# Strategies to Develop Na,K-ATPase-α4 Inhibitors as Male Contraceptives

**DOI:** 10.3390/ijms26125646

**Published:** 2025-06-12

**Authors:** Shameem S. Syeda, Gladis Sánchez, Jeffrey P. McDermott, Narsihmulu Cheryala, Henry L. Wong, Gunda I. Georg, Gustavo Blanco

**Affiliations:** 1 Department of Medicinal Chemistry, Institute for Therapeutics Discovery and Development, College of Pharmacy, University of Minnesota, Minneapolis, MN 55414, USA; syeda002@umn.edu (S.S.S.); chery002@umn.edu (N.C.); hlwong@umn.edu (H.L.W.); 2 Department of Cell Biology and Physiology, University of Kansas Medical Center, Kansas City, KS 66160, USA; gsanchez2@kumc.edu (G.S.); jmcdermott@kumc.edu (J.P.M.)

**Keywords:** sperm motility, sperm capacitation, male fertility, male contraception, ouabain, simplified steroidal analogs, and simplified small molecules

## Abstract

Male contraception remains an unmet need. Na,K-ATPase α4 (NKA α4), a specific Na⁺/K⁺ transporter of the sperm flagellum, is an attractive target for male contraception. NKA α4 is critical for sperm motility and fertility, and its deletion in male mice causes complete infertility. Our previous structure–activity relationship (SAR) studies on a cardenolide scaffold identified a highly selective, safe NKAα4 inhibitor, but its complex, heavily hydroxylated structure posed challenges for modification and optimization. To address this, we employed a structural simplification strategy to synthesize novel steroidal and non-steroidal analogs and examined their effects on NKAα4 inhibition and sperm motility. Both series reduced sperm motility (up to ~50%), with IC_50_ values in the picomolar range. Compounds **13** and **45** displayed specificities for NKAα4 over NKAα1, did not affect sperm viability, and showed no reversibility in vitro. Notably, **45**, featuring a hexahydronaphthalene core and a benzyltriazole moiety at C5, exhibited potent, highly selective NKAα4 inhibition, reduced sperm motility in vitro and in vivo, and blocked fertilization in vitro. This highlights **45** as a promising lead for non-hormonal male contraception and indicates that the newly generated series of compounds possess the key characteristics needed for further development as potential non-hormonal male contraceptive agents.

## 1. Introduction

Organisms often synthesize enzymes that catalyze the same basic biochemical reaction but have different molecular forms and properties, including distinct cell localization, specific regulation, and unique enzyme kinetics. This molecular heterogeneity highlights the versatility and refinement that cells have developed to perform their particular function [1]. An example of isozyme diversity is found for Na,K-ATPase (NKA), the active ion transport system of the plasma membrane of most cells, which exchanges intracellular Na^+^ for extracellular K^+^ [2]. Na,K-ATPase is a heterodimer of α and β subunits [2,3]. The α subunit is the main conduit through which Na^+^ and K^+^ cross the plasma membrane and has the enzymatic activity that allows it to hydrolyze ATP. The free energy released from the hydrolysis of ATP drives conformational changes in the NKA α molecule that are required to bind, transport, and release Na^+^ and K^+^ to the corresponding side of the cell plasma membrane [4]. The β polypeptide is also essential for NKA activity, participating in the folding, stability, and targeting of the α subunit to the plasma membrane [5,6]. The electrogenic movement of 3 Na^+^ out and 2 K^+^ into the cell by NKA contributes to establishing the plasma membrane potential typical of animal cells and the transmembrane Na^+^ gradient that fuels the secondary transport of other ions, nutrients, and water in and out of the cell [7,8]. 

NKA diversity is reflected in mammals by the existence of a family of genes that encode for four different α (α1, α2, α3, and α4) and three distinct β (β1, β2, and β3) polypeptides [9,10]. Additional molecular diversity is achieved by the association of the various α and β isoforms in different arrangements to produce multiple NKA αβ isozymes. These isozymes are expressed in a cell, tissue, and developmentally regulated manner, and they have distinct functional properties [11,12,13]. As demonstrated through the generation of mouse models in which different NKA isoforms were genetically deleted, NKA isozymes are not redundant but have a specific role in the tissues where they are expressed [2]. 

The NKAα4 isoform is restricted to male germ cells of the testis, and it is expressed after cell meiosis, reaching maximal levels in the differentiated spermatozoa [14,15]. Within sperm, NKAα4 is highly present at the plasma membrane of the flagellum, where it is essential for sperm motility and hyperactivation, a particular movement that sperm acquire during capacitation, and crucial for fertilization [16,17,18]. Importantly, the genetic deletion of NKAα4 in mice rendered the male animals infertile [19]. Conversely, the overexpression of NKAα4 enhances mouse sperm movement [20]. While sperm also express the NKAα1 isoform found in every cell, this isoform cannot compensate for NKA α4 [19]. Interestingly, patients with asthenospermia have been reported to have low NKA expression and activity levels [16]. This observation shows the highly specific role of NKAα4, which, by maintaining optimal intracellular Na^+^ ([Na^+^]_i_), cell membrane potential (V_m_), intracellular Ca^+2^ ([Ca^2+^]_i_), and H^+^ levels allow for efficient sperm motility [20].

The molecular diversity of NKA provides the opportunity to develop pharmacological interventions to inhibit NKAα4 function and control sperm fertility. NKAα4 represents an attractive target for contraception since it is expressed at the sperm cell surface, making it more accessible to compounds. It is exclusively present in sperm, which suggests that its inhibition will not affect other cells or tissues. Also, the expression of NKA α4 in the differentiated spermatozoa, and not its progenitor cells, should allow for temporary and not permanent infertility. These characteristics of NKAα4 prompted us to develop a contraceptive for men, which is in high need. While several contraceptive methods are currently available for women, a safe, effective, and reversible contraceptive for men is still not available. Therefore, developing a male contraceptive is of high priority to achieve a more comprehensive family planning strategy that will not place the responsibility of conception solely on the female.

**Figure 1:**
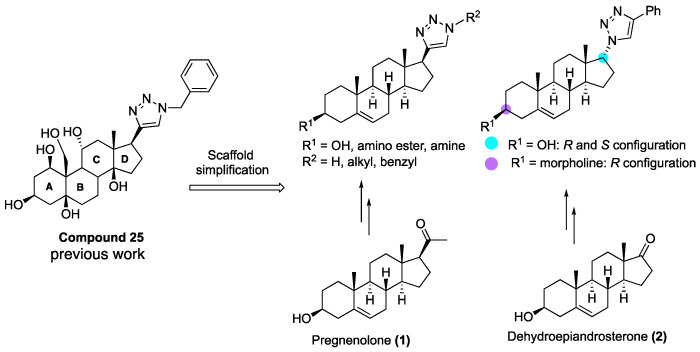
Design of simplified steroidal analogs.

We have previously reported that NKAα4 exhibits a particular sensitivity for ouabain, which is significantly higher than that of the NKAα1, α2, and α3 isoforms [15,21]. Ouabain is a natural product isolated from *Strophanthus gratus* and *Acokanthera schimperi*. It has a long history in treating heart failure and as an inhibitor for the specific determination of NKA activity in the laboratory setting [22]. The high affinity of NKAα4 for ouabain provides the opportunity to take advantage of this property to inhibit NKAα4 specifically. It has been shown that the dose-dependent inhibition of NKAα4 with relatively low concentrations of ouabain inhibits all parameters of sperm movement, including total and progressive motility, straight line, curvilinear, average path velocities, lateral head displacement, beat cross frequency, and linearity. The further inhibition of NKAα1 with higher ouabain concentrations does not cause an additional reduction in sperm motility [21,23], providing proof of principle for the specific inhibition of NKAα4 as a valid approach to block sperm function and for ouabain as an attractive molecular framework for male contraception. However, ouabain also binds to the NKAα1, α2, and α3 isoforms, and their inhibition is undesirable for a male contraceptive. Therefore, exploiting the high ouabain affinity site of NKAα4 for inhibition will require the development of new compounds with high selectivity for NKAα4. The feasibility of using this approach has been established in our previous work [24]. Thus, we have demonstrated that the ouabain derivative **25** (Figure 1) exhibits NKA isoform selectivity, effectively inhibiting NKAα4 at sub-nanomolar concentrations. It partially blocks sperm motility and hypermotility, interferes with oocyte fertilization in vitro, does not exhibit off-target effects in vitro, and reduces sperm motility when administered orally to rats [24,25]. However, its complex structure and extensive hydroxylation pose significant challenges for modification, limiting its pharmacological optimization. In addition, it has low permeability when tested in cell monolayers, which may complicate their absorption and perhaps their ability to reach the target cells [25].

Research in natural product-based drug discovery often faces challenges due to the structural complexity of compounds and the limited availability of natural sources. A practical approach to overcoming these obstacles is the design of structurally simplified analogs, a strategy that has proven successful in lead optimization and in developing several marketed drugs [26]. Insights from NKA inhibitors, including strophanthidin [24], highlight the potential of structural simplification in drug development. This strategy aims to retain biological activity while facilitating the synthesis of more accessible analogs with enhanced therapeutic potential. Building on these findings, we synthesized novel hybrid compounds featuring either a steroidal framework or a simplified structure, each incorporating a C17 triazole moiety that we had found to enhance the NKAα4 inhibitory potency and selectivity of our previously reported analog **25** (Figure 1) compared to ouabain [24,25]. This approach generated two distinct series: (1) simplified steroidal analogs and (2) non-steroidal small molecules. Here, we present our preliminary results on the synthesis and biological evaluation of these novel analogs, focusing on their effects on sperm motility and NKAα4 inhibition.

## 2. Results

### 2.1. Design and Structure–Activity Relationships (SAR) Exploration

To simplify the structure of previously reported compound **25** (Figure 1) and to identify its essential pharmacophore, we utilized pregnenolone (**1**) and dehydroepiandrosterone (**2**) as starting materials. They are readily available compounds that provide a reliable foundation for our synthetic strategy. Both possess a core ABCD steroidal ring system with 17 carbon atoms, offering a flexible scaffold for designing and synthesizing simplified steroidal analogs (Figure 1). We developed simplified steroid analogs **13–15** and **30** by incorporating a C-17 triazole moiety as the key structural element. To further investigate SAR and assess the positional relevance of the triazole ring, we synthesized reversed triazole analogs **24** and **26** by interchanging the azide and alkyne partners. These analogs differ in their stereochemistry at C17. L-lysine ester **19** and morpholine analog **26** were synthesized by modifying the C3 hydroxyl group of compounds **13** and **24** to enhance solubility. Incorporating polar L-lysine amino esters and morpholine moieties was expected to improve solubility compared to compounds **13** and **24**.

**Figure 2:**
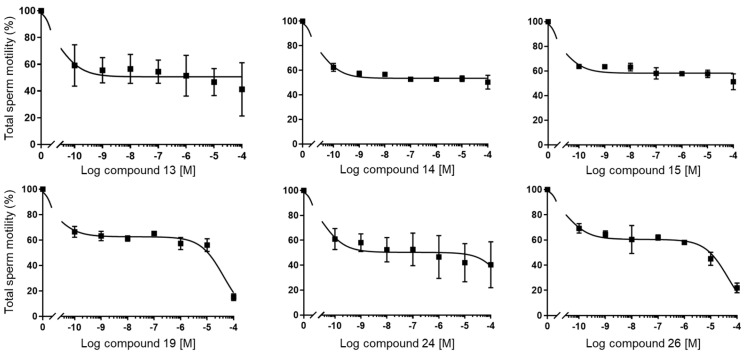
Effect of simplified steroidal analogs on mouse sperm total motility. Sperm was isolated from the cauda epididymis and treated in the absence and presence of the indicated concentrations of each compound. After one hour of incubation, sperm motility was determined by CASA. Values are expressed as the mean ± SEM of three determinations.

To characterize the SAR of these compounds, we first evaluated their effects on total sperm motility in vitro. Sperm isolated from the cauda epididymis of mice were incubated with the test compounds in modified Whitten’s medium under non-capacitating conditions. Motility was assessed using a computer-assisted sperm analysis (CASA) system. Figure 2 shows the dose-dependent effect curves for the simplified steroidal analogs. While sperm motility assays only provide a general estimation for IC_50_ values, especially at low concentrations of the compounds, it is clear that the compounds exerted an effect at sub-nanomolar concentrations. Also, although these scaffolds did not reach a full blockage of sperm motility, the inhibition that they caused (40–50%) provides proof of principle for their activity on sperm function.

Compounds **13**, **14**, and **15** reduced mouse sperm total motility by approximately 40–45%, displaying a monotonic inhibitory response with an IC_50_ in the sub-nanomolar range (Table 1). These compounds have variations in the triazole *N*-alkyl substitution; however, these structural changes did not significantly affect their inhibitory potency. In contrast, other compounds in this series, such as **19**, **24**, and **26,** displayed biphasic inhibition. They exhibited an initial inhibitory effect in the sub-nanomolar range, followed by a secondary phase that required micromolar concentrations (Table 1). Among these, **19**, which carries a L-lysine ester, was the weakest inhibitor, reducing sperm motility by ~37%. Compound **24**, featuring a reversed triazole scaffold, showed 50% inhibition of sperm motility. Compound **26**, which shares the reversed triazole core and includes a morpholine substituent at C3, retained potency and reduced sperm motility by approximately 40%, similar to other analogs in this series. Analog **30**, bearing inverted C17 stereochemistry, was not evaluated due to inadequate solubility.

**Figure 3:**
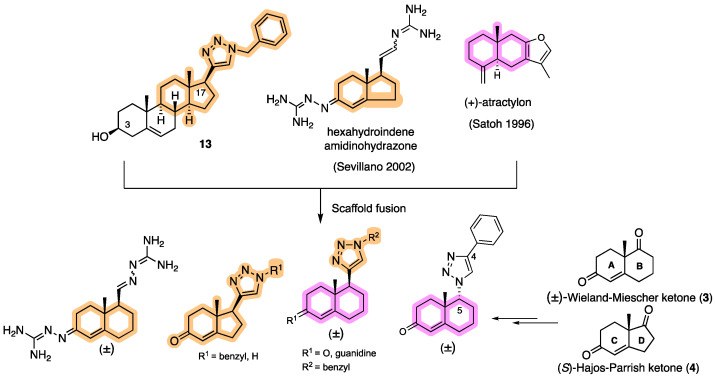
Design of simplified non-steroidal small molecule analogs of **13** by adapting structural features from hexahydroindene amidinohydrazone [28] and (+)-atractylon [29].

Compound **13** was selected for further evaluation due to its structural similarity to our previous lead compound **25**, which carries a C3 hydroxyl and a C17 benzyltriazole while maintaining potent NKAα4 activity [24]. These observations suggest that the hydroxyl groups in compound **25** may not be essential for potency. Given that hexahydroindene amidinohydrazone (**41**, Table 2) [27,28] and the natural product atractylone [29] are known Na,K-ATPase inhibitors, we pursued further simplification of the steroidal core by using a hexahydroindene skeleton (representing the C and D rings) and a hexahydronaphthalene core (representing the A and B rings), as illustrated in Figure 3. 

**Figure 4:**
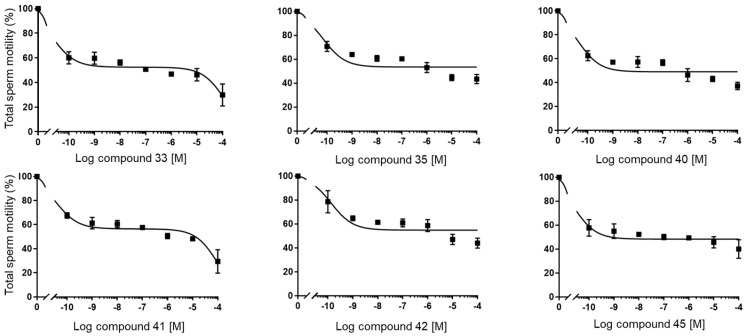
Effect of non-steroidal small compounds on mouse sperm total motility. Sperm was isolated from the cauda epididymis and treated in the absence and presence of the indicated concentrations of each compound. After 1 h of incubation, sperm motility was determined by CASA. Values are expressed as the mean ± SEM of three determinations.

In line with our design strategy, we synthesized non-steroidal small molecules **33**, **35**, **39**–**42**, and **45** bearing hexahydroindene or hexahydronaphthalene scaffolds from commercially available (±)-Wieland–Miescher ketone (**3**) and (*S*)-Hajos–Parrish ketone (**4**) and evaluated their effects on sperm motility. Figure 4 shows the dose-dependent effect curves of these simplified analogs.

Table 2 summarizes the maximal sperm motility inhibition at nanomolar range concentrations of the compounds and IC_50_ values in mouse sperm for the tested compounds. First, we introduced a triazole moiety with a benzyl substituent (**33**) or without it (**35**) on the hexahydroindene scaffold. The benzyl-triazole analog **33** exhibited 48% motility inhibition, and the unsubstituted triazole analog **35** showed 47% inhibition. We then incorporated a benzyl-substituted triazole into the hexahydronaphthalene scaffold to generate compound **39**, but its poor solubility precluded it from testing. A guanidine moiety was introduced in compound **40** to address solubility issues. This modification also showed activity like **33**. We also synthesized the known NKA inhibitor hexahydroindene-amidino guanidine analog **41** and the hexahydronaphthalene analog **42**, which exhibited 44% and 49% motility inhibition, respectively. The reversed triazole analog **45** demonstrated high potency compared to other compounds in the series, showing a maximum of 51% sperm motility inhibition with an IC_50_ of 48 pM. In motility inhibition, it outperformed the simplified steroidal analog **13**. Based on its favorable physicochemical and drug-like properties (Table 3), compound **45** was selected for further investigation. SAR studies on the simplified small molecules suggest that an intact steroid skeleton may not be essential for NKAα4 inhibitory activity. Instead, a truncated steroidal core design represents a promising strategy for developing novel male contraceptive agents, particularly given the enhanced chemical accessibility and ease of synthesis offered by analogs such as **45**.

Compounds **13** and **45** were selected for further studies, in which **13** features a benzyl triazole at C17, while **45** contains a reversed triazole with a C4-phenyl substitution. Both compounds were assessed through a series of assays to evaluate their cell viability, inhibitory potency, selectivity for NKAα4 and α1, reversibility, permeability, and solubility.

### 2.2. Sperm Viability

**Figure 5:**
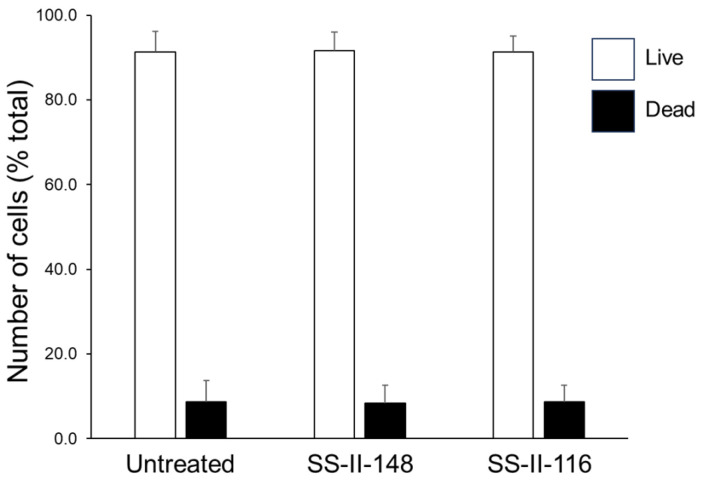
Cell viability after treatment with compounds **13** and **45**. Mouse caudal epididymal sperm was subjected to a medium not supporting capacitation to 10 µM of **13** and **45**. After one hour, sperm was subjected to the Live/Dead assay as per the manufacturer’s recommendations. Cells were counted as viable/non-viable and expressed as the mean ± SEM of three determinations.

To assess whether **13** and **45** affected sperm viability, we treated mouse sperm with the compounds for one hour at 10 µM and determined the number of live and dead cells in the sample. Figure 5 shows that the viability of the cells treated with **13** and **45** was the same as that of the untreated controls, indicating that our compounds are not spermicides but rather affect the function of the cells (Figure 5).

### 2.3. NKA Activity and Their Selectivity for the NKA α4 Isoform

**Figure 6:**
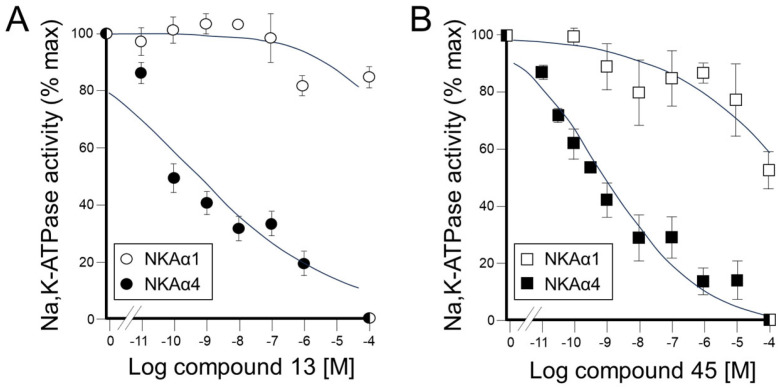
Selectivity of **13** and **45** on NKAα4 over the NKAα1 isoform. Dose–response curves for the inhibition of NKA activity by compounds **13** (**A**) and **45** (**B**) were determined on rat NKAα1β1 and α4β1 produced in Sf9 insect cells. The hydrolysis of ATP in the presence of saturating concentrations of Na^+^, K^+^, and Mg^2+^ was measured using γ[^32^P]-ATP. The curves represent the best fit of the experimental data to a single population of binding sites. Each value is the mean ± SEM of three independent experiments.

We next determined the capacity of the compounds to inhibit NKA activity and their selectivity for the NKA α4 isoform. As shown in Figure 6, dose–response curves for the inhibition of NKA activity by both **13** and **45** showed a specificity of effect toward NKA α4, with IC_50_ values for inhibition in the nanomolar range compared to low micromolar for NKA α1. The IC_50_ values for each isoform are shown in Table 3.

### 2.4. Reversibility

**Figure 7:**
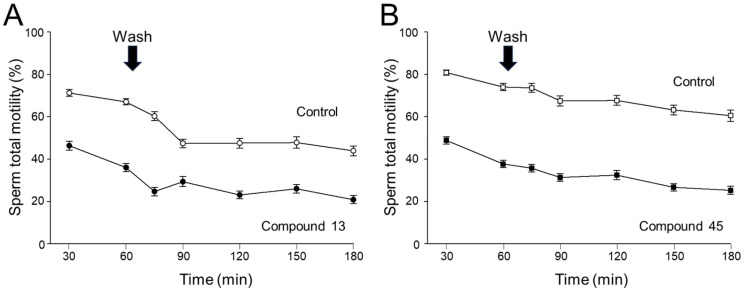
Reversibility of the effect of compounds **13** and **45**. Mouse epididymal sperm were treated in the absence (untreated control) or presence of **13** (**A**) or **45** (**B**) for one hour. Then, the sperm was washed, and motility was measured before and after washing the cells using CASA. Sperm movement was followed during the indicated times. Values are expressed as the mean ± SEM of three determinations. Samples treated with **13** and **45** were statistically different from the untreated controls, with *p* < 0.05.

An important goal for the development of agents to be used for contraception is to assess the reversibility of their effect on sperm. To achieve this, we treated mouse sperm in the absence and presence of either **13** or **45** and measured epididymal sperm motility before and after washing the cells with fresh medium. As shown in Figure 7, retrieving the compounds from the medium did not significantly affect sperm motility in the treated samples, indicating that, at least for the two hours of the experiment, the effect of the compound on sperm was stable. Although these experiments do not directly quantify the reversibility of the compounds to their sperm target, they suggest that the effect of **13** and **45** is maintained over time.

### 2.5. The Potencies, Permeability, and Solubility of Compounds ***13*** and ***45***

Table 3 summarizes the potencies, permeability, and solubility of compounds **13** and **45**, which displayed potent inhibitory activity against NKAα4 compared to NKAα1. While compound **45** exhibited good permeability and solubility, compound **13** showed poor permeability and solubility, limiting its usefulness as a pharmacological tool despite its potency and selectivity. Consequently, **13** was not pursued further. In contrast, the potent activity, selectivity, and favorable ADME properties make **45** a good prospect for further pharmacological development.

### 2.6. In Vitro ADME and Safety Profile Evaluation of ***45***

Before in vivo evaluation, compound **45** was assessed for its in vitro ADME properties and safety. Caco-2 permeability assays showed efficient bidirectional transport 24 × 10^−6^ cm/s, indicating good epithelial permeability (Table 3). As shown in Table 4, metabolic stability studies revealed moderate stability at 60 min in human hepatocytes (39%) but lower stability in rat and mouse hepatocytes (10%), leading to a shorter half-life (T_1_/_2_) and higher clearance in these species. This observation aligns with in vivo pharmacokinetic data, suggesting greater metabolic instability and faster elimination in mice, potentially requiring relatively high oral doses. Compound **45** was evaluated for cardiovascular risk (via human Ether-à-go-go-Related Gene (hERG) inhibition) and genotoxicity (via the Ames test) as part of the safety assessment. The compound showed no hERG inhibition at concentrations up to 30 μM (IC_50_ > 30 μM) and was negative in the Mini Ames test, indicating no genotoxicity.

### 2.7. Pharmacokinetics Study of ***45***

**Figure 8:**
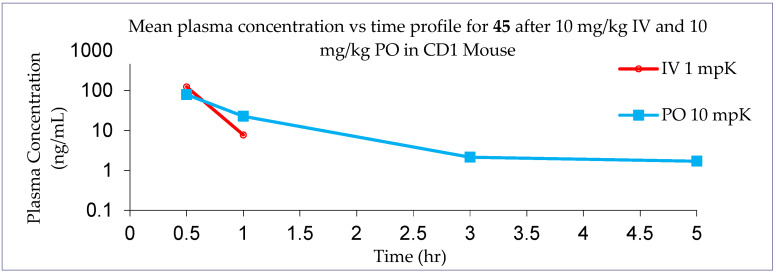
Pharmacokinetic profile of **45** in CD1 mice.

Pharmacokinetic experiments were carried out with compound **45** in CD-1 mice. The compound was administered at a dose of 10 mg/kg via both intravenous and oral routes. Blood samples were collected and analyzed using an LC-MS/MS system to determine drug concentration (Figure 8). Although **45** rapidly reached its maximum concentration following intravenous administration, it exhibited a high clearance rate (297 μL/min/kg in mice), consistent with its low metabolic stability in hepatocytes. Furthermore, the oral bioavailability was poor, measured at only 14%, indicating that oral administration is not a viable option for in vivo experiments (Table 5).

### 2.8. In Vivo Evaluation of ***45*** on Sperm Motility

**Figure 9:**
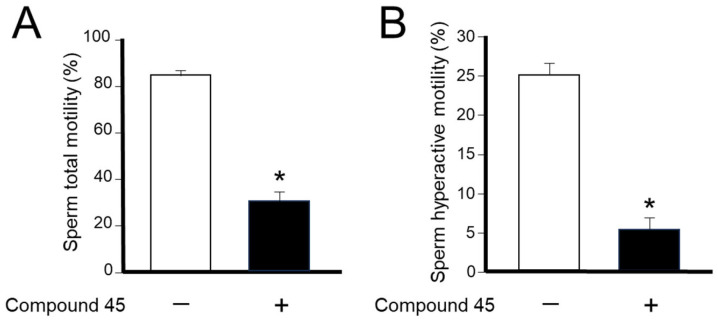
Effect of compound **45** on total (**A**) and hyperactive (**B**) sperm motility in mice in vivo. **45** was administered via intraperitoneal injection at a dosage of 40 mg/kg of body weight for 3 days. Total and hyperactive sperm motility was assessed using sperm from the cauda epididymis. Hyperactive motility was evaluated after incubating the cells in a medium that supports sperm capacitation for 1 h. Sperm motility was analyzed using CASA. Bars represent the mean ± SEM of three experiments. Values significantly different from untreated controls are indicated with an asterisk where *p* ≥ 0.05.

To determine whether our compounds had in vivo activity, we tested the effect of **45** after intraperitoneal administration to mice. Compound **45** was injected at a dose of 40 mg/kg for three days. This procedure is based on our previous protocol used for ouabain derivatives [24]. After treatment for 3 days, the animals were sacrificed, sperm was isolated from the cauda epididymis, and sperm motility was assessed using CASA. Compound **45** inhibited total sperm motility by approximately 60% (Figure 9A). Significantly, it also reduced hyperactive motility, which is the pattern of motility that cells adopt during capacitation (Figure 9B). These results demonstrate that compound **45** effectively inhibits sperm motility, with ~50% inhibition in vitro and ~60% inhibition in vivo following administration.

### 2.9. Evaluation of ***45*** in an In Vitro Fertilization Assay

**Figure 10:**
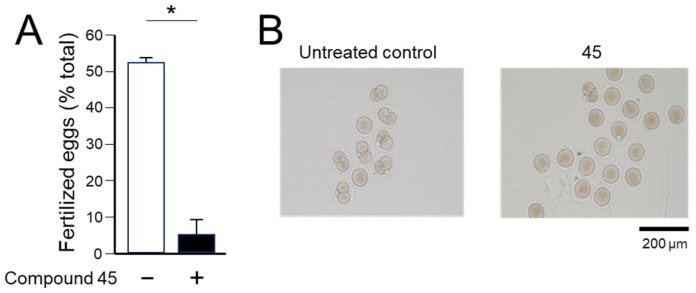
Effect of **45** on in vitro fertilization. Mouse caudal epididymal sperm was treated with compound **45** at 10 µM. The cells were washed with medium and then applied to eggs placed in 6-well plates. Sperm and oocytes were incubated in Cook’s medium to facilitate fertilization. Oocytes were separated and incubated overnight, and the development of two-cell embryos was assessed. (**A**) The fertilization rate is expressed as the number of eggs fertilized as a percentage of the total eggs in the sample. Bars represent the mean ± SEM. Statistical differences between untreated and control samples are indicated with an asterisk, with *p* < 0.001. (**B**) Representative light microscopy images of oocytes and two-cell embryos.

To more directly obtain proof of the principle of the potential contraceptive capability of a small ouabain derivative, we tested the effect of **45** in an in vitro fertilization assay. Therefore, after treating mouse caudal epididymal sperm with **45** for one hour, cells were washed and used to fertilize eggs in vitro. The washing of the sperm was performed to prevent any effect of the compound on the egg. As shown in Figure 10, **45** caused a significant decrease in the number of fertilized eggs compared to the untreated controls.

### 2.10. Chemistry

**Scheme 1:**
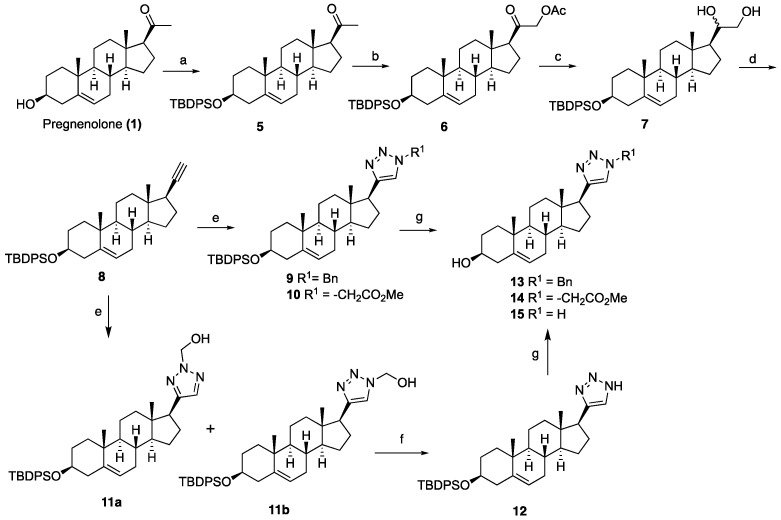
Synthesis of simplified-steroid analogs from pregnenolone. Reagents and conditions: (a) TBDPSCl, imidazole, DCM, rt, 4 h, 85%. (b) (i) Pb(OAc)_4_, BF_3_·OEt_2_, toluene, rt, 4 h, 63%. (c) LiAlH_4_, THF, 0 °C to rt, 30 min, 67%. (d) (i) NaIO_4_, THF:H_2_O (8:2), rt, 1 h; (ii) CBr_4_, TPP, DCM, 0 °C to rt, 30 min; (iii) BuLi, THF, −78 °C, 1 h, 70% over three steps. (e) Benzyl azide, methyl 2-azidoacetate or HCHO/NaN_3_, CuSO_4_·5H_2_O (20 mol%), sodium ascorbate (40 mol%), 24 h, **9** (80%), **10** (68%), **11a+b** (65%). (f) MnO_2_, CHCl_3_, reflux, 20 h, 82%. (g) 1 N HCl in MeOH, rt, 5 h, **13** (80%), **14** (70%), **15** (67%).

**Scheme 2:**
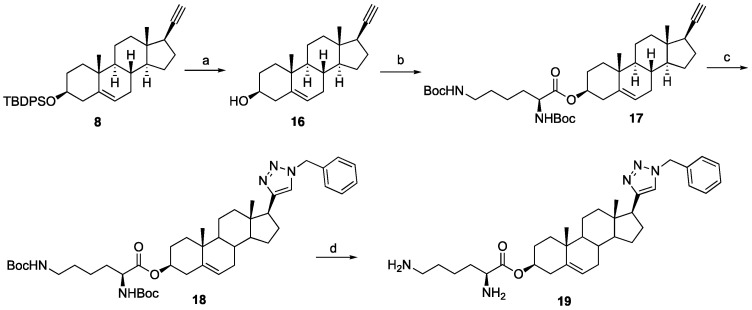
Synthesis of L-valine lysine ester analog 19. Reagents and conditions: (a) 1 N HCl in CH_3_OH, rt, 5 h, 63%; (b) *N*,*N*-diBoc-L-lycine, DCC, DMAP, THF, 18 h, rt, 76%; (c) benzyl azide, CuSO_4_·5H_2_O (20 mol%), sodium ascorbate (40 mol%), DMF:H_2_O (2:1), 12 h, rt, 78%; (d) 2 N HCl in Et_2_O, 18 h, 71%.

**Scheme 3:**
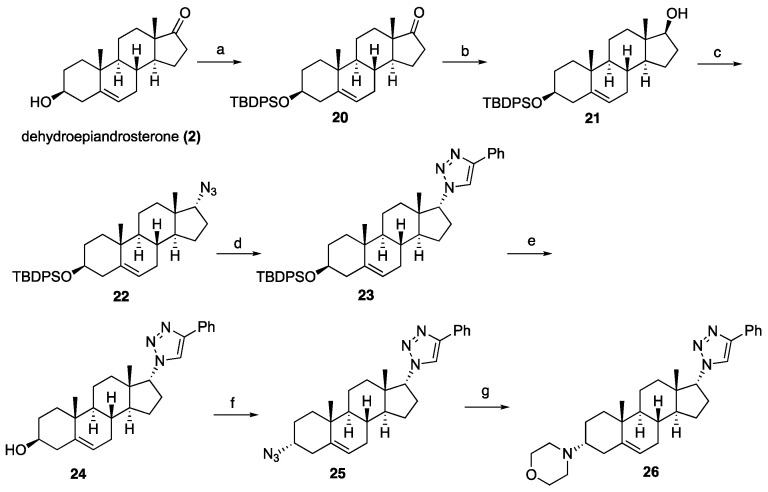
Synthesis of reversed triazole analogs from dehydroepiandrosterone (2). Reagents and conditions: (a) (i) TBDPSCl, imidazole, DCM, rt, 5 h, 98%; (b) NaBH_4_, CH_3_OH:THF (1:1), 0 °C to rt, 1 h, 88%; (c) DEAD, TPP, DPPA, THF, 0 °C to rt, 10 h, 71%; (d) phenylacetylene, CuSO_4_·5H_2_O (20 mol%), sodium ascorbate (40 mol%), DMF:H_2_O (1:1), 12 h, rt, 67%; (e) 1 N HCl in MeOH, rt, 5 h, 72%; (f) DEAD, TPP, DPPA, THF, 0 °C to rt, 10 h, 58%; (g) (i) TPP, THF, rt, 1 h then H_2_O, rt, 10 h, (ii) 2-bromoethyl ether, K_2_CO_3_, toluene, 24 h, 77% for two steps.

**Scheme 4:**
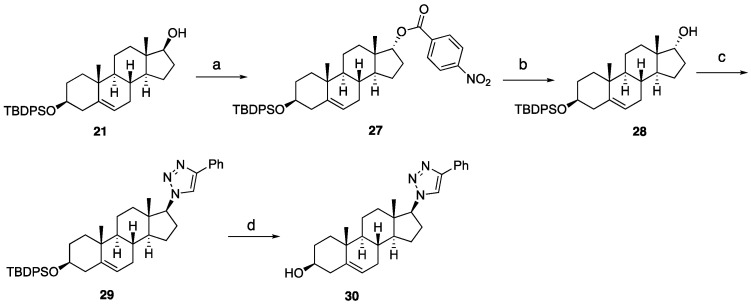
Synthesis C17 inverted triazole analog 30. Reagents and conditions: (a) DEAD, TPP, 4-nitrobenzoic acid, THF, 0 °C to rt, 12 h. (b) K_2_CO_3_, CH_3_OH, rt, 18 h, 53% for two steps. (c) (i) DEAD, TPP, DPPA, THF, 0 °C to rt, 12 h, 43%; (ii) phenylacetylene, CuSO_4_·5H_2_O (20 mol%), sodium ascorbate (40 mol%), DMF:H_2_O (1:1), 12 h, rt, 58%. (d) 1 N HCl in MeOH, rt, 5 h, 65%.

To determine the role of hydroxyl groups on the steroidal core of ouabain and compound **25** from our previous inhibitor, we designed and synthesized simplified steroidal analogs from pregnenolone (**1**) and dehydroepiandrosterone (**2**), as outlined in Scheme 1, Scheme 2, Scheme 3 and Scheme 4. As depicted in Scheme 1, the synthesis began with the protection of the C3 hydroxyl group in compound **1** as silyl ether **5**, followed by the acetoxylation of **5** to yield **6**. Subsequent reduction with LiAlH_4_ produced diol **7**. Oxidative cleavage of diol **7**, followed by one-carbon homologation using TPP and CBr_4_, and subsequent treatment with BuLi, provided the key alkyne intermediate **8**. The alkyne **8** underwent cycloaddition with azides, forming silyl-protected triazoles (**9**–**11**). Silyl-protected NH-triazole analog **12** was synthesized via a one-pot, three-component copper-catalyzed reaction of alkyne **8**, sodium azide (NaN_3_), and formaldehyde [30]. This reaction produced an inseparable mixture of regioisomeric hydroxymethyl triazoles **11a** and **11b**, which was subsequently subjected to oxidative cleavage of the hydroxymethyl group to yield the corresponding NH-triazole **12**. Finally, the silyl-protected triazoles **9**, **10**, and **12** were deprotected to obtain the simplified steroid analogs **13**–**15**. The C3 hydroxyl group of **13** was modified to generate lysine ester analog **19** to enhance solubility, as shown in Scheme 2. For the synthesis of this analog, silylated alkyne **8** was deprotected to yield **16**, which was then coupled with *N*,*N*-diBoc-lysine to afford the protected lysine ester **17**. The triazole ring was subsequently installed on **17** via click chemistry with benzyl azide. Finally, deprotection of **18** yielded the lysine ester triazole analog **19**.

To further explore SAR and assess the positional relevance of the triazole ring, we synthesized reversed triazole analogs by interchanging the azide and alkyne partners, as illustrated in Scheme 3. The C3 hydroxyl group of dehydroepiandrosterone (**2**) was protected as a silyl ether **20**, followed by keto reduction to provide **21.** The Mitsunobu azidation of **21** generated the C17 azide **22** that was subsequently coupled with phenylacetylene via click chemistry to install the triazole at C17, yielding **23**. Finally, silyl deprotection afforded the target compound **24**. Further modifications were introduced by replacing the C3 hydroxyl group of **24** with morpholine. To prepare this analog, compound **24** was subjected to Mitsunobu azidation to obtain the C3 azide **25**, which was reduced using Staudinger conditions followed by alkylation with 2-bromoethyl ether to furnish target compound **26.**

The inversion of configuration at C17 of compound **24** was achieved as detailed in Scheme 4. The transformation began with a Mitsunobu reaction with **21**, yielding the nitrobenzoate intermediate **27**, which was subsequently hydrolyzed to afford inverted alcohol 28. Compound **28** was then converted into the silylated C17-inverted triazole **29** through a two-step sequence involving a Mitsunobu azidation and a click reaction. Finally, silyl deprotection provided the target compound **30**.

**Scheme 5:**
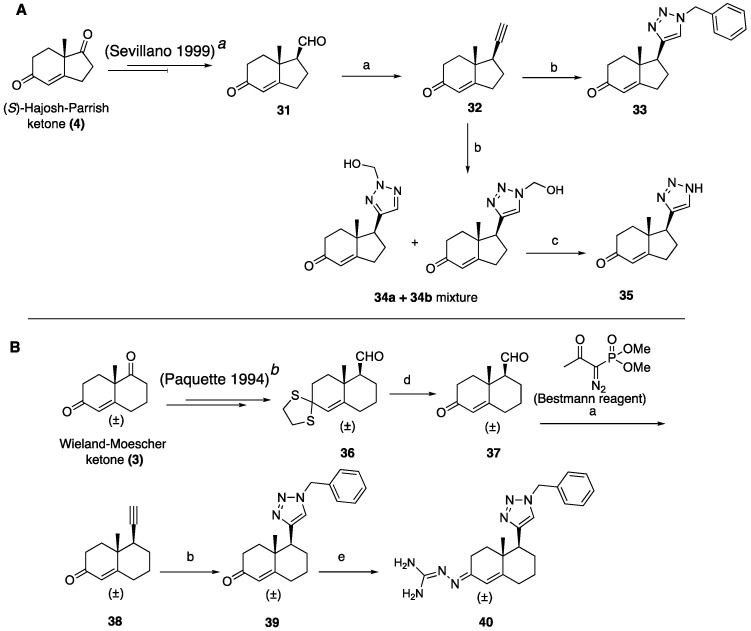
(**A**). Synthesis of hydroindene and (**B**) Synthesis of tetrahydronaphthalene triazole analogs. Reagents and conditions: (a) Bestmann reagent, K_2_CO_3_, CH_3_OH, 2 h, (**32**, 65%), (**38**, 68%); (b) benzyl azide or NaN_3_, CuSO_4_·5H_2_O (20 mol%), sodium ascorbate (40 mol%), DMF:H_2_O (1:1), 12 h, rt, (**33**, 67%), (**34a+34b**, 54%), (**39**, 51%); (c) MnO_2_, CHCl_3_, reflux, 60%; (d) PhI(O_2_CCF_3_)_2_, CH_3_CN (10:1), 10 min, rt, 92%; (e) aminoguanidine hydrochloride, 2 N HCl, EtOH, reflux, (**40**, 71%). Compounds **31***^a^* and **39***^b^* were synthesized following the method reported in references [27] and [31], respectively.

**Scheme 6:**
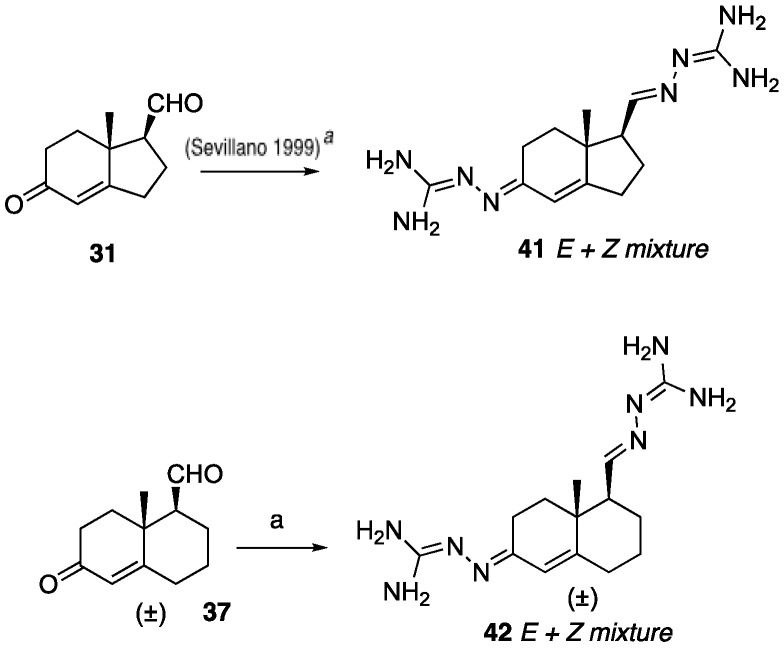
Synthesis of hydroindene and tetrahydronaphthalene triazole analogs. Reagents and conditions: (a) aminoguanidine hydrochloride, 2 N HCl, EtOH, reflux, 80%. Compounds **41***^a^* was synthesized following the method reported in reference [27].

**Scheme 7:**
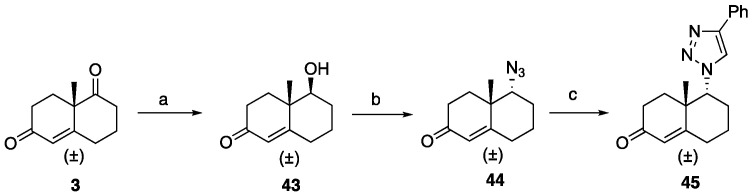
Synthesis of revered triazole tetrahydronaphthalene analog **45**. Reagents and conditions: (a) NaBH_4_, EtOH, 0 °C, 5 min, 82%; (b) DEAD, TPP, DPPA, THF, 0 °C-rt, 10 h, 50%; (c) phenylacetylene, CuSO_4_·5H_2_O (20 mol%), sodium ascorbate (40 mol%), DMF:H_2_O (1:1), 12 h, rt, 71%.

We synthesized simplified non-steroidal small molecules using the readily available Wieland–Miescher (**3**) and Hajos–Parrish (**4**) ketones, as illustrated in Scheme 5, Scheme 6 and Scheme 7. The introduction of the triazole ring was achieved according to Scheme 5. Keto-aldehyde **31** was prepared from **4** using a known procedure [27], while keto-aldehyde **37** was synthesized by the deprotection of **36**, which was obtained following a previously reported protocol from racemic Wieland–Miescher ketone (**3**) [31]. Keto-aldehydes **31** and **37** were treated with the Ohira–Bestmann reagent to yield alkynes **32** and **38**, which were subjected to click chemistry with benzyl azide to obtain **33** and **39**. Triazole **35** was prepared by click chemistry between alkyne **32**, NaN_3,_ and formaldehyde to furnish hydroxymethyl substituted triazole as a mixture of **34a** and **34b** in a 7:3 ratio. Subsequent cleavage with MnO_2_ yielded compound **35** (5A). Compound **40** was prepared from **39** by introducing the polar guanidine functionality to improve solubility (5B).

The known NKA inhibitor hexahydroindene amidinohydrazone **41** [27,28] was synthesized from keto-aldehyde **31** using a reported procedure. Additionally, we prepared hexahydronaphthalene amidinohydrazone **42** from keto-aldehyde **37** using aminoguanidine, as illustrated in Scheme 6.

Compound **45**, a reversed triazole hexahydronaphthalene analog, was synthesized using a three-step process: the reduction of ketone **3** to obtain alcohol **43**, followed by Mitsunobu azidation to form azide **44**, and a click reaction to yield **45**, as shown in Scheme 7.

## 3. Discussion

The need for effective contraceptive methods remains critical due to the high rate of unintended pregnancies and the rapidly growing global population [32,33,34,35]. While numerous contraceptive options exist for women [36], a safe, effective, and reversible male contraceptive remains unavailable. Developing a male contraceptive would significantly contribute to family planning and reproductive health [37]. With this objective in mind, we designed novel ouabain analogs that inhibit the NKAα4 isoform, interfering with sperm function and fertility [24]. Our approach builds on previous findings demonstrating that sperm-specific NKAα4 plays a crucial role in sperm motility and capacitation, making it an attractive target for male contraception. 

Furthermore, NKAα4 is pharmacologically targetable due to its high affinity for ouabain [24,25]. While ouabain’s specificity for NKA enables the selective inhibition of NKAα4, its toxic effects and those of other natural cardenolides [38,39,40] necessitate the development of alternative chemical scaffolds. Prior studies have demonstrated that synthetic ouabain derivatives show potential as pharmacological agents to regulate sperm function [24]. However, their complex structure and numerous hydroxyl groups present substantial challenges for further research modifications. 

In this study, we employed a structural simplification strategy to develop steroidal and non-steroidal analogs inspired by the inhibitory activity of strophanthin, bis guanidine compounds, and the natural product atractylon against NKA [29]. Both compound series effectively inhibited total sperm motility, a key function regulated by NKAα4. While their effects varied, these compounds reduced sperm movement in vitro by 20–50%. This level of inhibition is lower than that obtained through genetic ablation studies, where the complete loss of NKAα4 expression abolished nearly all sperm motility. The reason why our compounds do not entirely suppress sperm movement remains unclear. It is possible that the relatively short time of our in vitro experiments, as compared to the complete knockout of NKAα4, accounts for these differences. Importantly, however, the observed level of sperm motility inhibition achieved by the compounds aligns with the World Health Organization’s criteria for male infertility [41], reinforcing their potential as potential pharmacological contraceptive agents. 

Among the compounds studied, **13** and **45** demonstrated exceptional selectivity for NKAα4 over the ubiquitously expressed NKAα1 isoform, with IC_50_ values for NKAα4 activity in the sub-nanomolar range. This high selectivity is a desirable characteristic for developing a pharmacological male contraceptive. Another important property that a contraceptive agent should present is reversibility of effect. This was taken into consideration when selecting our target for male contraception. Being expressed after meiosis, NKα4 is present in spermatids and mature spermatozoa [14]. Therefore, its inhibition should not affect the primordial male germ cells, allowing spermatogenesis to progress normally so that no permanent sterility will occur. On the other hand, to be an effective contraceptive, compounds should not rapidly leave their sperm target once bound. Long-term in vitro evaluations are limited by the natural decline in sperm motility over time. However, our results indicate that the effects of **13** and **45** were not reversible, at least within the timeframe of mouse sperm motility assessments. This prolonged activity is an advantageous property for our compounds. Due to its poor permeability and solubility, **13** was unsuitable for further studies. Therefore, only compound **45** was pursued for further evaluation. 

Importantly, our experiments demonstrated that the sperm motility inhibitory action of **45** also takes place after administration in vivo. Pharmacokinetic studies and ADME evaluations of **45** revealed good solubility and permeability and no hERG liability. However, the compound exhibited low metabolic stability and poor oral bioavailability (14% in mice). This suggests that a relatively high dose may be required to achieve a sustained in vivo effect on sperm motility. To ensure that the compound achieved relevant systemic levels, we injected compound **45** intraperitoneally. At present, the effect of the compound via other routes of administration is unknown, and this will be explored during the further development of the compound. Nevertheless, our data provides proof of principle for the in vivo effect of small NKAα4 inhibitors.

Compound **45** significantly inhibited sperm motility both in vitro and in vivo. Three days after intraperitoneal administration, sperm movement was reduced by approximately 60%, suggesting that the compound effectively reaches its target cells and retains its activity after sperm isolation from the mouse epididymis. Our in vivo experiments showed that **45** affected sperm motility and hypermotility, indicating that it interfered not only with sperm function under non-capacitated conditions but also with sperm capacitation. Therefore, **45** has a dual effect, not only decreasing the capacity of sperm to swim and reach the egg but also reducing the hyperactivation needed for sperm to penetrate the egg zona pellucida and fertilize the egg.

While it is apparent that **45** can reach the spermatozoa, it is unclear whether this occurs at the testis level or later in the male reproductive tract. The blood–testis barrier is known to tightly restrict the passage of molecules into the seminiferous tubule lumen. Instead, the epididymal epithelium is relatively more permeable to circulating compounds. Additional experiments will be required to determine the distribution of our compounds in different regions of the male reproductive tract; however, our study provides promising information for using these compounds as effective blockers of sperm function in vivo.

A key proof of principle for the effect of our compounds as blockers of sperm function is the reduction in fertilization that **45** caused in in vitro fertilization assays. Compound **45** diminished the formation of two-cell embryos by approximately 90%. This is of high significance, especially considering that IVF assays maximize the conditions for fertility in experimental conditions in a controlled environment. The relatively higher effect of **45** in the IVF assays compared to the effect on sperm motility or sperm hyperactivation indicates that **45** partially affects different sperm functional parameters. The combined effects result in a stronger action on fertilization, the ultimate goal for a desired male contraceptive.

## 4. Materials and Methods

### 4.1. Synthetic and Characterization Data

#### 4.1.1. General Information

All chemicals and reagents were purchased from commercial sources and used directly without further purification. Anhydrous solvents (CH_3_CN, EtOH, *^i^*PrOH) were purchased from Sigma-Aldrich (Milwaukee, WI, USA) and were used without further purification. Dry solvents (DMF, THF, DCM) were dispensed under nitrogen from a solvent purification system. All non-aqueous reactions were performed under an atmosphere of nitrogen in oven-dried glassware. Reaction progress was monitored by thin layer chromatography using silica gel plates (60 F_254_), and eluted TLC plates were visualized with UV light (254 nm) or I_2_. The products were isolated and purified by flash column chromatography. Yields were unoptimized. NMR experiments were performed on a 400/100 MHz instrument. NMR spectra were processed using the MestReNova program 14.1.1. Chemical shifts are reported as ppm referenced to CDCl_3_ (7.26 ppm for ^1^H, 77.0 ppm for ^13^C) and CD_3_OD (3.31 and 4.87 ppm for ^1^H, 49.1 ppm for ^13^C). ^1^H NMR coupling constants (*J*) are expressed in Hz, and multiplicity is described as follows: s = singlet; d = doublet; t = triplet; q = quartet; p = pentet; br = broad; m = multiplet. The compounds’ purity was analyzed with UPLC/MS. The UPLC analyses and mass spectra (LC-MS) were obtained on the Waters ACQUITY system (Waters, Milford, CT, USA) with a QDa Mass Detector with ESI inlet and UV PDA detector. The Waters UPLC BEH C18 (2.1 × 50 mm, 1.7 µm), column was used at a 40 °C temperature. The sample was dissolved in between 200 µL and 600 µL of DMSO, depending on the solubility of the sample. The sample was then diluted to approximately 0.1 mg/mL in MeOH for injection into the LCMS. The LCMS parameters were as follows: mobile Phase A (10 mM ammonium bicarbonate in water), mobile Phase B (ACN or MeOH), a flow rate of 0.6 mL/min, injection volume 7.5 µL, run time 6.0 min. The Gradient Operation was as follows: Hold at 95:5 A: B for 0.5 min. Linear gradient to 5:95 A: B for 3 min. Hold at 5:95 A: B for 0.5 min. Linear gradient to 95:5 A: B for 0.5 min. Hold at 95:5 A: B for 1.5 min. The mass detector ran a positive scan from 150 to 1000 Da. The PDA quantified the peaks at 254 nm. The low solubility of the steroidal analogs prevented the determination of their purity using standard HPLC methods. However, all tested compounds demonstrated greater than 95% purity as assessed by NMR. Their purity was sufficient for testing, as determined by NMR. All tested simplified small molecules are over 95% pure by HPLC, except for compounds **41** and **42.** The purity of compound **41**, assessed by NMR, was adequate for testing, while compound **42** exhibited 93% purity by HPLC. Keto aldehyde **31** [27,28], dithiane-protected aldehyde **36** [31], and hexahydroindene amidinohydrazone (**41**) were synthesized according to the published procedures [27,28].

#### 4.1.2. Experimental Procedures

1-((3*S*,8*S*,9*S*,10*R*,13*S*,14*S*,17*S*)-3-((*tert*-Butyldiphenylsilyl)oxy)-10,13-dimethyl-2,3,4,7,8,9,10,11,12,13,14,15,16,17-tetradecahydro-1*H*-cyclopenta[*a*]phenanthren-17-yl)ethan-1-one (**5**): To a solution of pregnenolone (**1**, 4.00 g, 12.65 mmol) in DCM (100 mL) at 0 °C, imidazole (2.58 g, 38.0 mmol) and TBDPSCl (4.80 mL, 19.0 mmol) were added. After being stirred at room temperature for 4 h, the reaction mixture was quenched with saturated aqueous NaHCO_3_ (60 mL). The organic phase was separated and extracted with an additional DCM (2 × 100 mL). The combined organic layers were dried over anhydrous Na_2_SO_4_, filtered, and concentrated under reduced pressure. The resulting residue was purified by column chromatography (silica gel, EtOAc/hexanes, 1:9) to obtain TBDPS-ether **5** (5.96 g, 85%) as a white solid: ^1^H NMR (400 MHz, CDCl_3_) δ 7.75–7.65 (m, 4H), 7.46–7.32 (m, 6H), 5.17–5.09 (m, 1H), 3.66–3.42 (m, 1H), 2.59–2.44 (m, 1H), 2.43–2.26 (m, 1H), 2.22–2.08 (m, 5H), 2.07–1.85 (m, 2H), 1.80–1.32 (m, 10H), 1.31–1.15 (m, 1H), 1.14–1.03 (m, 10H), 0.99 (s, 3H), 0.87 (ddd, *J* = 13.9, 6.6, 3.6 Hz, 2H), 0.61 (s, 3H); ^13^C NMR (100 MHz, CDCl_3_) δ 209.5, 141.2, 135.7 (2C), 134.8, 134.7, 129.4 (2C), 127.4 (2C), 120.8, 73.1, 63.7, 56.9, 49.9, 43.9, 42.4, 38.8, 37.2, 36.5, 31.8 (2C), 31.7, 31.5, 27.0, 24.7, 22.8, 21.0, 19.4, 19.1, 13.2.

2-((3*S*,8*S*,9*S*,10*R*,13*S*,14*S*,17*S*)-3-((*tert*-Butyldiphenylsilyl)oxy)-10,13-dimethyl-2,3,4,7,8,9,10,11,12,13,14,15,16,17-tetradecahydro-1*H*-cyclopenta[*a*]phenanthren-17-yl)-2-oxoethyl acetate (**6**): To a stirred solution of compound **5** (1.00 g, 1.80 mmol) in toluene (28 mL), methanol (3.40 mL) containing BF_3_.OEt_2_ (3.37 mL, 27.0 mmol) and lead tetraacetate (0.870 g, 1.98 mmol) were added at room temperature. After being stirred at room temperature for 4 h, the mixture was poured into ice water and extracted with DCM (3 × 30 mL). The combined organic layers were dried over anhydrous Na_2_SO_4_, filtered, and concentrated under reduced pressure to give a residue, which was purified by column chromatography (silica gel, EtOAc/hexanes, 15:85) to obtain compound **6** (0.696 g, 63%) as a white powder: ^1^H NMR (400 MHz, CDCl_3_) δ 7.75–7.61 (m, 4H), 7.50–7.29 (m, 6H), 5.12 (d, *J* = 5.1 Hz, 1H), 4.70 (dd, *J* = 16.9, 6.6 Hz, 1H), 4.51 (dd, *J* = 16.8, 5.9 Hz, 1H), 3.63–3.44 (m, 1H), 2.55–2.25 (m, 2H), 2.24–2.08 (m, 5H), 2.07–1.81 (m, 2H), 1.74–1.52 (m, 6H), 1.50–1.37 (m, 3H), 1.35–1.18 (m, 2H), 1.15–0.92 (m, 13H), 0.85 (ddd, *J* = 22.5, 14.4, 8.3 Hz, 2H), 0.68–0.61 (m, 3H); ^13^C NMR (100 MHz, CDCl_3_) δ 203.8, 170.2, 141.3, 135.7, 135.6, 134.7 (2C), 129.45 (2C), 127.4 (2C), 120.7, 73.1, 69.1, 59.2 (2C), 57.0, 49.8, 44.6, 42.4, 38.5, 37.1, 36.5, 31.8 (2C), 31.7, 24.5, 22.8, 20.9, 20.4, 19.3, 19.1, 13.0.

1-((3*S*,8*S*,9*S*,10*R*,13*S*,14*S*,17*S*)-3-((*tert*-Butyldiphenylsilyl)oxy)-10,13-dimethyl-2,3,4,7,8,9,10,11,12,13,14,15,16,17-tetradecahydro-1*H*-cyclopenta[*a*]phenanthren-17-yl)ethane-1,2-diol (**7**): A 100-mL, two-necked, round-bottomed flask was charged with LiAlH_4_ (0.860 g, 22.7 mmol) under nitrogen and cooled to 0 °C. Anhydrous THF (40 mL) followed by compound **6** (3.48 g, 5.68 mmol) in THF (10 mL) was added dropwise, and the resulting mixture was stirred at ambient temperature for 30 min. The reaction mixture was quenched with H_2_O (0.9 mL), NaOH solution (0.9 mL, 15%), and H_2_O (2.7 mL) and stirred for another 10 min. The precipitate was filtered and washed with Et_2_O. The filtrate was dried over anhydrous Na_2_SO_4_, filtered and concentrated under reduced pressure to give a residue, which was purified by column chromatography (silica gel, EtOAc/hexanes, 3:7) to obtain diol **7** (2.18 g, 67%) as a white foam: ^1^H NMR (400 MHz, CDCl_3_) δ 7.72–7.63 (m, 4H), 7.47–7.31 (m, 6H), 5.12 (d, *J* = 5.0 Hz, 1H), 3.64 (d, *J* = 9.3 Hz, 2H), 3.60–3.46 (m, 1H), 3.36 (t, *J* = 9.2 Hz, 1H), 2.33 (t, *J* = 12.2 Hz, 1H), 2.22–1.98 (m, 2H), 1.99–1.79 (m, 3H), 1.77–1.53 (m, 4H (overlapped with H_2_O), 1.52–1.33 (m, 5H), 1.31–1.10 (m, 3H), 1.05 (s, 9H), 0.99 (s, 3H), 0.92–0.78 (m, 2H), 0.76 (s, 3H); ^13^C NMR (100 MHz, CDCl_3_) δ 141.3, 135.7 (2C), 134.8 (2C), 129.4 (2C), 127.4 (2C), 120.8, 74.6, 73.2, 66.4, 55.9, 52.4, 50.0, 42.4 (2C), 39.7, 37.2, 36.5, 31.8 (2C), 31.7, 26.9, 24.6, 24.5, 20.8, 19.4, 19.1, 12.3.

*tert*-Butyl(((3*S*,8*S*,9*S*,10*R*,13*S*,14*S*,17*R*)-17-ethynyl-10,13-dimethyl-2,3,4,7,8,9,10,11,12,13,14,15,16,17-tetradecahydro-1*H*-cyclopenta[*a*]phenanthren-3-yl)oxy)diphenylsilane (**8**): Sodium periodate (0.560 g, 2.62 mmol) was added to a solution of diol **7** (0.500 g, 0.872 mmol) in THF:H_2_O (8:2, 20 mL) and the solution was stirred for 1 h. The reaction mixture was then diluted with water (20 mL). The organic phase was separated and extracted with EtOAc (3 × 30 mL). The combined organic layers were dried over anhydrous Na_2_SO_4_, filtered, and concentrated under reduced pressure. The resulting residue was purified by column chromatography (silica gel, EtOAc/hexanes, 3:7) to afford the corresponding aldehyde (0.500 g), which was used for the next step without further purification.

To a solution of tetrabromomethane (0.910 g, 2.77 mmol) in anhydrous DCM (20 mL) triphenylphosphine (1.45 g, 5.55 mmol) was added at 0 °C, and the resulting mixture was stirred for 10 min. Subsequently, a solution of the above aldehyde (0.500 g, 0.920 mmol) in DCM (5 mL) was added. After stirring for 20 min, the reaction mixture was diluted with DCM (30 mL). The organic phase was washed with water (30 mL) and saturated NaCl solution (30 mL), dried over anhydrous Na_2_SO_4_, filtered, and concentrated under reduced pressure to give dibromoalkene (0.700 g), which was also used for the next step without further purification.

A solution of n-BuLi (1.6 M) in hexanes (2.51 mL, 4.02 mmol) was added to a solution of dibromoalkene (0.700 g, 1.00 mmol) in anhydrous THF (20 mL) at –78 °C, and the resulting mixture was stirred at the same temperature for 1 h. The reaction mixture was quenched with saturated aqueous NH_4_Cl (20 mL), and the mixture was extracted with EtOAc (3 × 20 mL). The combined organic layers were dried over anhydrous Na_2_SO_4_, filtered, and concentrated under reduced pressure. The resulting residue was purified by column chromatography (silica gel, hexanes/EtOAc, 1:9) to afford the alkyne **8** (0.330 g, 70%) as a white solid: ^1^H NMR (400 MHz, CDCl_3_) δ 7.76–7.61 (m, 4H), 7.51–7.28 (m, 6H), 5.12 (d, *J* = 5.2 Hz, 1H), 3.69–3.42 (m, 1H), 2.35 (dt, *J* = 13.2, 11.3 Hz, 1H), 2.21–1.78 (m, 6H), 1.75–1.57 (m, 5H), 1.53–1.35 (m, 4H), 1.35–1.12 (m, 1H), 1.06 (s, 9H), 1.03 (d, *J* = 4.5 Hz, 1H), 1.00 (s, 3H), 0.86 (m, 3H), 0.79 (s, 3H).

General procedure A for synthesizing silyl or Fmoc or Boc-protected triazole analogs (9, 10, and 18), simplified small molecules **33**, and **39**: A mixture of alkyne (1.0 equiv), azide (1.5 equiv), and DMF (6 mL) was combined with sodium ascorbate (0.40 equiv) in H_2_O (3 mL) and stirred for two min at ambient temperature. Next, CuSO_4_·5H_2_O (0.20 equiv) in H_2_O (3 mL) was added to the mixture. The mixture was stirred at room temperature for 12 h, and then water was added (6 mL) and extracted with EtOAc (3 × 10 mL). The combined organic layers were dried over anhydrous Na_2_SO_4_, filtered, and concentrated under reduced pressure. The resulting residue was purified by column chromatography (silica gel, hexanes/EtOAc, 2:8) to yield the triazole analogs.

1-Benzyl-4-((3*S*,8*S*,9*S*,10*R*,13*S*,14*S*,17*S*)-3-((*tert*-butyldiphenylsilyl)oxy)-10,13-dimethyl-2,3,4,7,8,9,10,11,12,13,14,15,16,17-tetradecahydro-1*H*-cyclopenta[*a*]phenanthren-17-yl)-1*H*-1,2,3-triazole (**9**): Compound **9** was prepared following by general procedure **A**, using alkyne **8** (0.300 g, 0.559 mmol), benzyl azide (0.112 g, 0.838 mmol), CuSO_4_·5H_2_O (27.0 mg, 0.011 mmol), and sodium ascorbate (44.3 mg, 0.022 mmol)) to yield **9** (0.300 g, 80%) as a white foam: ^1^H NMR (400 MHz, CDCl_3_) δ 7.70–7.65 (m, 4H), 7.45–7.32 (m, 9H), 7.23–7.20 (m, 2H), 7.10 (s, 1H), 5.55–5.46 (m, 2H), 5.13 (d, *J* = 5.0 Hz, 1H), 3.55–3.44 (m, 1H), 2.75 (t, *J* = 9.8 Hz, 1H), 2.37–2.26 (m, 1H), 2.16–1.90 (m, 4H), 1.80–1.20 (m, 12H), 1.05 (s, 9H), 0.97 (s, 3H), 0.92–0.81 (m, 2H), 0.44 (s, 3H); ^13^C NMR (100 MHz, CDCl_3_) δ 149.4, 141.3, 135.8 (2C), 135.12, 134.6, 129.5 (2C), 129.0, 128.5, 127.8, 127.5 (2C), 121.0, 120.9, 73.1, 55.9, 53.9, 50.0, 47.8, 43.5, 42.4, 37.5, 37.1, 36.5, 32.2, 31.8 (2C), 27.0, 26.6, 24.5, 20.6, 19.5, 19.1, 13.0. HRMS (ESI) calcd for C_44_H_56_N_3_OSi (M + H)^+^ 670.4193, found 670.4189.

Methyl 2-(4-((3*S*,8*S*,9*S*,10*R*,13*S*,14*S*,17*S*)-3-((*tert*-butyldiphenylsilyl)oxy)-10,13-dimethyl-2,3,4,7,8,9,10,11,12,13,14,15,16,17-tetradecahydro-1*H*-cyclopenta[*a*]phenanthren-17-yl)-1*H*-1,2,3-triazol-1-yl)acetate (**10**): Compound **10** was prepared using general procedure **A**, using alkyne **8** (400 mg, 0.745 mmol), methyl 2-azidoacetate (257 mg, 2.23 mmol), CuSO_4_·5H_2_O (37.2 mg, 0.015 mmol), and sodium ascorbate (59.0 mg, 0.030 mmol) to yield **10** (0.330 g, 68% yield) as a white foam: ^1^H NMR (400 MHz, CDCl_3_) δ 7.70–7.65 (m, 4H), 7.44–7.34 (m, 7H), 5.15–5.11 (m, 3H), 3.79 (s, 3H), 3.60–3.46 (m, 1H), 2.80 (t, *J* = 9.8 Hz, 1H), 2.37–2.30 (m, 1H), 2.17–1.94 (m, 4H), 1.83–1.59 (m, 5H), 1.58–1.44 (m, 3H), 1.40–1.21 (m, 3H), 1.18–1.11 (m, 1H), 1.06 (s, 9H), 0.98 (s, 3H), 0.94–0.82 (m, 2H), 0.49 (s, 3H); ^13^C NMR (100 MHz, CDCl_3_) δ 167.0, 149.3, 141.4, 135.7 (2C), 134.8, 129.4 (2C), 127.4 (2C), 122.2, 120.9, 73.2, 56.0, 52.9, 50.5, 50.1, 47.8, 43.5, 42.4, 37.6, 37.2, 36.6, 32.2, 31.8 (2C), 27.0, 26.6, 24.5, 20.6, 19.4, 19.1, 12.9; HRMS (ESI) calcd for C_40_H_54_N_3_O_3_Si (M + H)^+^ 652.3934, found 652.3919.

General procedure B for synthesizing hydroxymethyl triazole analogs (**11** and **34**): The mixture of HCHO (10 equiv, 37% aq), glacial AcOH (1.5 equiv), and 1,4- dioxane (1 mL) was stirred for 15 min, then NaN_3_ (1.5 equiv), followed by the alkyne (1 equiv), was added to the reaction. After 10 min of stirring, sodium ascorbate (0.2 equiv) and CuSO_4_^.^5H_2_O (0.4 equiv) were added. The mixture was stirred for 18 h at room temperature, then diluted with H_2_O (20 mL), extracted with CHCl_3_ (3 × 20 mL), dried over Na_2_SO_4_, filtered, and concentrated on a rotary evaporator to give a residue. The crude product was purified by column chromatography (acetone: hexanes, 3:7) to give a regio-isomeric mixture of hydroxy methyl triazoles, which was subjected to the next step without further purification.

(4-((3*S*,8*S*,9*S*,10*R*,13*S*,14*S*,17*S*)-3-((*tert*-Butyldiphenylsilyl)oxy)-10,13-dimethyl-2,3,4,7,8,9,10,11,12,13,14,15,16,17-tetradecahydro-1*H*-cyclopenta[*a*]phenanthren-17-yl)-1*H*-1,2,3-triazol-1-yl)methanol (**11a+b**): Compound **11a+b** was prepared following general procedure **B** using alkyne **8** (0.400 g, 0.745 mmol), HCHO (0.600 mL, 7.45 mmol, 37% aq), and glacial AcOH (64.0 μL, 1.19 mol), then NaN_3_ (73.0 mg, 1.5 mmol), sodium ascorbate (59.0 mg, 0.030 mol), and CuSO_4_^.^5H_2_O (37.0 mg, 0.015 mmol). The crude product was purified by flash column chromatography (acetone: hexanes, 3:7) to give a regio-isomeric mixture of hydroxy methyl triazoles **11a** and **11b** as a white foam (0.309 g, 65% yield), which was used for the next step without further purification.

4-((3*S*,8*S*,9*S*,10*R*,13*S*,14*S*,17*S*)-3-((*tert*-Butyldiphenylsilyl)oxy)-10,13-dimethyl-2,3,4,7,8,9,10,11,12,13,14,15,16,17-tetradecahydro-1*H*-cyclopenta[*a*]phenanthren-17-yl)-1*H*-1,2,3-triazole (**12**): The regioisomeric mixture **11a+b** (0.220g, 0.361 mmol) and active MnO_2_ (0.314 g, 3.61 mmol) in CHCl_3_ (20 mL) was stirred under reflux for 20 h. Then, the reaction was filtered through Celite, washed with CHCl_3_:MeOH, 1:1, and the solvent was removed under reduced pressure. The residue was purified by column chromatography to obtain **12** (0.172 g, 82%) as a white foam: ^1^H NMR (400 MHz, CDCl_3_) δ 7.72–7.64 (m, 4H), 7.50 (s, 1H), 7.45–7.32 (m, 6H), 5.14 (d, *J* = 5.2 Hz, 1H), 3.64–3.45 (m, 1H), 2.77 (t, *J* = 9.8 Hz, 1H), 2.33 (td, *J* = 13.4, 6.8 Hz, 1H), 2.23–1.87 (m, 4H), 1.86–1.10 (m, 13H), 1.06 (d, *J* = 5.7 Hz, 9H), 1.01–0.96 (m, 3H), 0.95–0.80 (m, 2H), 0.49 (s, 3H). HRMS (ESI) calcd for C_37_H_50_N_3_OSi (M + H)^+^ 580.3723, found 580.3740.

General procedure C for silyl deprotection: The silylated triazole (0.10 mmol HCl in MeOH (1 N, 5 mL) and the solution were stirred for 5 h at room temperature. The reaction mixture was concentrated in vacuo. The residue was E_3_N (1 mL) to obtain a solid, which was filtered off and purified by column chromatography (silica gel, acetone/hexanes, 4:6) or washing with DCM to give the triazole targets.

(3*S*,8*S*,9*S*,10*R*,13*S*,14*S*,17*S*)-17-(1-Benzyl-1*H*-1,2,3-triazol-4-yl)-10,13-dimethyl-2,3,4,7,8,9,10,11,12,13,14,15,16,17-tetradecahydro-1*H*-cyclopenta[*a*]phenanthren-3-ol (**13**): Compound **13** was prepared following general procedure **C** using silylated benzyl triazole **9** (80 mg, 0.12 mmol) in 1 N HCl in MeOH (5 mL). The solid was washed with DCM to give pure **13** (41 mg, 80%) as a white solid: mp 286–287 °C; ${\left[\alpha \right]}_{D}^{26}$–9.41 (*c* 1.55, CHCl_3_: MeOH (3:1); ^1^H NMR (400 MHz, CDCl_3_+CD_3_OD) δ 7.36–7.26 (m, 4H), 7.25–7.15 (m, 2H), 5.48 (s, 2H), 5.31 (dt, *J* = 4.1, 1.9 Hz, 1H), 3.85 (s, 1H), 3.42 (tt, *J* = 10.7, 4.8 Hz, 1H), 2.73 (t, *J* = 9.8 Hz, 1H), 2.19 (dtdd, *J* = 15.8, 13.2, 5.3, 2.3 Hz, 2H), 2.10–1.87 (m, 3H), 1.84–1.66 (m, 4H), 1.60–0.97 (m, 10H), 0.96 (s, 3H), 0.44 (s, 3H); ^13^C NMR (100 MHz, CDCl_3_+CD_3_OD) δ 153.0, 144.8, 138.7, 132.9, 132.5, 131.6, 125.3, 125.1, 75.0, 59.8, 57.8, 54.1, 51.7, 47.3, 45.6, 41.38, 41.15, 40.4, 36.2, 35.6, 34.9, 30.5, 28.3, 24.5, 23.1, 16.6; HRMS (ESI) calcd for C_28_H_38_N_3_O (M + H)^+^ 432.3015, found 432.3018.

2-(4-((3*S*,8*S*,9*S*,10*R*,13*S*,14*S*,17*S*)-3-Hydroxy-10,13-dimethyl-2,3,4,7,8,9,10,11,12,13,14,15,16,17-tetradecahydro-1*H*-cyclopenta[*a*]phenanthren-17-yl)-1*H*-1,2,3-triazol-1-yl)acetic acid (**14**): Compound **14** was prepared following general procedure **C** using silylated triazole **10** (60 mg, 0.092 mmol) in HCl in MeOH (1 N, 4 mL). The residue was purified by column chromatography (acetone/hexanes, 4:6) to obtain **14** (27 mg, 70%) as a white solid: mp 221–223 ^°^C; ${\left[\alpha \right]}_{D}^{26}$ –15.68 (*c* 0.38 CHCl_3_: MeOH (3:1)); ^1^H NMR (400 MHz, CDCl_3_+CD_3_OD) δ 7.40 (s, 1H), 5.28 (d, *J* = 5.2 Hz, 1H), 5.09 (s, 2H), 3.73 (s, 3H), 3.29 (s, 1H), 2.74 (t, *J* = 9.8 Hz, 1H), 2.17 (p, *J* = 12.8 Hz, 2H), 2.08–1.90 (m, 3H), 1.75 (dd, *J* = 18.0, 11.6 Hz, 4H), 1.58–1.10 (m, 9H complex), 1.07–0.85 (m, 5H), 0.83–0.71 (m, 1H), 0.44 (s, 3H); HRMS (ESI) calcd for C_24_H_36_N_3_O_3_ (M + H)^+^ 414.2757, found 414.2767.

(3*S*,8*S*,9*S*,10*R*,13*S*,14*S*,17*S*)-10,13-Dimethyl-17-(1*H*-1,2,3-triazol-4-yl)-2,3,4,7,8,9,10,11,12,13,14,15,16,17-tetradecahydro-1*H*-cyclopenta[*a*]phenanthren-3-ol (**15**): Compound **15** was prepared following general procedure **C** using silylated triazole **12** (0.07 g, 0.12 mmol in 1 N HCl in MeOH (6 mL). The residue was purified by column chromatography (acetone/hexanes, 4:6) to obtain **15** (0.027 g, 67%) as a white solid: mp. 236–238 °C. Spectroscopic data for compound **15** were consistent with previously reported data [42].

(3*S*,8*S*,9*S*,10*R*,13*S*,14*S*,17*R*)-17-Ethynyl-10,13-dimethyl-2,3,4,7,8,9,10,11,12,13,14,15,16,17-tetradecahydro-1*H*-cyclopenta[*a*]phenanthren-3-ol (**16**): Silylated alkyne **8** (0.48 g, 0.89 mmol) was dissolved in 1 N HCl in MeOH (10 mL) and the solution was stirred for 5 h at room temperature. The reaction mixture was concentrated in vacuo. The residue was purified by column chromatography (silica gel, acetone/hexanes, 2:7) to give compound **16** (0.168 g, 63%) as a white solid. ^1^H NMR (400 MHz, CDCl_3_) δ 5.36–5.29 (m, 1H), 3.59–3.43 (m, 1H), 2.38–2.10 (m, 3H), 2.09–1.94 (m, 3H), 1.93–1.77 (m, 3H), 1.76–1.37 (m, 8H), 1.34–1.14 (m, 1H), 1.16–0.87 (m, 7H), 0.79 (s, 3H); ^13^C NMR (100 MHz, CDCl_3_) δ 140.8, 121.4, 85.9, 71.7, 69.8, 54.9, 50.1, 43.6, 42.2, 41.9, 37.3, 37.1, 36.6, 32.4, 31.8, 31.6, 29.0, 24.6, 20.8, 19.4, 13.3. Spectroscopic data for compound **12** were consistent with previously reported data [43].

(3*S*,8*S*,9*S*,10*R*,13*S*,14*S*,17*R*)-17-Ethynyl-10,13-dimethyl-2,3,4,7,8,9,10,11,12,13,14,15,16,17-tetradecahydro-1*H*-cyclopenta[*a*]phenanthren-3-yl *N*^2^,*N*^6^-bis(*tert*-butoxycarbonyl)-*L*-lysinate (**17**): To a solution of compound **16** (0.19 g, 0.63 mmol), *N,N*-diBoc-L-lysine (0.24 g, 0.70 mmol), and 4-dimethylaminopyridine (7 mg, 0.006 mmol) in anhydrous THF (12 mL) dicyclohexylcarbodiimide (0.10 g, 0.70 mmol) was added at room temperature. The solution was stirred overnight and then filtered through Celite. The filtrate was concentrated under reduced pressure, and the resulting residue was purified by column chromatography (acetone/hexanes, 3:7) to give compound **17** (0.30 g, 76%) as a colorless oil: ^1^H NMR (400 MHz, CDCl_3_) δ 5.37 (d, *J* = 4.5 Hz, 1H), 5.07 (d, *J* = 7.0 Hz, 1H), 4.73–4.51 (m, 2H), 4.22 (d, *J* = 4.8 Hz, 1H), 3.11 (d, *J* = 6.2 Hz, 2H), 2.31 (d, *J* = 7.6 Hz, 2H), 2.25–2.13 (m, 1H), 2.12–1.95 (m, 2H), 1.89–1.34 (m, 34H), 1.27–1.21 (m, 1H), 1.16–0.91 (m, 7H), 0.81 (s, 3H); ^13^C NMR (100 MHz, CDCl_3_) δ 172.1, 156.0, 155.4, 139.4, 122.6, 85.8, 79.7, 79.1, 74.8, 69.8, 54.8, 53.3, 50.0, 43.6, 41.8, 40.1, 37.9, 37.0, 36.9, 36.6, 32.5, 32.3, 31.8, 29.6, 29.0, 28.4, 28.3, 27.6, 24.6, 22.4, 20.8, 19.3, 13.3.

(3*S*,10*R*,13*S*,17*S*)-17-(1-Benzyl-1*H*-1,2,3-triazol-4-yl)-10,13-dimethyl-2,3,4,7,8,9,10,11,12,13,14,15,16,17-tetradecahydro-1*H*-cyclopenta[*a*]phenanthren-3-yl *N*^2^,*N*^6^-bis(*tert*-butoxycarbonyl)-*L*-lysinate (**18**): Compound **18** was prepared following general procedure **A** using alkyne **17** (0.31 g, 0.50 mmol), benzyl azide (46 mg, 0.18 mmol), sodium ascorbate (39 mg, 0.20 mmol), and CuSO_4_^.^5H_2_O (24 mg, 0.10 mmol) to yield **18** (0.29 g, 78%) as a solid: ^1^H NMR (400 MHz, CDCl_3_) δ 7.49–7.39 (m, 3H), 7.34 (d, *J* = 5.8 Hz, 1H), 7.32 (dd, *J* = 7.5, 1.8 Hz, 2H), 5.65–5.55 (m, 2H), 5.47 (d, *J* = 3.9 Hz, 1H), 5.18 (d, *J* = 7.5 Hz, 1H), 4.85–4.59 (m, 2H), 4.31 (d, *J* = 4.6 Hz, 1H), 3.19 (d, *J* = 6.1 Hz, 2H), 2.87 (t, *J* = 9.8 Hz, 1H), 2.40 (d, *J* = 7.7 Hz, 2H), 2.23–2.02 (m, 3H), 1.99–1.56 (m, 14H), 1.55–1.50 (m, 18H), 1.47–1.23 (m, 6H), 1.16–1.00 (m, 4H), 0.57 (s, 3H); ^13^C NMR (100 MHz, CDCl_3_) δ 172.1, 156.0, 155.4, 149.2, 139.4, 135.1, 129.0, 128.5, 127.7, 122.7, 120.8, 79.7, 79.1, 74.9, 55.9, 53.9, 53.3, 50.1, 47.8, 43.5, 40.1, 37.9, 37.6, 36.9, 36.6, 32.4, 32.2, 31.8, 29.5, 28.4, 28.3, 27.6, 26.6, 24.5, 22.4, 20.6, 19.3, 12.9.

(3*S*,10*R*,13*S*,17*S*)-17-(1-Benzyl-1*H*-1,2,3-triazol-4-yl)-10,13-dimethyl-2,3,4,7,8,9,10,11,12,13,14,15,16,17-tetradecahydro-1*H*-cyclopenta[*a*]phenanthren-3-yl *L*-Lysinate Dihydrochloride (**19**): Compound **18** (0.20 g, 0.26 mmol) was dissolved in 2 N HCl in ether (10 mL), and the reaction mixture was stirred at room temperature overnight. The solvent was removed under reduced pressure, and the resulting residue was purified by column chromatography (silica gel, MeOH/DCM, 2:8) to obtain **19** (0.10 g, 71%) as a yellowish solid: mp 258–260 °C; ${\left[\alpha \right]}_{D}^{26}$ − 0.50 (*c* 0.59, MeOH). ^1^H NMR (400 MHz, CD_3_OD) δ 8.02 (s, 1H), 7.36 (s, 5H), 5.56 (d, *J* = 52.2 Hz, 2H), 5.45 (s, 1H), 4.75–4.70 (m, 1H), 4.02 (s, 1H), 2.91 (m, 3H), 2.31 (m, 2H), 2.30–0.84 (m, 26H), 0.53 (s, 3H); LCMS (ESI) *m*/*z* 560.52 (M + H)^+^.

(3*S*,8*R*,9*S*,10*R*,13*S*,14*S*)-3-((*tert*-Butyldiphenylsilyl)oxy)-10,13-Dimethyl-1,2,3,4,7,8,9,10,11,12,13,14,15,16-tetradecahydro-17*H*-cyclopenta[*a*]phenanthren-17-one (**20**): To a solution of dehydro-epi-androsterone (3.00 g, 10.41 mmol) in CH_2_Cl_2_ (75 mL) at 0 °C, imidazole (1.41 g, 20.83 mmol) and TBDPSCl (2.92 mL, 11.45 mmol) were added. After being stirred at ambient temperature for 5 h, the reaction mixture was quenched with saturated aqueous NaHCO_3_ (50 mL) and extracted with CH_2_Cl_2_ (2 × 75 mL). The combined organic layers were dried over Na_2_SO_4_ and concentrated in vacuo to give a residue, which was purified by column chromatography (silica gel, EtOAc/hexanes, 1:9) to give compound **20** (5.37 g, 98%) as a white solid. Spectroscopic data for compound **20** were consistent with previously reported data [44].

(3*S*,8*R*,9*S*,10*R*,13*S*,14*S*,17*S*)-3-((*tert*-Butyldiphenylsilyl)oxy)-10,13-dimethyl-2,3,4,7,8,9,10,11,12,13,14,15,16,17-tetradecahydro-1*H*-cyclopenta[*a*]phenanthren-17-ol (**21**): To a solution of ketone **20** (1.70 g, 3.23 mmol) in MeOH:THF (30 mL, 1:1), NaBH_4_ (0.36 g, 9.69 mmol) was added at 0 °C. After stirring for 1 h, the reaction mixture was quenched by the addition of saturated aqueous NH_4_Cl (50 mL). The mixture was extracted with EtOAc (3 × 50 mL), washed with brine (50 mL), and dried over Na_2_SO_4_. The volatiles were evaporated and purified by flash chromatography (silica gel, EtOAc/hexane 2:8) to afford the alcohol **21** (1.50 g, 88%) as a white solid. Spectroscopic data for compound **21** were consistent with previously reported data [44].

General procedure D for the Mitsunobu azidation: To a solution of triphenylphosphine (1.3 equiv) in THF (30 mL) diethyl azodicarboxylate (1 equiv, 40% solution in toluene) was added at 0 °C, and the resulting orange solution was stirred for 10 min. The alcohol (1.0 equiv) in THF (10 mL) was added to the above solution. After stirring for 10 min, diphenylphosphoryl azide (1.7 equiv) was added. The reaction mixture was allowed to warm to room temperature and stirred for 10 h. The solvent was evaporated, and the resulting residue was purified by column chromatography (silica gel, EtOAc/hexanes, 1:9) to yield the azides.

(((3*S*,8*R*,9*S*,10*R*,13*S*,14*S*,17*R*)-17-Azido-10,13-dimethyl-2,3,4,7,8,9,10,11,12,13,14,15,16,17-tetradecahydro-1*H*-cyclopenta[*a*]phenanthren-3-yl)oxy)(*tert*-butyl)diphenylsilane (**22**): Compound **22** was prepared following general procedure **D** using triphenylphosphine (1.09 g, 4.18 mmol), diethyl azodicarboxylate (2.20 mL, 4.82 mmol, 40% solution in toluene), alcohol **21** (1.70 g, 3.21 mmol), and diphenyl phosphoryl azide (1.18 g, 4.82 mmol) to obtain **22** (1.26 g, 71%) as a viscous oil. ^1^H NMR (400 MHz, CDCl_3_) δ 7.77–7.59 (m, 4H), 7.48–7.29 (m, 6H), 5.18–5.05 (m, 1H), 3.53 (ddd, *J* = 16.4, 10.5, 5.4 Hz, 2H), 2.44–2.23 (m, 1H), 2.24–2.04 (m, 2H), 2.03–1.84 (m, 1H), 1.81–1.29 (m, 11H (overlapped with H_2_O), 1.28–1.12 (m, 2H), 1.06 (s, 9H), 0.98 (s, 3H), 0.92–0.81 (m, 2H), 0.73 (s, 3H); ^13^C NMR (100 MHz, CDCl_3_) δ 141.2, 135.7 (2C), 134.7, 129.4 (2C), 127.4 (2C), 120.8, 73.1, 71.4, 49.8, 49.6, 45.6, 42.4, 37.2, 36.5, 32.4, 32.1, 32.0, 31.8, 28.6, 27.0, 24.7, 20.5, 19.4, 19.1, 17.4.

1-((3*S*,8*R*,9*S*,10*R*,13*S*,14*S*,17*R*)-3-((*tert*-Butyldiphenylsilyl)oxy)-10,13-dimethyl-2,3,4,7,8,9,10,11,12,13,14,15,16,17-tetradecahydro-1*H*-cyclopenta[*a*]phenanthren-17-yl)-4-phenyl-1*H*-1,2,3-triazole (**23**): Compound **23** was prepared following general procedure **A** using azide **22** (0.900 g, 1.62 mmol) and phenylacetylene (0.330 g, 3.25 mmol), sodium ascorbate (128 mg, 0.64 mmol), and CuSO_4_.5H_2_O (80 mg, 0.32 mmol) to yield **23** (0.71 g, 67%) as a solid: ^1^H NMR (400 MHz, CDCl_3_) δ 7.82 (dd, *J* = 8.2, 1.2 Hz, 2H), 7.69–7.60 (m, 5H), 7.47–7.28 (m, 9H), 5.21–5.04 (m, 1H), 4.62 (dt, *J* = 14.0, 7.0 Hz, 1H), 3.61–3.35 (m, 1H), 2.66–2.41 (m, 1H), 2.26 (tt, *J* = 22.9, 11.6 Hz, 2H), 2.19–1.91 (m, 3H), 1.74–1.31 (m, 10H(overlapped with H_2_O)), 1.12–0.99 (m, 9H), 0.97 (s, 3H), 0.95 (s, 3H), 0.83–0.62 (m, 2H), 0.28 (dd, *J* = 18.3, 10.3 Hz, 1H); ^13^C NMR (100 MHz, CDCl_3_) δ 146.9, 141.2, 135.7 (2C), 134.7 (2C), 129.4 (2C), 128.7, 127.9, 127.4 (2C), 125.6, 120.6, 119.5, 73.1, 70.3, 50.2, 49.3, 46.1, 42.4, 37.0, 36.4, 32.4, 32.1, 31.9, 31.7, 28.6, 26.9, 25.3, 20.3, 19.3, 19.1, 18.3; HRMS (ESI) calcd for C_43_H_54_N_3_OSi (M + H)^+^ 656.4036, found 656.4038.

(3*S*,8*R*,9*S*,10*R*,13*S*,14*S*,17*R*)-10,13-Dimethyl-17-(4-phenyl-1*H*-1,2,3-triazol-1-yl)-2,3,4,7,8,9,10,11,12,13,14,15,16,17-tetradecahydro-1*H*-cyclopenta[*a*]phenanthren-3-ol (**24**): Compound **24** was prepared following general procedure **C** using **23** (0.50 g, 0.76 mmol) dissolved in 1 N HCl in MeOH (15 mL). The residue was purified by column chromatography (silica gel, acetone/hexanes, 4:6) to give **24** (0.23 g, 72%) as a white solid: mp 234–236 °C; ${\left[\alpha \right]}_{D}^{26}\;$– 25.36 (*c* 0.40, CHCl_3_); ^1^H NMR (400 MHz, CDCl_3_) δ 7.84 (d, *J* = 7.2 Hz, 2H), 7.67 (s, 1H), 7.42 (t, *J* = 7.6 Hz, 2H), 7.32 (t, *J* = 7.4 Hz, 1H), 5.43–5.28 (m, 1H), 4.64 (dd, *J* = 8.5, 1.6 Hz, 1H), 3.60–3.37 (m, 1H), 2.55 (ddd, *J* = 18.7, 10.8, 2.5 Hz, 1H), 2.43–1.97 (m, 5H), 1.87–1.33 (m, 11H (overlapped with H_2_O)), 1.08–0.77 (m, 8H), 0.46–0.22 (m, 1H); ^13^C NMR (100 MHz, CDCl_3_) δ 146.9, 140.7, 130.7, 128.8, 128.0, 125.6, 121.2, 119.7, 71.6, 70.3, 50.2, 49.4, 46.1, 42.1, 37.1, 36.4, 32.4, 32.2, 31.9, 31.5, 28.6, 25.3, 20.4, 19.3, 18.4; HRMS (ESI) calcd for C_27_H_36_N_3_O (M + H)^+^ 418.2858, found 418.2842.

1-((3*R*,8*R*,9*S*,10*R*,13*S*,14*S*,17*R*)-3-Azido-10,13-dimethyl-2,3,4,7,8,9,10,11,12,13,14,15,16,17-tetradecahydro-1*H*-cyclopenta[*a*]phenanthren-17-yl)-4-phenyl-1*H*-1,2,3-triazole (**25**): Compound **25** was prepared following general procedure **D** using triphenylphosphine (0.16 g, 0.62 mmol), diethyl azodicarboxylate (0.12 g, 0.71 mmol, 40% solution in toluene), alcohol **24** (0.20 g, 0.47 mmol), and diphenyl phosphoryl azide (0.22 mL, 0.81 mmol) to obtain **25** (0.12 g, 58%) as a yellow foam. ^1^H NMR (400 MHz, CDCl_3_) δ 7.89–7.79 (m, 2H), 7.67 (d, *J* = 5.7 Hz, 1H), 7.42 (t, *J* = 7.6 Hz, 2H), 7.32 (t, *J* = 7.4 Hz, 1H), 5.39 (dd, *J* = 13.5, 11.0 Hz, 1H), 4.69 (dd, *J* = 8.6, 1.8 Hz, 1H), 3.88–3.80 (m, 1H), 2.72–2.39 (m, 2H), 2.41–1.97 (m, 4H), 1.90–1.21 (m, 12H), 1.08–0.90 (m, 6H), 0.45–0.21 (m, 1H); ^13^C NMR (100 MHz, CDCl_3_) δ 147.0, 138.0, 128.7, 128.0, 125.6 (2C), 122.6, 119.3, 70.2, 57.9, 50.3, 49.2, 46.0, 37.0, 35.9, 33.4, 32.4, 32.0, 31.9, 28.7, 26.0, 25.2, 20.0, 18.9, 18.3; HRMS (ESI) calcd for C_27_H_35_N_6_ (M + H)^+^ 443.2923, found 443.2911.

4-((3*R*,8*R*,9*S*,10*R*,13*S*,14*S*,17*R*)-10,13-Dimethyl-17-(4-phenyl-1*H*-1,2,3-triazol-1-yl)-2,3,4,7,8,9,10,11,12,13,14,15,16,17-tetradecahydro-1*H*-cyclopenta[*a*]phenanthren-3-yl)morpholine (**26**): To a solution of azide **25** (0.13 g, 0.30 mmol) in THF, triphenyl phosphine (0.10 g, 0.39 mmol) was added at ambient temperature. After stirring for 1 h, the reaction mixture was diluted with water and stirred at room temperature for 10 h. The solvent was evaporated under reduced pressure to give the corresponding amine, which was used for the next step without purification.

To a solution of the crude amine (0.13 g, 0.32 mmol) in toluene (10 mL), 2-bromomethyl ether (0.11 g, 0.48 mmol) and K_2_CO_3_ (0.050 g, 0.64 mmol) were added. The reaction mixture was refluxed for 24 h. After cooling to room temperature, the reaction mixture was filtered and concentrated under reduced pressure. The residue was purified by column chromatography (silica gel, CHCl_3_/MeOH, 1:9) to afford **26** (0.11 g, 77%) as a white solid: mp 178–180 °C; ${\left[\alpha \right]}_{D}^{26}$–15.56 (*c* 0.25, CHCl_3_); ^1^H NMR (400 MHz, CDCl_3_) δ 7.94–7.78 (m, 2H), 7.68 (s, 1H), 7.43 (dd, *J* = 10.4, 4.7 Hz, 2H), 7.32 (dd, *J* = 16.2, 8.7 Hz, 1H), 5.18 (s, 1H), 4.71–4.59 (m, 1H), 3.65 (s, 4H), 2.66–1.98 (m, 10H), 1.86–1.19 (m, 12H (overlapped with H_2_O), 0.99 (d, *J* = 9.7 Hz, 7H), 0.35 (dd, *J* = 12.3, 8.0 Hz, 1H); ^13^C NMR (100 MHz, CDCl_3_) δ 146.9, 140.6, 130.8, 128.8, 128.0, 125.6, 119.9, 119.6, 70.3, 67.1, 59.9, 50.3 (2C), 49.0, 46.1, 36.9, 34.5, 33.3, 32.5, 32.1, 31.8, 28.7, 25.2, 24.2, 20.0, 19.9, 18.4; HRMS (ESI) calcd for C_31_H_43_N_4_O (M + H)^+^ 487.3437, found 487.3437.

(3*S*,8*R*,9*S*,10*R*,13*S*,14*S*,17*R*)-3-((*tert*-Butyldiphenylsilyl)oxy)-10,13-dimethyl-2,3,4,7,8,9,10,11,12,13,14,15,16,17-tetradecahydro-1*H*-cyclopenta[*a*]phenanthren-17-yl 4-nitrobenzoate (**27**): To a solution of triphenylphosphine (3.87 g, 14.77 mmol) in THF (100 mL), diethyl azodicarboxylate (2.87 g, 16.25 mmol) was added at 0 °C. The resulting orange solution was stirred for 10 min. Alcohol **21** (3.90 g, 7.38 mmol) in THF (20 mL) was added to the above solution. After stirring for 10 min, a solution of 4-nitrobenzoic acid (2.96 g, 17.72 mmol) in THF (15 mL) was added. The reaction mixture was allowed to warm to room temperature and stirred for 12 h. The solvent was evaporated under reduced pressure, and the residue was purified by column chromatography (silica gel, EtOAc/hexanes, 15:85) to give impure **30** (3.90 g) as a yellowish foam, which was used directly in the next step.

(3*S*,8*R*,9*S*,10*R*,13*S*,14*S*,17*R*)-3-((*tert*-Butyldiphenylsilyl)oxy)-10,13-dimethyl-2,3,4,7,8,9,10,11,12,13,14,15,16,17-tetradecahydro-1*H*-cyclopenta[*a*]phenanthren-17-ol (**28**): 4-Nitrobenzoate **27** (3.10 g, 4.64 mmol) was dissolved in THF (30 mL) and MeOH (10 mL). Powdered K_2_CO_3_ (1.28 g, 9.29 mmol) was added, and the reaction mixture was stirred at room temperature overnight. EtOAc (100 mL) was added to dilute the reaction and filtered through a pad of Celite. The filtrate was concentrated under reduced pressure, and the resulting residue was purified by column chromatography (EtOAc/hexanes 1:2) to obtain corresponding inverted alcohol **28** (2.07 g, 53% for two steps) as a viscous oil: ^1^H NMR (400 MHz, CDCl_3_) δ 7.79–7.61 (m, 4H), 7.51–7.29 (m, 6H), 5.26–5.07 (m, 1H), 3.73 (d, *J* = 5.9 Hz, 1H), 3.67–3.46 (m, 1H), 2.51–2.25 (m, 1H), 2.23–2.09 (m, 2H), 2.03–1.90 (m, 1H), 1.81–1.28 (m, 13H), 1.22–1.07 (m, 10H), 1.01 (s, 3H), 0.94–0.83 (m, 2H), 0.66 (s, 3H).

1-((3*S*,8*R*,9*S*,10*R*,13*S*,14*S*,17*S*)-3-((*tert*-Butyldiphenylsilyl)oxy)-10,13-dimethyl-2,3,4,7,8,9,10,11,12,13,14,15,16,17-tetradecahydro-1*H*-cyclopenta[*a*]phenanthren-17-yl)-4-phenyl-1*H*-1,2,3-triazole (**29**): To a solution of triphenylphosphine (1.22 g, 4.67 mmol) in THF (50 mL), diethyl azodicarboxylate (0.93 g, 5.39 mmol) was added at 0 °C, and the resulting orange solution was stirred for 10 min. Alcohol (1.90 g, 3.59 mmol) in THF (10 mL) was added to the above solution. After being stirred for 10 min, a solution of diphenylphosphoryl azide (1.68 g, 6.11 mmol) was added. The reaction mixture was allowed to warm to room temperature and stirred for 12 h. The solvent was evaporated under reduced pressure, and the residue was purified by column chromatography (silica gel, EtOAc/hexanes) to give azide (0.85 g, 43%) as a yellow oil. LCMS (ESI) *m*/*z* 576.24 (M + Na)^+^.

To a mixture of the azide (0.40 g, 0.72 mmol) and phenylacetylene (0.14 g, 1.44 mmol) in DMF (6 mL), sodium ascorbate (57 mg, 0.29 mmol) in H_2_O (3 mL) was added and stirred for two min at ambient temperature. Then, a CuSO_4_·5H_2_O (35 mg, 0.14 mmol) in H_2_O (3 mL) was added to the above mixture. The mixture was stirred at room temperature for 12 h and extracted with EtOAc (3 × 20 mL). The evaporation of combined organic extracts under reduced pressure afforded a green solid, which was purified by flash column chromatography (silica gel, acetone/hexanes, 2:8) to give silyl protected triazole **29** (0.27 g, 58%) as a viscous oil: ^1^H NMR (400 MHz, CDCl_3_) δ 7.86–7.80 (m, 2H), 7.73 (s, 1H), 7.68 (ddd, *J* = 7.9, 3.4, 1.5 Hz, 4H), 7.47–7.31 (m, 9H), 5.14 (d, *J* = 5.1 Hz, 1H), 4.41 (t, *J* = 9.5 Hz, 1H), 3.63–3.45 (m, 1H), 2.69–2.46 (m, 1H), 2.41–2.22 (m, 2H), 2.15 (dd, *J* = 13.3, 2.8 Hz, 1H), 2.09–1.76 (m, 3H), 1.77–1.43 (m, 8H overlapped with H_2_O), 1.40–1.12 (m, 4H), 1.06 (s, 9H), 1.00 (d, *J* = 11.1 Hz, 3H), 0.97–0.80 (m, 2H), 0.59 (s, 3H).

(3*S*,8*R*,9*S*,10*R*,13*S*,14*S*,17*S*)-10,13-Dimethyl-17-(4-phenyl-1*H*-1,2,3-triazol-1-yl)-2,3,4,7,8,9,10,11,12,13,14,15,16,17-tetradecahydro-1*H*-cyclopenta[*a*]phenanthren-3-ol (**30**): Compound **30** was prepared following general procedure **C** using silylated triazole (0.17 g, 0.25 mmol) in 1 N HCl in MeOH (10 mL) and the solution was stirred for 5 h at room temperature. The reaction mixture was concentrated under reduced pressure. The residue was purified by column chromatography (silica gel, acetone/hexanes, 3:7) to give compound **30** (70 mg, 65%) as a white solid: mp 278–280 °C. ^1^H NMR (400 MHz, CDCl_3_) δ 7.85 (s, 1H), 7.77 (d, *J* = 6.8 Hz, 2H), 7.38 (t, *J* = 7.1 Hz, 2H), 7.30 (t, *J* = 7.9 Hz, 1H), 5.28 (d, *J* = 23.0 Hz, 1H), 4.42 (t, *J* = 8.9 Hz, 1H), 3.40 (m, 1H), 2.53 (m, 1H), 2.22 (dt, *J* = 23.8, 15.3 Hz, 3H), 2.01 (d, *J* = 15.0 Hz, 1H), 1.79 (d, *J* = 13.0 Hz, 4H), 1.69–1.14 (m, 8H), 1.13–0.87 (m, 5H), 0.56 (s, 3H); ^13^C NMR (100 MHz, CDCl_3_) δ 150.5, 144.9, 133.4, 132.8, 132.4, 129.6, 124.7, 124.0, 75.0, 74.9, 57.0, 53.9, 48.2, 45.6, 41.1, 40.6, 40.4, 35.9, 35.3, 34.9, 29.6, 27.3, 24.4, 23.1, 15.8. LCMS (ESI) *m*/*z* 418.41 (M + H)^+^.

(1*R*,7a*R*)-1-Ethynyl-7a-methyl-1,2,3,6,7,7a-hexahydro-5*H*-inden-5-one (**32**): To a solution of Bestmann reagent (0.13 g, 0.71 mmol) in MeOH (10 mL), K_2_CO_3_ (0.19 g, 1.43 mmol) was added and the mixture was stirred for 10 minutes at 0 °C. Then, a solution of aldehyde **31** (85 mg, 0.47 mmol) in MeOH (5 mL) was added. After stirring at room temperature for 8 h, the reaction mixture was treated with saturated aqueous NaHCO_3,_ and then the methanol was removed under reduced pressure. The residue was dissolved in EtOAc (30 mL) and washed with water, dried over Na_2_SO_4,_ and concentrated under reduced pressure to give **32** (54 mg, 65%) as a yellow solid. ^1^H NMR (400 MHz, CDCl_3_) δ 5.77 (s, 1H), 2.72 (dddd, *J* = 9.0, 7.2, 5.0, 2.7 Hz, 1H), 2.59–2.33 (m, 4H), 2.23–2.11 (m, 3H), 2.04–1.89 (m, 1H), 1.78 (td, *J* = 13.7, 5.2 Hz, 1H), 1.23 (d, *J* = 12.4 Hz, 3H); ^13^C NMR (100 MHz, CDCl_3_) δ 198.8, 175.3, 122.5, 82.6, 71.7, 45.3, 42.3, 34.5, 33.3, 28.7, 28.2, 17.6; HRMS (ESI) calcd for C_12_H_15_O (M + H)^+^ 175.1123, found 175.1128.

(1*S*,7a*R*)-1-(1-Benzyl-1*H*-1,2,3-triazol-4-yl)-7a-methyl-1,2,3,6,7,7a-hexahydro-5*H*-inden-5-one (**33**): Compound **33** was prepared following general procedure **A** using benzyl azide (57 mg, 0.43 mmol) and alkyne **32** (50 mg, 0.28 mmol), sodium ascorbate (22 mg, 0.11 mmol), and CuSO_4_.5H_2_O (13 mg, 0.056 mmol) to yield **33** as a white solid (59 mg, 67%). mp 122–125 °C; ${\left[\alpha \right]}_{D}^{26}$ + 71.2 (*c* 0.40, CHCl_3_); ^1^H NMR (400 MHz, CDCl_3_) δ 7.41–7.24 (m, 3H), 7.25–7.13 (m, 3H), 5.76 (d, *J* = 10.1 Hz, 1H), 5.53–5.37 (m, 2H), 2.96 (dd, *J* = 12.3, 7.4 Hz, 1H), 2.82–2.65 (m, 1H), 2.59–2.00 (m, 6H), 1.96–1.86 (m, 1H), 0.82 (s, 3H); HRMS (ESI) calcd for C_19_H_22_N_3_O (M + H)^+^ 308.1763, found 308.1766.

(1*S*,7a*R*)-1-(2-(Hydroxymethyl)-2*H*-1,2,3-triazol-4-yl)-7a-methyl-1,2,3,6,7,7a-hexahydro-5*H*-inden-5-one (**34a+34b**): To a mixture of HCHO (0.41 mL, 5.17 mmol, 10 equiv, 37% aq.), glacial AcOH (0.04 mL, 0.77 mmol), and 1,4- dioxane (0.41 mL), stirred for 15 min, NaN_3_ (0.05 g, 0.77 mmol) was added, followed by alkyne **32** (0.09 g, 0.51 mmol). After an additional 10 min of stirring, sodium ascorbate (38.8 mg, 0.196 mol, 20 mol %) was added, followed by CuSO_4_^.^5H_2_O (39.6 mg, 0.20 mmol). The mixture was stirred for 18 h at room temperature and extracted with CHCl_3_ (3 × 10 mL). Combined organic layers were dried over Na_2_SO_4_, filtered, and concentrated under reduced pressure to obtain the residue, which was a regioisomeric mixture of **34a** and **34b** (7:3) (0.07 g, 54%). The crude product was sufficiently pure to be used without further purification: ^1^H NMR (400 MHz, CD_3_OD) δ 7.44 (s, 0.7H), 7.19 (s, 0.3H), 5.88–5.56 (m, 2H), 4.97–4.55 (m, 2H), 2.94 (dd, *J* = 12.3, 7.4 Hz, 1H), 2.83–2.63 (m, 1H), 2.65–1.66 (m, 7H), 1.17 (s, 1H), 0.83 (s, 2H).

(1*S*,7a*R*)-7a-Methyl-1-(1*H*-1,2,3-triazol-4-yl)-1,2,3,6,7,7a-hexahydro-5*H*-inden-one (**35**): The mixture of **34a** and **34b** (65 mg, 0.26 mmol) and active MnO_2_ (220 mg, 2.63 mmol) in CHCl_3_ (10 mL) was stirred under reflux for 20 h. The reaction was filtered through Celite and washed with CHCl_3_: MeOH (1:1, 20 mL). The solvents were removed under reduced pressure. The residue was purified by column chromatography (silica gel, acetone/hexanes, 3:7) to obtain **35** (34 mg, 60%) as a white solid: mp 126–129 °C; ${\left[\alpha \right]}_{D}^{26}$ + 2.25 (*c* 0.31, CHCl_3_); ^1^H NMR (400 MHz, CDCl_3_) δ 7.58 (s, 1H), 5.89 (t, *J* = 1.9 Hz, 1H), 3.09 (dd, *J* = 12.3, 7.4 Hz, 1H), 2.86 (ddt, *J* = 19.8, 10.7, 2.3 Hz, 1H), 2.65 (dtd, *J* = 19.8, 9.0, 1.8 Hz, 1H), 2.57–2.31 (m, 3H), 2.25 (dddd, *J* = 13.1, 9.4, 7.4, 2.3 Hz, 1H), 2.13–1.97 (m, 2H), 0.92 (s, 3H); ^13^C NMR (100 MHz, CDCl_3_) δ 199.8, 177.7, 144.4, 131.9, 122.5, 47.5, 45.7, 35.2, 33.4, 29.0, 26.0, 17.4; HRMS (ESI) calcd for C_12_H_16_N_3_O (M + H)^+^ 218.1293, found 218.1279.

8a-Methyl-6-oxo-1,2,3,4,6,7,8,8a-octahydronaphthalene-1-carbaldehyde (**37**): To a solution of dithiolane **36** (0.40 g, 1.49 mmol) in CH_3_CN: H_2_O (10:1, 20 mL) at room temperature, bis(trifuoroacetoxy)iodobenzene (0.96 g, 2.23 mmol) was added in one portion. After stirring for 10 min, a mixture of water, saturated aqueous NaHCO_3_, and saturated aqueous Na_2_S_2_O_3_ (1:1:1, 18 mL) was added. The aqueous phase was extracted with EtOAc (3 × 20 mL). The combined organics were dried over anhydrous Na_2_SO_4_, filtered, and evaporated under reduced pressure. The resulting residue was purified by column chromatography (silica gel, EtOAc/hexanes, 3:7) to obtain **37** (0.26 g, 92%) as a yellow oil: ^1^H NMR (400 MHz, CDCl_3_) δ 9.83 (dd, *J* = 6.4, 3.0 Hz, 1H), 6.01–5.55 (m, 1H), 2.60–2.20 (m, 6H), 2.19–1.96 (m, 2H), 1.96–1.73 (m, 2H), 1.54–1.36 (m, 1H), 1.32 (s, 3H); ^13^C NMR (100 MHz, CDCl_3_) δ 203.1, 198.6, 167.6, 125.0, 60.0, 38.5, 36.1, 33.6, 32.6, 25.2, 22.2, 18.3.

5-Ethynyl-4a-methyl-4,4a,5,6,7,8-hexahydronaphthalen-2(3*H*)-one (**38**): Compound **38** was synthesized using the same procedure employed for **32** using Bestmann reagent (0.60 g, 3.12 mmol), K_2_CO_3_ (0.860 g, 6.25 mmol), and **37** (0.400 g, 2.08 mmol) to obtain a crude residue, which was purified by column chromatography (silica gel, EtOAc/hexanes, 2:8) to obtain **38** (0.266 g, 68%) as a solid: ^1^H NMR (400 MHz, CDCl_3_) δ 5.79–5.73 (m, 1H), 2.57–2.18 (m, 6H), 1.96–1.74 (m, 4H), 1.43 (ddd, *J* = 17.2, 8.6, 2.9 Hz, 1H), 1.31 (s, 3H), 1.25 (t, *J* = 7.1 Hz, 1H).

5-(1-Benzyl-1*H*-1,2,3-triazol-4-yl)-4a-methyl-4,4a,5,6,7,8-hexahydronaphthalen-2(3*H*)-one (**39**): Compound **39** was prepared following general procedure **A** using benzyl azide (0.21 g, 1.59 mmol) and alkyne **38** (0.20 g, 1.06 mmol), sodium ascorbate (84 mg, 0.42 mmol), and CuSO_4_^.^5H_2_O (52 mg, 0.21 mmol) to yield **39** as a white solid, (0.17 g, 51%). ^1^H NMR (400 MHz, CDCl_3_) δ 7.49–7.37 (m, 3H), 7.35–7.26 (m, 4H), 5.85 (d, *J* = 1.6 Hz, 1H), 5.64–5.48 (m, 2H), 2.89 (dd, *J* = 13.1, 3.4 Hz, 1H), 2.60–2.46 (m, 1H), 2.45–2.31 (m, 3H), 2.16 (qd, *J* = 13.4, 3.7 Hz, 1H), 2.09–1.96 (m, 2H), 1.85 (d, *J* = 13.6 Hz, 1H), 1.76 (dt, *J* = 13.6, 4.3 Hz, 1H), 1.56 (ddd, *J* = 16.2, 10.2, 5.5 Hz, 1H), 1.15 (s, 3H); ^13^C NMR (100 MHz, CDCl_3_) δ 199.5, 169.3, 148.2, 134.8, 129.1, 128.7, 127.8, 124.8, 121.5, 54.0, 46.3, 39.8, 35.9, 33.7, 32.7, 27.6, 26.3, 17.8; HRMS (ESI) calcd for C_20_H_24_N_3_O (M + H)^+^ 322.1919, found 322.1910.

*N*’-(5-(1-Benzyl-1*H*-1,2,3-triazol-4-yl)-4a-methyl-4,4a,5,6,7,8-hexahydronaphthalen-2(3*H*)-ylidene)amidinohydrazone (**40**): To a solution of **39** (116 mg, 0.36 mmol) in EtOH (10 mL), aminoguanidine hydrochloride (39 mg, 0.36 mmol, 1.0 equiv) and HCl (2 N, 1.0 equiv) in EtOH (0.18 mL) were added. The mixture was heated to reflux for 45 min, then cooled to room temperature, concentrated under reduced pressure, and crystallized to obtain the corresponding amidinohydrazone **40** (97 mg, 71%) as a yellow foam: ^1^H NMR (400 MHz, CD_3_OD) δ 7.99 (d, *J* = 3.7 Hz, 1H), 7.58–7.08 (m, 5H), 6.35 (d, *J* = 1.8 Hz, 0.3H), 6.13–5.91 (m, 0.7H), 5.62 (d, *J* = 2.3 Hz, 2H), 2.81 (dd, *J* = 13.1, 3.3 Hz, 1H), 2.72–2.07 (m, 4H), 1.77 (tt, *J* = 13.4, 7.4 Hz, 2H), 1.64–1.36 (m, 2H), 1.09 (s, 1H), 1.03 (s, 2H); HRMS (ESI) calcd for C_21_H_28_N_7_ (M + H)^+^ 378.2406, found 378.2396.

(*E+Z*)-2-(((1*S*,7a*R*,*E*)-5-(2-Carbamimidoylhydrazineylidene)-7a-methyl-2,3,5,6,7,7a-hexahydro-1*H*-inden-1-yl)methylene)hydrazine-1-carboximidamide (**41**): To a solution of the ketone-aldehyde **31** (57 mg, 0.32 mmol) in EtOH (6 mL), 2.00 equiv of the aminoguanidine hydrochloride (70 mg, 0.64 mmol) and 1 equiv of 2 N HCl (2 N, 1.0 equiv) in EtOH were added. The mixture was heated to reflux for 45 min, then cooled to room temperature, concentrated under reduced pressure, and the residue was washed with hexanes and EtOAc (8:2, 30 mL) to obtain the corresponding amidinohydrazone **41** (70 mg, 78%) as a yellow foam. Spectroscopic data for compound **41** were consistent with reported data [27,28].

(*E+Z*)-2-((6-(2-Carbamimidoylhydrazineylidene)-8a-methyl-1,2,3,4,6,7,8,8a-octahydronaphthalen-1-yl)methylene)hydrazine-1-carboximidamide (**42**): Compound **42** was synthesized using the same procedure employed for **41** using ketone-aldehyde **37 (**40 mg, 0.20 mmol), aminoguanidine hydrochloride (48 mg, 0.43 mmol), and 2 N HCl (2 N, 1.0 equiv). After the completion of the reaction, the residue was washed with hexanes and EtOAc (8:2, 30 mL) to obtain the corresponding amidinohydrazone **42** (51 mg, 80%) as a white foam: ^1^H NMR (400 MHz, CD_3_OD) δ 7.59 (dq, *J* = 15.2, 8.6, 8.1 Hz, 2H), 7.29 (s, 1H), 6.43 (s, 0.2H), 5.95 (s, 0.8H), 3.22–2.92 (m, 1H), 2.76 (d, *J* = 17.1 Hz, 1H), 2.57–2.16 (m, 4H), 1.81 (dt, *J* = 74.0, 13.4 Hz, 5H), 1.49–1.03 (m, 4H). HRMS (ESI) calcd for C_14_H_25_N_8_ (M + H)^+^ 305.2202, found 305.2204.

5-Hydroxy-4a-methyl-4,4a,5,6,7,8-hexahydronaphthalen-2(3*H*)-one (**43**): To a solution of ketone **3** (0.50 g, 2.80 mmol) in EtOH (18 mL), NaBH_4_ (40 mg, 1.10 mmol) was added at 0 °C, and the reaction was stirred for 5 min. AcOH (0.5 mL) was then introduced to the reaction mixture and stirred for an additional 5 min. Volatiles were removed under reduced pressure. To the resulting residue, EtOAc (30 mL) was added, followed by washing with brine and drying over Na_2_SO_4_. Purification through flash column chromatography (EtOAc: hexanes 1:3) yielded hydroxy ketone **43** (0.41 g, 82%) as a colorless liquid: ^1^H NMR (400 MHz, CDCl_3_) δ 5.74 (d, *J* = 1.8 Hz, 1H), 3.39 (dd, *J* = 11.6, 4.3 Hz, 1H), 2.66–2.51 (m, 1H), 2.48–2.23 (m, 3H), 2.25–2.08 (m, 2H), 1.90–1.74 (m, 3H), 1.73–1.61 (m, 1H), 1.46–1.28 (m, 1H), 1.16 (s, 3H); ^13^C NMR (100 MHz, CDCl_3_) δ 200.0, 169.1, 125.3, 78.1, 41.7, 34.2, 33.7, 32.1, 30.2, 23.2, 15.3.

5-Azido-4a-methyl-4,4a,5,6,7,8-hexahydronaphthalen-2(3*H*)-one (**44**): Compound **44** was prepared following general procedure **D** using triphenylphosphine (0.16 g, 0.64 mmol), diethyl azodicarboxylate (0.11 mL, 0.64 mmol, 40% solution in toluene), alcohol **43** (0.10 g, 0.58 mmol), and diphenyl phosphoryl azide (0.13 mL, 0.64 mmol) to obtain **44** (0.057 g, 50%) as a yellow liquid. ^1^H NMR (400 MHz, CDCl_3_) δ 5.81 (d, *J* = 1.9 Hz, 1H), 3.56 (t, *J* = 2.8 Hz, 1H), 2.55–2.35 (m, 4H), 2.33–2.24 (m, 1H), 2.17–1.93 (m, 2H), 1.90–1.69 (m, 2H), 1.59–1.50 (m, 1H), 1.29 (s, 3H); ^13^C NMR (100 MHz, CDCl_3_) δ 198.8, 165.7, 126.9, 68.6, 40.1, 34.0, 32.1, 31.4, 25.5, 22.4, 20.4.

4a-Methyl-5-(4-phenyl-1*H*-1,2,3-triazol-1-yl)-4,4a,5,6,7,8-hexahydronaphthalen-2(3*H*)-one (**45**): To a mixture of azide **44** (49 mg, 0.22 mmol) and phenylacetylene (36 mg, 0.33 mmol) in DMF (2 mL), sodium ascorbate (17 mg, 0.088 mmol) in H_2_O (1 mL) was added and stirred for 2 min at ambient temperature. Then, a CuSO_4_.5H_2_O (11 mg, 0.044 mmol) in H_2_O (1 mL) was added to the above mixture. The reaction was stirred at room temperature for 12 h, quenched with water (3 mL), and extracted with EtOAc (3 × 5 mL). The combined organic layers were dried over Na_2_SO_4_, filtered, and concentrated under reduced pressure to give a greenish residue, which was purified by flash chromatography (silica gel, EtOAc/hexanes, 4:6) to give **45** (52 mg, 71%) as a white solid: mp 178–180 ^°^C; ^1^H NMR (400 MHz, CDCl_3_) δ 7.79–7.72 (m, 2H), 7.68 (s, 1H), 7.41–7.33 (m, 2H), 7.32–7.25 (m, 1H), 5.93 (d, *J* = 1.5 Hz, 1H), 4.79 (t, *J* = 3.6 Hz, 1H), 2.71–2.27 (m, 4H), 2.22–2.12 (m, 1H), 2.10–1.81 (m, 4H), 1.51 (s, 3H), 1.49–1.41 (m, 2H); ^13^C NMR (100 MHz, CDCl_3_) δ 197.6, 164.9, 147.6, 130.3, 128.9, 128.4, 127.2, 125.8, 119.6, 66.5, 40.0, 33.6, 31.0, 30.8, 26.9, 24.0, 20.5; HRMS (ESI) calcd for C_19_H_22_N_3_O (M + H)^+^ 308.1763, found 308.1776.

#### 4.1.3. Sperm Isolation and Studies in Mice

All experimental protocols involving animals used in this work were approved by the University of Kansas Medical Center Institutional Animal Care and Use Committee (ACUP number: 22-09-26, 10/2022). C57BL/6 mice were purchased from Harlan (Indianapolis, IN, USA). Spermatozoa were isolated from the cauda of adult mouse epididymis in Whitten’s medium, containing 100 mM NaCl, 4.7 mM KCl, 1.2 mM KH_2_PO_4_, 1.2 mM MgSO_4_, 5.5 mM glucose, 0.8 mM pyruvic acid, 4.8 mM lactic acid, and 40 mM Hepes, as previously described [19].

#### 4.1.4. Sperm Motility Assays

Spermatozoa (1 × 10^6^ cells) were incubated at 37 °C in 100 μL of Whitten’s medium, with the addition of 1.7 mM CaCl_2_ and in the absence and presence of different amounts of the tested compounds for 60 min. Then, cells were labeled with the fluorescent nucleic acid stain SYTO 21, which allows for the tracking of the movement of the cell head. After 1 min of incubation with the dye, 5.5 μL aliquots from each sample were taken and placed into a glass cell chamber (Leja Products B.V., Nieuw-Vennep, The Netherlands). Sperm were maintained at 37 °C on a heated platform and viewed with an Olympus BX51 microscope (Olympus, Center Valley, PA, USA) through a 20× phase objective. Images of the moving sperm were captured using a CCD camera. Samples were analyzed by CASA using the Minitube SpermVision Digital Semen Evaluation system (version 3.5; Penetrating Innovations, Verona, WI, USA). An average of 200 cells/field were captured, at a rate of 30 frames per field, and a total of 10 fields in each sample were analyzed. Total sperm motility was obtained from sperm maintained in a non-capacitating medium, while for assessing the hyperactive pattern of motility, typical of sperm capacitation, cells were incubated in Whitten’s medium with the addition of 1.7 mM CaCl_2_, 25 mM sodium bicarbonate, and 0.5% bovine serum albumin.

#### 4.1.5. Insect Cells and Viral Infections

The NKA α1 and α4 isoforms were expressed in Sf9 insect cells as previously described [15,45]. Briefly, cells were cultured in suspension at 27 °C in Grace’s medium (JRH Biosciences, Lenexa, KS, USA) with 3.3 g/L of lactalbumin hydrolysate and 3.3 g/L of yeastolate and supplemented with 10% (*v*/*v*) fetal bovine serum, 100 units/mL of penicillin, 100 μg/mL of streptomycin, and 0.25 μg/mL of fungizone. For infections, cells were placed on 150 mm Petri dishes and infected with baculoviruses coding for the NKA α1β1 and α4β1 isozymes. Three days after infection, cells were scraped from the culture plates, centrifuged at 1500× *g* for 10 min, and washed twice in 10 mM imidazole hydrochloride (pH 7.5) and 1 mM EGTA (suspension buffer). Cells were then placed in a suspension buffer plus the addition of 0.32 M sucrose and stored at −20 °C or directly used for assays.

#### 4.1.6. Na,K-ATPase Assay

The Na,K-ATPase activity of insect cell homogenates was determined by measuring the initial rate of release of ^32^P_i_ from γ[^32^P]-ATP as described [45]. The incubation medium (0.25 mL final volume) contained 120 mM NaCl, 30 mM KCl, 3 mM MgCl_2_, 0.2 mM EGTA, 30 mM Tris-HCl (pH 7.4), and different concentrations of the corresponding compounds. The assay was started by the addition of ATP with 0.2 μCi γ[^32^P]-ATP (2 mM final concentration). After 30 min of incubation at 37 °C, the reaction was stopped with trichloroacetic acid, and 5% ammonium molybdate in 4 N H_2_SO_4_ was used to complex the ^32^P_i_ released in the Na,K-ATPase reaction. The phosphomolybdate was extracted with isobutanol as described, and 170 μL of the organic phase was taken and subjected to liquid scintillation counting. Enzymatic activity was determined as the difference in ATP hydrolysis in the absence and presence of 1 mM ouabain.

#### 4.1.7. Reversibility of Compounds In Vitro

Mouse epididymal sperm were treated in the absence or presence of compounds **13** or **45** for one hour in Whitten’s medium. After an initial measurement of sperm motility using CASA, the sperm was washed by centrifugating the sample at 300× *g* for 3 min and resuspension in fresh Whitten’s medium. Then, sperm motility was determined again using CASA for the different times and for up to 180 min.

### 4.2. ADME and Toxicity Studies

Solubility, permeability, compound stability, metabolic stability, and toxicity studies were performed at Pharmaron (Waltham, MA, USA).

#### 4.2.1. Solubility Determination

The stock solutions of **11** and **45** were prepared in DMSO at a concentration of 30 mM, and the stock solution of the control compound was prepared in DMSO at a concentration of 30 mM. Progesterone was used as a positive control in the assay. An amount of 10 µL of stock solution of each compound was placed in order into their proper 96-well rack, followed by adding 990 µL of PBS at pH 7.4 into each vial of the capless Solubility Sample plate. This study was performed in duplicate. One stir stick was added to each vial and then sealed using a molded PTDE/SIL 96-Well Plate Cover (Pharmaron, Waltman, MA, USA). The Solubility Sample plate was transferred to the Thermomixer Comfort plate shaker and incubated at RT for 2 h with shaking at 1100 rpm. After 2 h of incubation, stir sticks were removed using a big magnet, and all samples from the Solubility Sample plate were transferred into the filter plate. All the samples were filtered using a vacuum manifold. The filtered samples were diluted with methanol. The soluble fraction (filtrate) was then analyzed by LC-MS/MS, and the compound concentration was determined using 0.3 μM standard solutions prepared from DMSO stocks. The experimental details are reported in the Supporting Information (Table S1).

#### 4.2.2. In Vitro Permeability Assay

The in vitro permeability and potential to be transported by P-gp (P-glycoprotein) were determined in a Caco-2 or MDCK cell line. Values are reported for a mass recovery > 60%. The efflux ratio was determined as follows: *P*^app^_(B-A)_/*P*_app(A-B)._ The experimental details are reported in the Supporting Information (Tables S2–S5).

#### 4.2.3. Metabolic Stability in Hepatocytes

The metabolic stability of a test compound is tested using liver hepatocytes from humans, rats, and mice incubated up to 120 min at 37 °C with 1 μM of the test compound. The in vitro metabolic half-life (*t*_1/2_) is calculated using the slope of the log−linear regression from the percentage of the parent compound remaining versus the time relationship (*κ*). The experimental details are reported in the Supporting Information (Tables S6 and S7).

#### 4.2.4. hERG Inhibition Patch-Clamp Assay

The potential inhibitory effect of **45** on the human Ether-à-go-go related gene (hERG) channel was evaluated using a manual patch-clamp system. HEK293 cell line stably transfected with the hERG gene was employed in this study, and dofetilide was used as a positive control to validate the assay’s performance. The experimental details are reported in the Supporting Information).

#### 4.2.5. Mini Ames Test

The mutagenic potential of **45** was evaluated using the Mini Ames test, which measures reverse mutations at the histidine locus in *Salmonella typhimurium* strains TA98 and TA100. The assay was conducted with and without exogenous metabolic activation (β-naphthoflavone/phenobarbital-induced rat liver S9). The experimental details are reported in the Supporting Information).

#### 4.2.6. In Vivo Pharmacokinetic Study

Male CD-1 mice (approximately 6–8 weeks old, weighing 20–25 g, supplied by Vital River, Beijing, China) were used for the pharmacokinetic studies. Each mouse (n = 2) received either a bolus intravenous injection via the tail vein or oral gavage administration of the test compound. The formulation consisted of 20% HP-b-CD in PBS (pH 7.4), administered at a dose level of 10 mg/kg. The intravenous dose volume was 5 mL/kg, while the oral dose volume was 10 mL/kg. Blood samples (approximately 30 μL) were collected at 15 min, 30 min, and 1, 3, and 5 h in tubes containing potassium EDTA as an anticoagulant via direct vein puncture of the dorsal metatarsal vein. At the end of the study, the mice were euthanized by the inhalation of rising carbon dioxide concentrations. Samples were centrifuged at 4000× *g* for 5 min at 4 °C to obtain plasma. The plasma samples were stored at −75 ± 15 °C before analysis. Plasma samples were analyzed using the LC/MS/MS method. WinNonlin (PhoenixTM, version 8.3) software was employed for pharmacokinetic analysis calculations. The experimental details are reported in the Supporting Information.

### 4.3. In Vitro Fertilization Assays

In vitro fertilization assays (IVF) followed standard procedures with some modifications [22]. Oocytes were obtained from super-ovulated female mice after stimulation with 5 IU of PG 600 (Intervet, Rahway, NJ, USA) and 5 IU of human chorionic gonadotrophin. Spermatozoa were collected from the cauda epididymis after swim-up. Sperm and oocytes, at a ratio of 500:1, were incubated in Cook’s medium (Research Vitro Fert K-RVFE-50; Cook Medical, Bloomington, IN, USA) for 6 h at 37 °C, with 5% CO_2_ and 5% O_2_ under oil. After removing and washing the oocytes in Cook’s medium, they were cultured overnight. The next day, fertilization was determined by the development of embryos at the two-cell stage.

### 4.4. Testing of Compounds in Mice

Compound **45** was administered to mice by intraperitoneal injection at a dose of 40 mg/kg of body weight for three days. This protocol was used based on our previous results [24]. At day 3 of treatment, the animals were sacrificed, and sperm was collected from the caudal section of the epididymis. Sperm was incubated under non-capacitating and capacitating conditions, and total and hyperactive sperm motility was determined using CASA.

### 4.5. Data Analysis

The statistical significance of the differences between the effect of the compounds, depending on dose, time of action, and isoform selectivity, was determined by ANOVA, followed by Tukey’s post-test for multiple comparisons. Statistical significance was defined as *p* < 0.05. Comparisons of the effect of the compounds on sperm viability, sperm motility after in vivo administration to mice, and in vitro fertilization assays were assessed by Student’s *t-*test. For the calculation of IC_50_ values for sperm motility inhibition and the response of NKA activity to the compounds, Marquardt least square nonlinear regression was used as previously published [15,45]. Dose–response curves for the inhibition of NAK isoform activity by the compounds were best fitted by a single-site model. In all cases, the data acquired were analyzed with GraphPad Prism 9.0 software.

## 5. Conclusions

In conclusion, we have synthesized a novel series of simplified, non-steroidal compounds with potency and excellent selectivity for the NKAα4 isoform. These compounds effectively disrupt sperm motility, hyperactivation, and fertilizing capacity. Their streamlined chemical scaffolds enable rapid synthesis in just 3–4 steps, making them attractive candidates for the development of a highly specific non-steroidal male contraceptive. Further optimization of compound **45** to address its metabolic liabilities is ongoing, and the results will be reported in due course.

## Figures and Tables

**Table 1:** IC_50_ values and relative inhibition effect of simplified steroid analogs on mouse sperm motility.

** 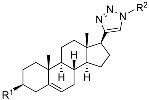 **				
**Compound**	**R^1^**	**R^2^**	**Maximal Motility** Inhibition (%) *	**IC_50_ (pM)**
**13**	OH	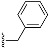	41	24
**14**	OH	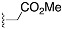	46	26
**15**	OH	H	42	19
**19**	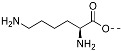	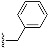	37	32
**24**		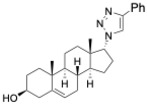	50	15
**26**		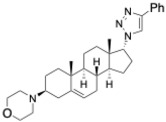	40	31
**30**		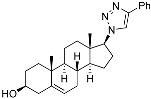	ND **	ND **

* Maximal sperm motility inhibition at the nanomolar range concentration of the compounds. ** Not determined due to the limited solubility of the compound.

**Table 2:** IC_50_ values and relative inhibition effect of non-steroidal small molecules on mouse sperm motility.

Compound	Structure	Maximal Motility Inhibition (%) *	IC_50_ (pM)
**33**	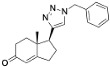	48	23
**35**	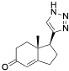	47	71
**39**	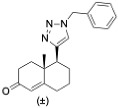	ND **	ND **
**40**	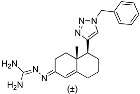	49	60
**41**	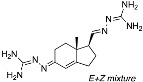	44	37
**42**	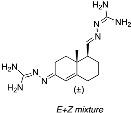	49	302
**45**	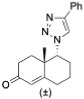	51	48

* Maximal sperm motility inhibition at the nanomolar range concentration of the compounds. ** Not determined (ND) due to the limited solubility of the compound.

**Table 3:** NKAα4 and α1 isoform potencies, permeability, and solubility of compounds **13** and **45**.

Compound	NKA Isoform Specificity (IC_50_ M) ^a^		Permeability ^b^ P_app_ (A-B)	Solubility ^c^
	NKAα4	NKAα1		
**13**	1.0 ± 4.2 × 10^−10^	>10^−4^	0.6 × 10^−6^ cm/s	0.300 μM
**45**	2.7 ± 7.9 × 10^−9^	>10^−4^	24 × 10^−6^ cm/s	256 μM

^a^ The IC_50_ values were calculated from dose–response curves for the inhibition of α1β1 and α4β1 expressed in Sf9 insect cells. Values ± SEM of three independent experiments. ^b^ The permeability for **13** was evaluated using the MDCK cell line in the presence of 0.5% BSA, while the permeability of **45** was assessed using the Caco-2 cell line assay. We selected different cell lines based on the solubility profiles of the compounds. MDCK cells were used for poorly soluble compound **13** due to their robustness and tolerance for lower solubility conditions. For soluble compound **45**, we used Caco-2 cells, which better mimic the human intestinal epithelium and are widely accepted for modeling oral absorption and permeability. ^c^ The solubility of the test compound was determined in phosphate-buffered saline (PBS) at pH 7.4.

**Table 4:** In vitro ADME and toxicity assessment for compound **45**.

Stability: 96% ^a^		
Log D: 2.66 ^b^		
hERG ^d^: >30 μM		
Mini Ames ^e^: negative		
Metabolic Stability in Hepatocytes (60 min)		
Human ^c^	Rat ^c^	Mouse ^c^
39%	10%	10%
T_1/2_ min 38	T_1/2_ min 19	T_1/2_ min 18
In vitro Cl_int_ (μL/min/10^6^ cells): 36.04	In vitro Cl_int_ (μL/min/10^6^ cells): 72.15	In vitro Cl_int_ (μL/min/10^6^ cells): 77.11
Scale-up Cl_int_ (mL/min/kg) 91.69	Scale-up Cl_int_ (mL/min/kg) 337.7	Scale-up Cl_int_ (mL/min/kg) 10.91

^a^ The stability in PBS (pH 7.4) is reported as the percentage of the compound remaining after 60 min. ^b^ The LogD was measured in a 1-octanol/phosphate buffer system at pH 7.4. ^c^ The metabolic stability was evaluated by incubating the compound with human, rat, and mouse liver hepatocytes for 120 min at 37 °C; Table 4 presents the percentage of compound remaining after 60 min. ^d^ The hERG inhibitory effect was assessed using a manual patch-clamp assay. ^e^ The Mini Ames assay was performed with *Salmonella typhimurium*.

**Table 5:** In vivo pharmacokinetic parameters of **45** in CD1 mice.

Parameter	Single-Dose Administration	
	IV 10 mg/kg	PO 10 mg/kg
C_0_ (ng/mL)	1981	-
C_max_ (ng/mL)	124	79.7
T_max_ (h)	-	0.50
T_1/2_ (h)	0.125	1.07
AUC_last_ (h·ng/mL)	559	74.3
AUC_inf_ (h·ng/mL)	561	77.0
CL (mL/min/kg	297	-
V_ss_ (mL/kg)	1.10	-
F%	-	13.7

## Data Availability

All data generated in this study are included in the article and its Supporting Information. Further inquiries can be directed to the corresponding authors.

## Supplementary Materials

The following supporting information can be downloaded at: https://www.mdpi.com/article/10.3390/ijms26125646/s1.

## Abbreviations

The following abbreviations are used in this manuscript:

ADME	Absorption, distribution, metabolism, and excretion
ATP	Adenosine triphosphate
AUC	Area under the curve
BSA	Bovine serum albumin
*C*max	Maximum plasma concentration
Clint	Intrinsic clearance
Cl	Clearance
DCC	*N*,*N*′-Dicyclohexylcarbodiimide
DCM	Dichloromethane
DEAD	Diethyl azodicarboxylate
DMAP	*N*,*N*-Dimethylaminopyridine
DMF	*N*,*N*-Dimethylformamide
DPPA	Diphenylphosphoryl azide
EtOAc	Ethyl acetate
EtOH	Ethanol
F%	Oral bioavailability
LCMS	Liquid chromatography−mass spectrometry
MeOH	Methanol
MDCK	Madin Darby canine kidney
min	Minutes
PK	Pharmacokinetics
Papp	Apparent permeability coefficient
rt	Room temperature
SAR	Structure–activity relationship
SD	Standard deviation
TBDPSCl	*tert*-Butyldiphenylchlorosilane
THF	Tetrahydrofuran
*T*1/2	Half-life
*T*max	Time to reach maximum plasma concentration
TPP	Triphenylphosphine
UPLC	Ultraperformance liquid chromatography
V_ss_	Volume of distribution
