# Supplementary material for: Strategies to Develop Na,K-ATPase-α4 Inhibitors as Male Contraceptives
Source: Int J Mol Sci. 2025 Jun 12;26(12):5646. doi: 10.3390/ijms26125646 (PMC12192727; doi:10.3390/ijms26125646)
Supplement: Supplementary file 1 [file ijms-26-05646-s001.zip › ijms-3661160-supplementary.pdf]

Supporting Information

Strategies to develop Na,K-ATPase-α4 inhibitors as male contraceptives

Shameem Sultana Syeda[1], Gladis Sánchez,[2] Jeffrey P McDermott,[2] Narsihmulu Cheryala,[1] Henry Wong,[1] Gunda I Georg[1]* and Gustavo Blanco[2],*

[1] Department of Medicinal Chemistry, College of Pharmacy and Institute for Therapeutics Discovery and Development, University of Minnesota, Minneapolis, MN 55414, USA.
[2] Department of Cell Biology and Physiology, University of Kansas Medical Center, Kansas City, KS 66160, USA.
* Correspondence: georg@umn.edu (G. I. G.); gblanco@kumc.edu (G. B.); Tel.: +1 (913) 588-7405 (G. B.).

Solubility

Table S1. Solubility Results of Test Compound and Control Compound in PBS pH 7.4

Compound ID	Solubility (µM)
Progesterone	18.0
**13**	0.300
**45**	256

Preparation of Stock Solutions: The stock solutions of test compounds were prepared in DMSO at the concentration of 30 mM, and the stock solution of control compound was prepared in DMSO at the concentration of 30 mM. Progesterone was used as positive control in the assay.

Solubility Determination: 10 µL stock solution of each compound was placed in order into their proper 96-well rack, followed by adding 990 µL of PBS at pH 7.4 into each vial of the cap-less Solubility Sample plate. This study was performed in duplicate. One stir stick was added to each vial and then vials were sealed using a molded PTDE/SIL 96-Well Plate Cover. The Solubility Sample plate was transferred to the Thermomixer Comfort plate shaker and incubated at RT for 2 hours with shaking at 1100 rpm. After 2 hours incubation, stir sticks were removed using a big magnet and all samples from the Solubility Sample plate were transferred into the filter plate. All the samples were filtered by using the Vacuum Manifold. The filtered samples were diluted with methanol. The dilution factor might be changed according to the solubility value and the LC/MS signal response. To prepare 0.3 µM Standards (STD), 30 mM DMSO compound stock solutions were diluted with DMSO to 300 µM and then diluted with methanol to obtain 0.3 µM STDs. Samples were analyzed by LC-MS/MS. All calculations were carried out using Microsoft Excel. The solution filtered was analyzed and quantified against a standard of known concentration in DMSO using LC coupled with Mass spectral peak identification and quantitation. The solubility values of the test compounds were calculated as follow

$$[Sample] = \frac{AREA_{Sample} \times INJ\ VOL_{Std} \times DF_{Sample} \times [STD]}{AREA_{Std} \times INJ\ VOL_{Sample}}$$

DF means the dilution factor

1. Chromatographic conditions

LC system: Shimadzu

Apparatus: Triple Quad™ 5500 instrument from AB Inc (Canada) with an ESI interface

Column: XSelect Hss T3 2.5µ (2.1×50 mm) Column XP coupled with preguard column

Mobile phase: 0.1% formic acid in acetonitrile (A) and 0.1% formic acid in water (B)

Column temperature: 40°C

Elution Rate:

Time (min)	0	0.5	0.8	0.81	1.0
% B	5	100	100	5	5

Injection volume: 3 μL
Elution rate: 0.65mL/min

2. MS parameters

Ion source: Turbo spray
Ionization model: ESI
Scan type: MRM
Collision gas: 10 L/min
Curtain gas: 30 L/min
Nebulize gas: 60 L/min
Auxiliary gas: 60 L/min
Temperature: 550°C
Ionspray voltage: + 5500 v (positive MRM)

Compound ID	Q1 (*m*/*z*)	Q3 (*m*/*z*)	DP (v)	EP (v)	CE (v)	CXP (v)
**13**	432.3	158.0	120	10	35	20
**45**	308.3	121.1	80	10	25	10

Compound **13** and **45** Permeability:

Table S2. Permeability Results of Test Compounds in MDCK Cells

Compound ID	BSA (%)	$P_{app\ (A-B)}$ (10^{-6}, cm/s)	$P_{app\ (B-A)}$ (10^{-6}, cm/s)	Efflux Ratio	Recovery (%)AP-BL	Recovery (%)BL-AP
Metoprolol	0	21.57	16.99	0.79	83.49	97.11
Digoxin	0	1.01	3.29	3.26	83.95	106.43
Imatinib	0	8.95	12.14	1.36	59.75	86.83
**13**	0.5	0.63	0.78	1.24	70.16	88.09

Table S3. The Assessment of MDCK Cell Monolayer Integrity

Compound ID	BSA (%)	$TEER_{A-B}$ ($W \times cm^2$)	$TEER_{B-A}$ ($W \times cm^2$)	LY Leakage $_{A-B}$ (%)	LY Leakage $_{B-A}$ (%)
Metoprolol	0	108	112	0.23	0.23
Digoxin	0	106	119	0.16	0.23
Imatinib	0	104	94	0.15	0.54
**13**	0.5	112	116	0.14	0.13

The apparent permeability coefficient (P_{app}), in units of centimeter per second, can be calculated for MDCK drug transport assays using the following equation:

$$P_{app} = (V_A \times [drug]_{acceptor})/(Area \times Time \times [drug]_{initial,\ donor})$$

Where V_A is the volume (in mL) in the acceptor well, Area is the surface area of the membrane (0.143 cm^2 for Transwell-96 Well Permeable Supports), and time is the total transport time in seconds.

The efflux ratio will be determined using the following equation:

$$\text{Efflux Ratio} = P_{app(B-A)}/P_{app(A-B)}$$

Where $P_{app\ (B-A)}$ indicates the apparent permeability coefficient in basolateral to apical direction, and $P_{app\ (A-B)}$ indicates the apparent permeability coefficient in apical to basolateral direction.

The recovery can be determined using the following equation:

$Recovery\% = (V_A \times [drug]_{acceptor} + V_D \times [drug]_{donor}) / (V_D \times [drug]_{initial,\ donor})$

Where V_A is the volume (in mL) in the acceptor well (0.235 mL for Ap→Bl flux, and 0.075 mL for Bl→Ap), V_D is the volume (in mL) in the donor well (0.075 mL for Ap→Bl flux, and 0.235 mL for Bl→Ap)

The leakage of Lucifer Yellow, in unit of percentage (%), can be calculated using the following equation:

$\%LY\ leakage = 100 \times [LY]_{acceptor} \times 0.3 / ([LY]_{donor} \times 0.1 + [LY]_{acceptor} \times 0.3)$

LY leakage of <1% is acceptable to indicate the well-qualified MDCK/ Caco-2 monolayer.

Table S4. Permeability Results of Test Compounds in Caco-2 Cells

Compound ID	$P_{app\ (A-B)}$ (10^{-6}, cm/s)	$P_{app\ (B-A)}$ (10^{-6}, cm/s)	Efflux Ratio	Recovery (%)AP-BL	Recovery (%)BL-AP
Propranolol	24.42	16.43	0.67	72.75	86.63
Digoxin	0.63	18.76	30.00	88.71	107.44
Prazosin	7.59	24.32	3.20	71.43	85.05
**45**	23.97	11.84	0.49	64.28	77.86

Table S5. The Assessment of Caco-2 Cell Monolayer Integrity

Compound ID	$TEER_{A-B}$ (W × cm^2)	$TEER_{B-A}$ (W × cm^2)	LY $Leakage_{A-B}$ (%)	LY $Leakage_{B-A}$ (%)
Propranolol	713	725	0.55	0.62
Digoxin	674	768	0.29	0.12
Prazosin	683	836	0.13	0.10
**45**	797	794	0.40	0.63

The apparent permeability coefficient (Papp), Efflux ratio (ER), recovery percentage, and LY leakage percentage can be calculated for Caco-2 Cell drug transport assays using the above equations.

Compound 45 Metabolic Stability:

Table S6. Metabolic Stability of Test Compounds in Different Species of Hepatocytes

Compound ID	Species	*In vitro* $T_{1/2}$ (min)	*In vitro* Cl_{int} (μL/min/10^6 cells)	Scale-up Cl_{int} (mL/min/kg)	Hepatic Extraction Ratio (E)	Predicted hepatic CL (mL/min/kg)
Verapamil	Human	32.22	43.01	109.44	0.84	17.41
	Rat	19.63	70.62	330.48	0.86	47.30
	Mouse	17.52	79.13	934.68	0.91	82.10
**45**	Human	38.46	36.04	91.69	0.82	16.89
	Rat	19.21	72.15	337.65	0.86	47.44
	Mouse	17.97	77.11	910.91	0.91	81.91

Table S7. Metabolic Stability of Test Compounds in Different Species of Hepatocytes

Compound ID	Species	Assay Format	Remaining Percentage (%)					
			0 min	15 min	30 min	60 min	90 min	120 min
Verapamil	Human	Hepatocytes	100.00	68.67	50.56	27.47	17.29	6.57
		Boiled hepatocytes	100.00	103.69	105.49	107.70	108.83	88.30
	Rat	Hepatocytes	100.00	46.42	21.83	8.08	2.99	1.36
		Boiled hepatocytes	100.00	101.75	100.07	104.62	101.66	107.75
	Mouse	Hepatocytes	100.00	47.95	21.18	7.67	2.70	0.73
		Boiled hepatocytes	100.00	102.10	94.40	94.97	97.09	94.98
**45**	Human	Hepatocytes	100.00	83.47	64.22	39.26	22.24	11.51
		Boiled hepatocytes	100.00	105.47	106.55	108.22	105.29	107.99
	Rat	Hepatocytes	100.00	51.55	26.35	9.77	3.36	1.25
		Boiled hepatocytes	100.00	101.65	104.87	104.47	104.04	104.50
	Mouse	Hepatocytes	100.00	54.16	31.17	10.30	3.01	0.80
		Boiled hepatocytes	100.00	104.93	101.77	101.78	101.42	100.86

hERG inhibition patch-clamp assay

The potential inhibitory effect of test articles on the human Ether-à-go-go related gene (hERG) channel was evaluated using a manual patch-clamp system. The HEK293 cell line, which was stably transfected with the hERG gene, was employed in this study, and Dofetilide served as a positive control to ensure the assay's quality. The results are presented in the following table. Based on the findings and the criteria described in the report, the test article **45** is considered a weak inhibitor of the hERG channel.

Test article	hERG IC_{50} [μM]
**45**	> 30 [(1)]
Dofetilide	0.014 [(2)]

Note: (1) The IC_{50} > 30 μM is reported if the maximal percentage of hERG current inhibition in presence of 30 mM of test compound is lower than 50%.
A generally acceptable ranking system used to identify the potency of a test compound inhibiting hERG channel is list as follows:

a) Low: IC_{50} > 10 μM;
b) Moderate: 1 μM < IC_{50} < 10 μM;
c) High: IC_{50} < 1 μM;

The FDA criterion for defining a drug as hERG-positive is when IC_{50} < 1 μM.

Snap Shot PK of 45 in CD1-Mice

**N / Treatment**	2
**Sex**	Male
**Doses**	IV 10 mg/Kg<br>PO 10 mg/Kg
**Formulations**	IV 20% HP-b-CD in PBS

	PO 20% HP-b-CD in PBS
**Dosing Solutions:**	IV 2 mg/mL<br>PO 2mg/mL
**Blood Sampling @**	IV&PO Plasma: 0.5, 1, 3, and 5 h post dose

Bioanalytical Assay:

HPLC	Instrument: SHIMADZU (LC-40D XS, Serial NO.L22426002383 AE and L22426102679 AE; DGU-405 Serial NO.L22176052658 US; CBM-40 Serial NO.L22116104417 CD; SIL-40C, Serial NO: L22456101599 AE; CTO-40C, Serial NO: L22246108429 IK); Plate Changer (Serial No. L22226100904 CZ and Serial No. L22226100905 CZ)	
MS	AB API 6500 + LC/MS/MS instruments (Serial NO.DZ249952308)	
Column	HALO, 90A, C18, 2.7μm,3x30 mm	
Mobile Phase	A	5% Acetonitrile in Water (0.1%Formic acid)
	B	95% Acetonitrile in Water (0.1%Formic acid)
Quantification	Internal Standard Method	
**Bioanalysis**	10 uL of plasma sample + 5 uL of blank solution + 200 uL of acetonitrile for PPE (protein precipitation extraction)	

45 IV and PO Pharmacokinetic Profile in CD1 Mouse

45 IV Plasma concentration-time data

Blue data were included in terminal elimination $T_{1/2}$

IV Dose **10** **mg/kg**

**Time (h)**	**Concentration (ng/mL) Mean_Plasma**
0.5	124
1	7.76
3	BLOQ
5	BLOQ

45 PO Plasma concentration-time data

Blue data were included in terminal elimination $T_{1/2}$

PO Dose **10** **mg/kg**

**Time (h)**	**Concentration (ng/mL) Mean_Plasma**
0.5	79.7
1	22.8
3	2.14
5	1.71

Summary of **45** IV pharmacokinetic parameters

IV Dose 10 mg/kg

PK parameters	Unit	Mean_Plasma
Cl_obs	mL/min/kg	297
T1/2	h	0.125
C0	ng/mL	1981
AUC_{last}	h*ng/mL	559
AUC_{Inf}	h*ng/mL	561
AUC_%Extrap_obs	%	0.250
MRT_{Inf}_obs	h	0.0617
AUC_{last}/D	h*mg/mL	55.9
V_{ss}_obs	L/kg	1.10

Summary of **45** PO pharmacokinetic parameters

PO Dose 10 mg/kg

PK parameters	Unit	Mean_Plasma
$T_{1/2}$	h	1.07
T_{max}	h	0.500
C_{max}	ng/mL	79.7
AUC_{last}	h*ng/mL	74.3
AUC_{Inf}	h*ng/mL	77.0
AUC_%Extrap_obs	%	3.43
MRT_{Inf}_obs	h	1.13
AUC_{last}/D	h*mg/mL	7.43
F	%	13.7

Notes

1. BLOQ = 0.5 ng/mL for plasma
2. NA – Not available
3. PK parameters were estimated by non-compartmental model using WinNonlin 8.3
4. The bioavailability (F%) was calculated as following:
   AUClast-PO/AUCINF-PO > 80%: F=(AUCINF-PO*DoseIV)/(mean AUCINF- IV*DosePO)

Chemistry

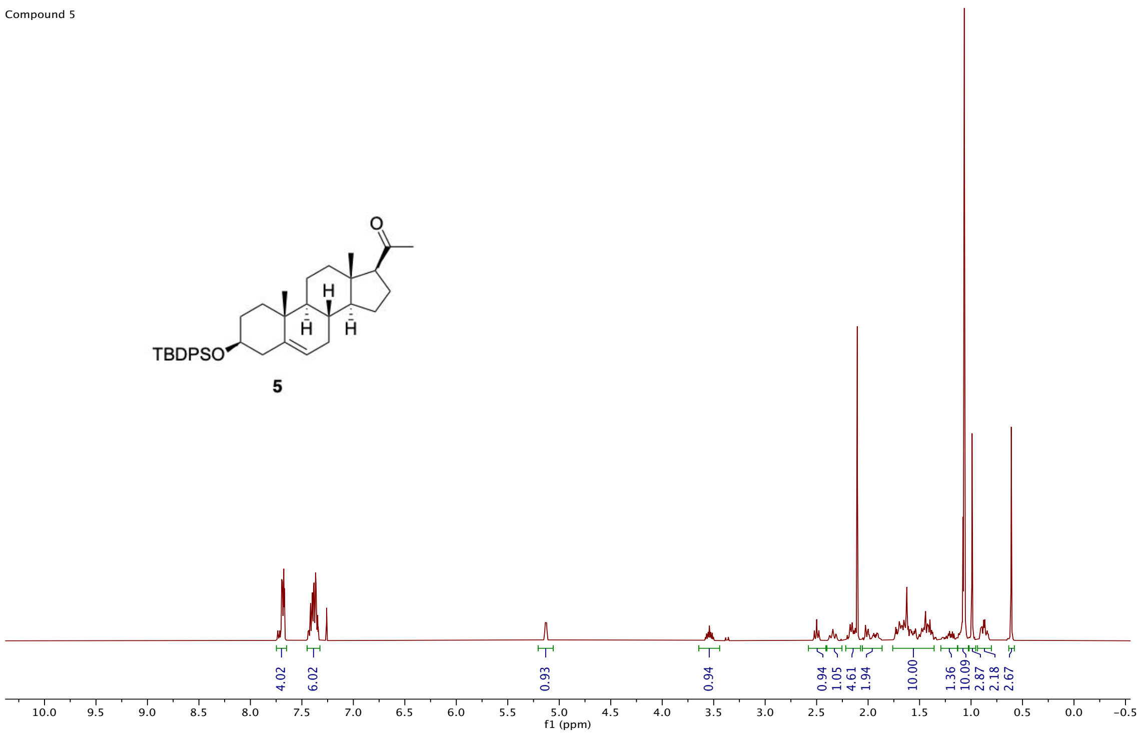

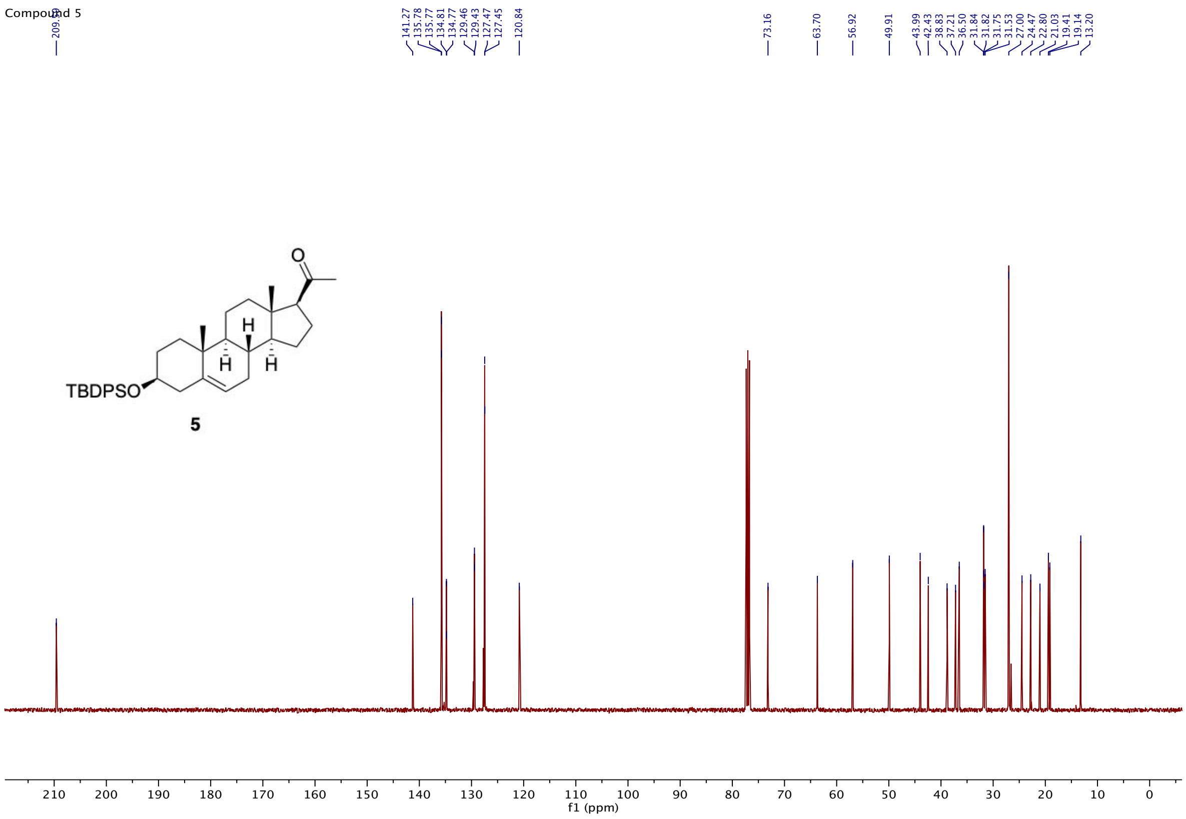

Compound 6

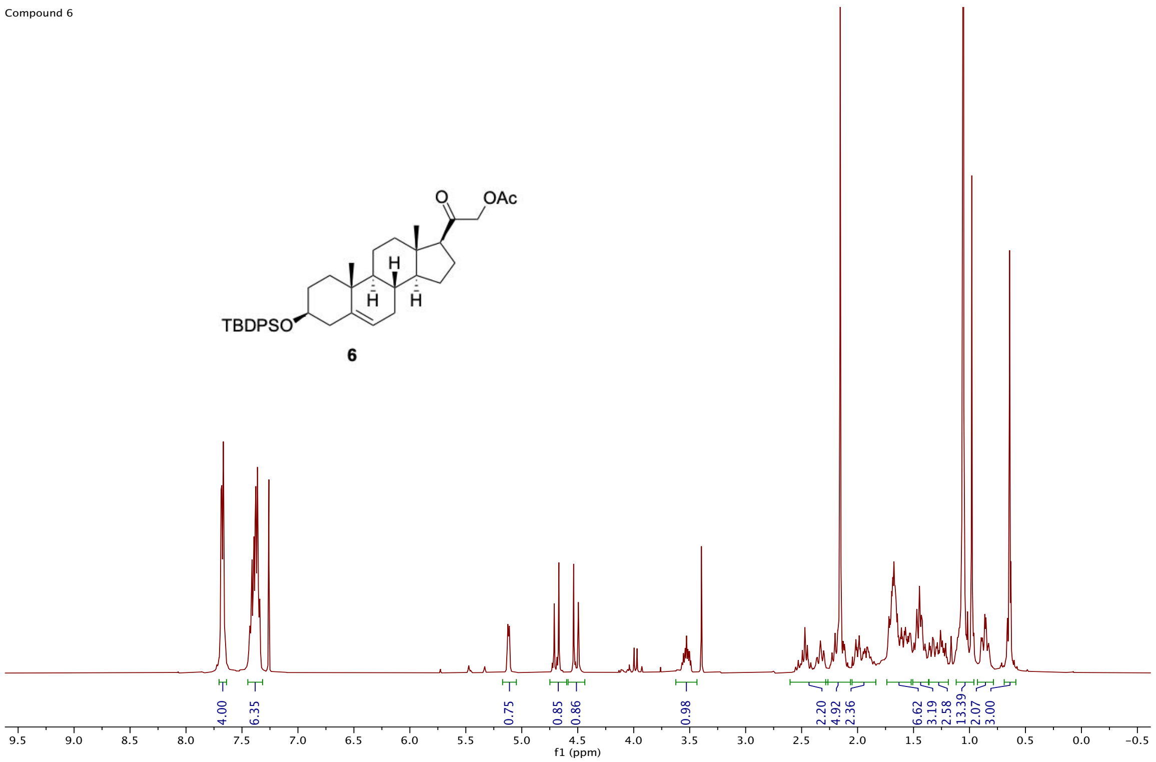

Compound 6

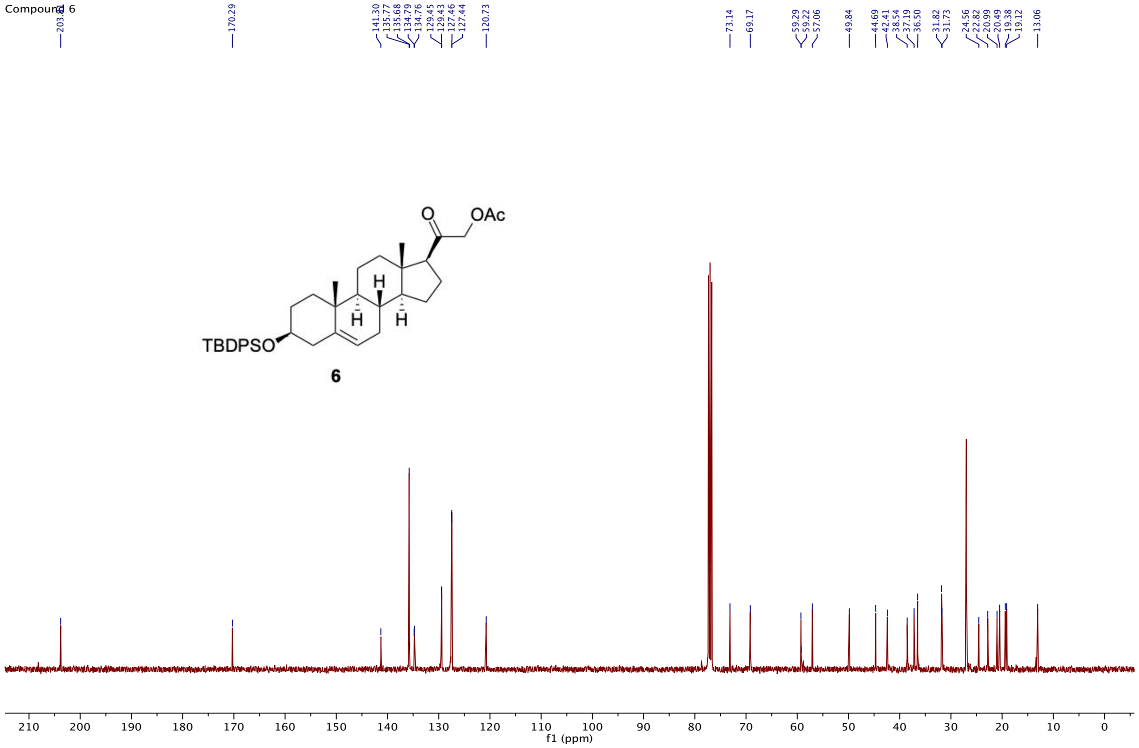

Compound 7

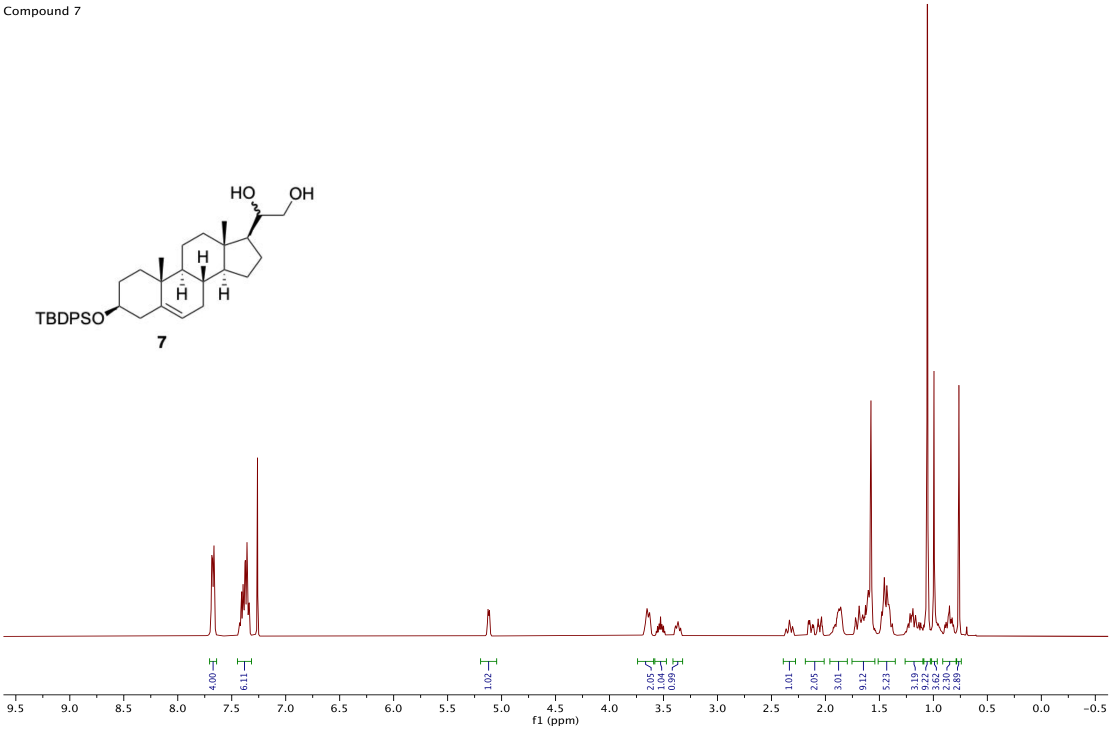

Compound 7

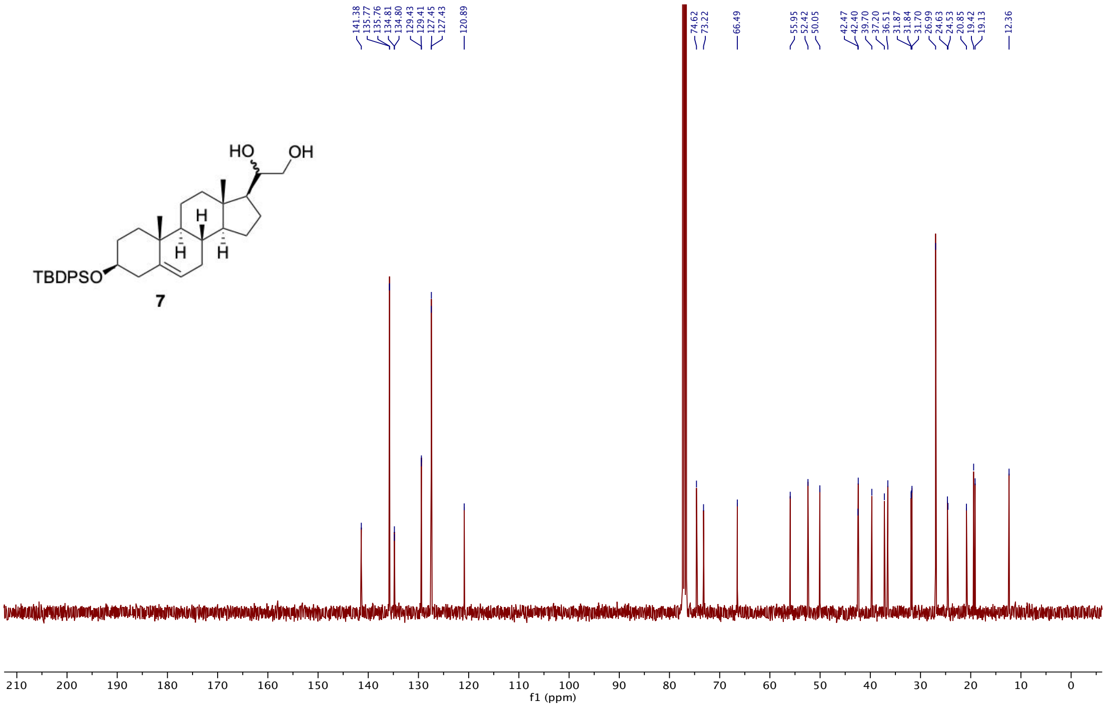

Compound 8

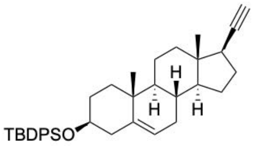
TBDPSO
H
H
H

8

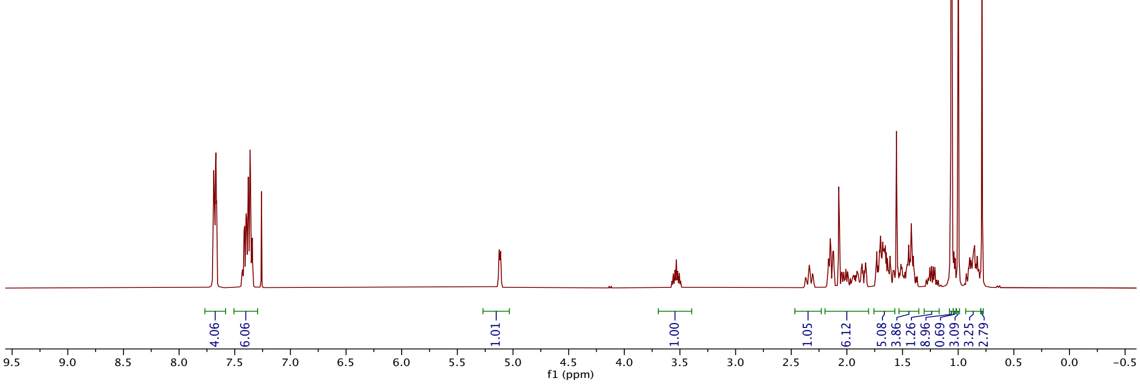
4.06
6.06
1.01
1.00
1.05
6.12
5.08
3.86
1.26
8.96
0.69
3.09
3.25
2.79
9.5
9.0
8.5
8.0
7.5
7.0
6.5
6.0
5.5
5.0
4.5
4.0
3.5
3.0
2.5
2.0
1.5
1.0
0.5
0.0
-0.5
f1 (ppm)

Compound 9

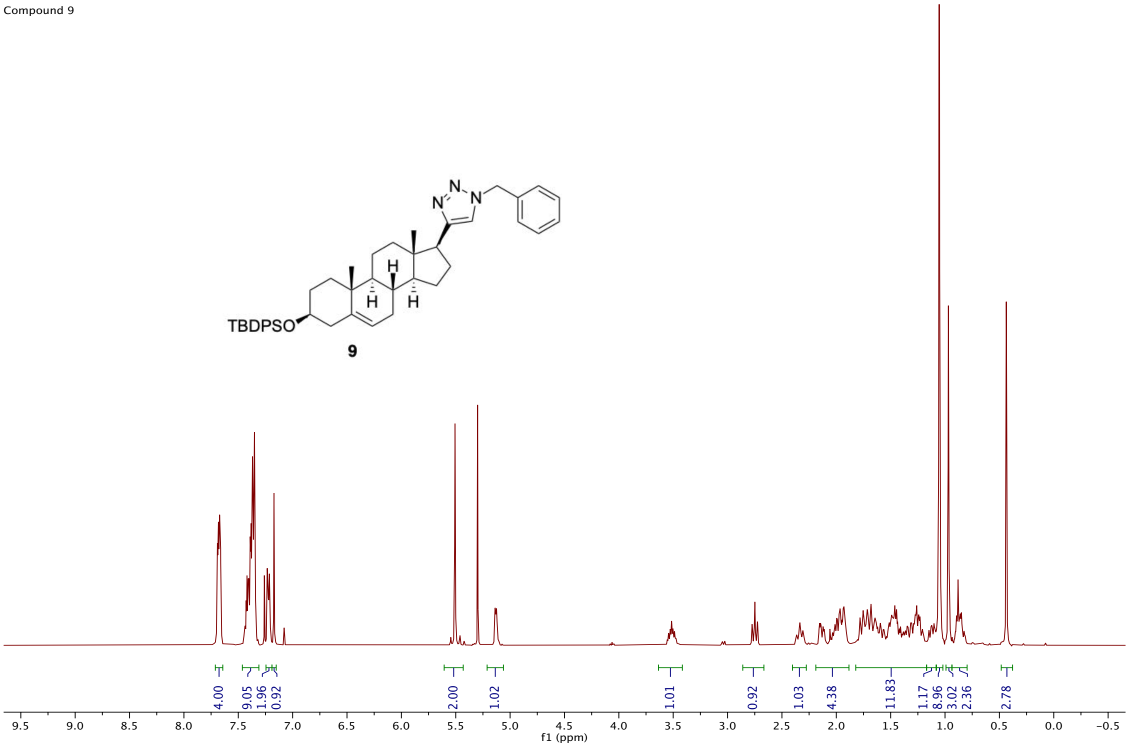

Compound 9

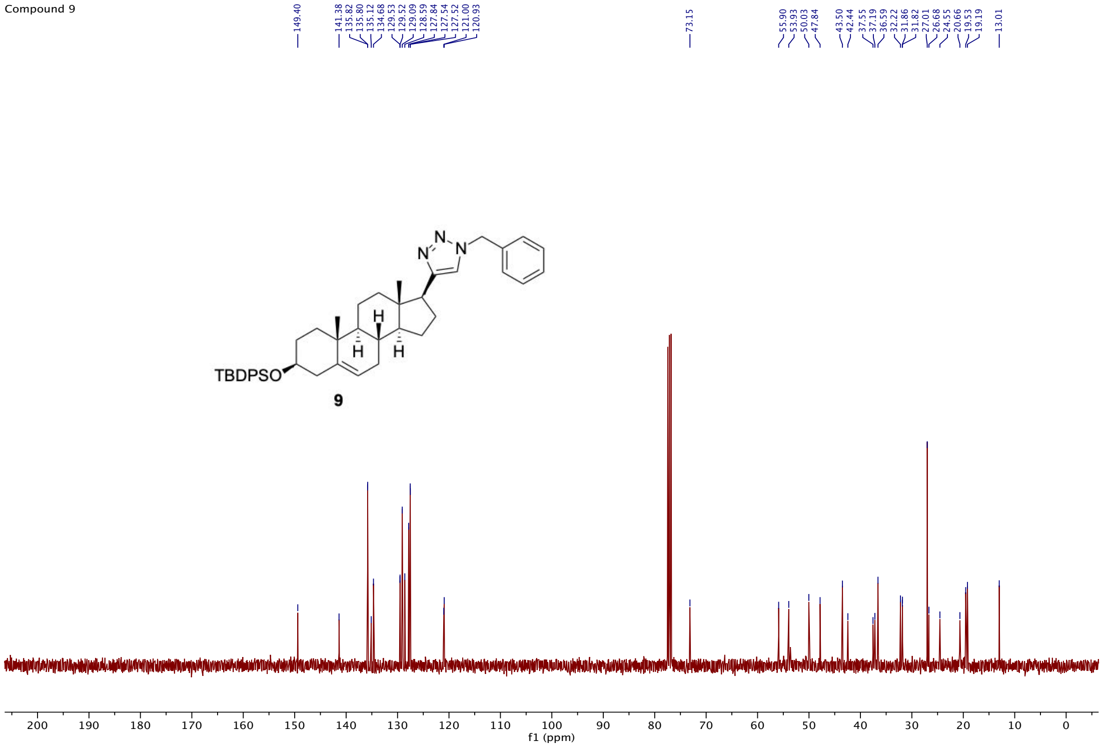

Single Mass Analysis

Tolerance = 1000.0 PPM / DBE: min = 0.0, max = 50.0
Element prediction: Off
Number of isotope peaks used for i-FIT = 3

Monoisotopic Mass, Even Electron Ions
318 formula(e) evaluated with 213 results within limits (up to 5 closest results for each mass)
Elements Used:
C: 5-50 H: 3-72 N: 1-3 O: 1-9 Si: 1-2

Compound 9 20 (0.369) AM (Cen,4, 50.00, Ar,6000.0,686.85,0.80,LS 10); Cm (15:22)
1: TOF MS ES+

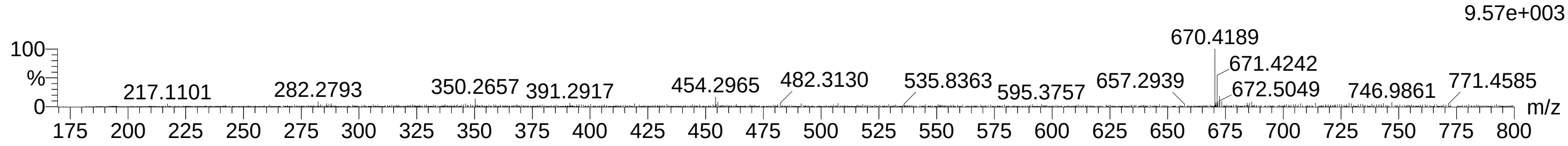

Minimum:				0.0		
Maximum:		5.0	1000.0	50.0		
Mass	Calc. Mass	mDa	PPM	DBE	i-FIT	Formula
670.4189	670.4193	-0.4	-0.6	19.5	3.0	C44 H56 N3 O Si
	670.4224	-3.5	-5.2	14.5	45.1	C40 H60 N3 O2 Si2
	670.4139	5.0	7.5	10.5	23.9	C38 H60 N O7 Si
	670.4251	-6.2	-9.2	10.5	27.9	C37 H60 N3 O6 Si
	670.4171	1.8	2.7	5.5	29.7	C34 H64 N O8 Si2

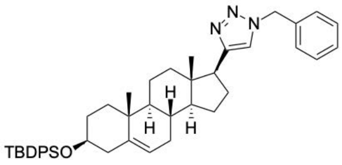

9

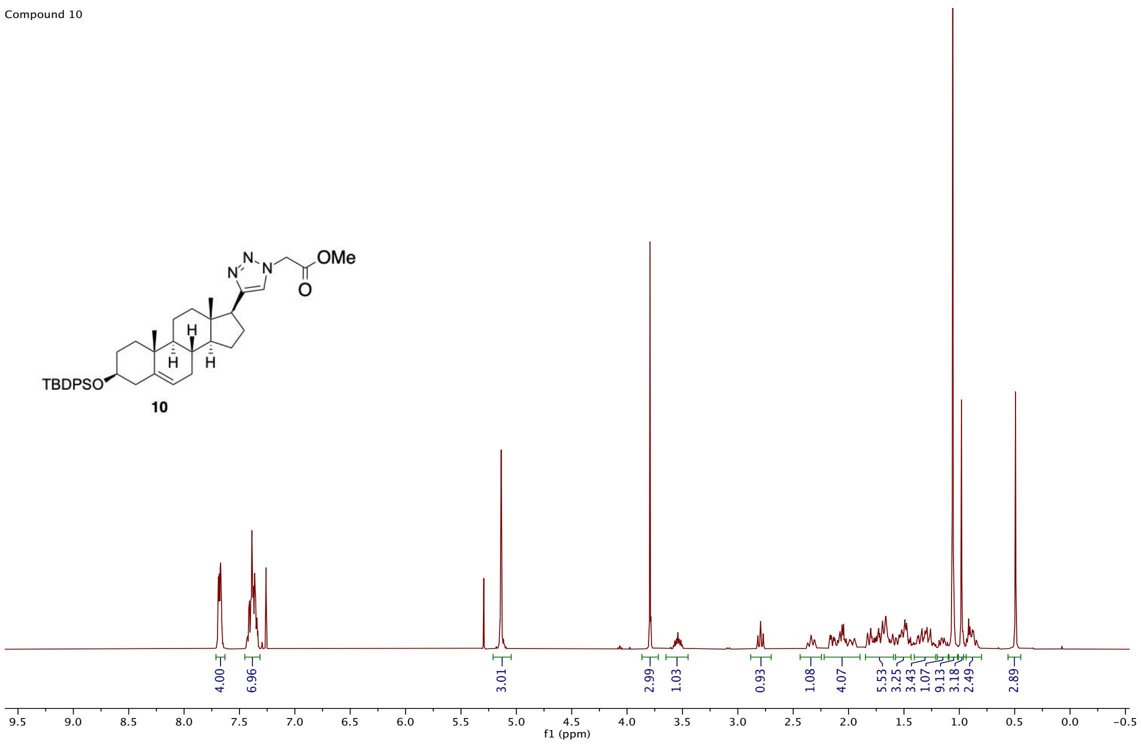

Compound 10
OMe
TBDPSO
10
4.00
6.96
3.01
2.99
1.03
0.93
1.08
4.07
5.53
3.25
3.43
1.07
9.13
3.18
2.49
2.89
9.5 9.0 8.5 8.0 7.5 7.0 6.5 6.0 5.5 5.0 4.5 4.0 3.5 3.0 2.5 2.0 1.5 1.0 0.5 0.0 −0.5
f1 (ppm)

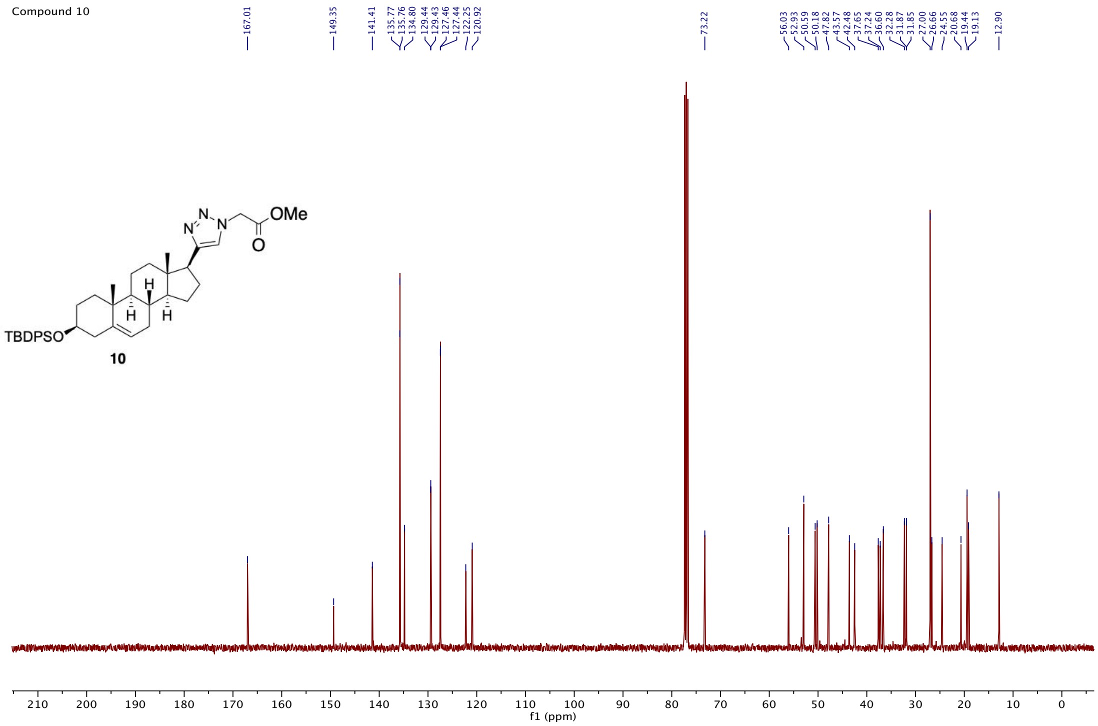

Compound 10
167.01
149.35
141.41
135.77
135.76
134.80
129.44
129.43
127.46
127.44
122.25
120.92
73.22
56.03
52.93
50.59
50.18
47.82
43.57
42.48
37.65
37.24
36.60
32.28
31.87
31.85
27.00
26.66
24.55
20.68
19.44
19.13
12.90
OMe
TBDPSO
10
210 200 190 180 170 160 150 140 130 120 110 100 90 80 70 60 50 40 30 20 10 0
f1 (ppm)

Single Mass Analysis

Tolerance = 1000.0 PPM / DBE: min = 0.0, max = 50.0
Element prediction: Off
Number of isotope peaks used for i-FIT = 3

Monoisotopic Mass, Even Electron Ions
314 formula(e) evaluated with 153 results within limits (up to 5 closest results for each mass)
Elements Used:
C: 5-50 H: 3-72 N: 1-6 O: 1-9 Si: 1-1

Compound 10 19 (0.352) AM (Cen,4, 50.00, Ar,6000.0,588.87,0.80,LS 10); Cm (13:26)
1: TOF MS ES+

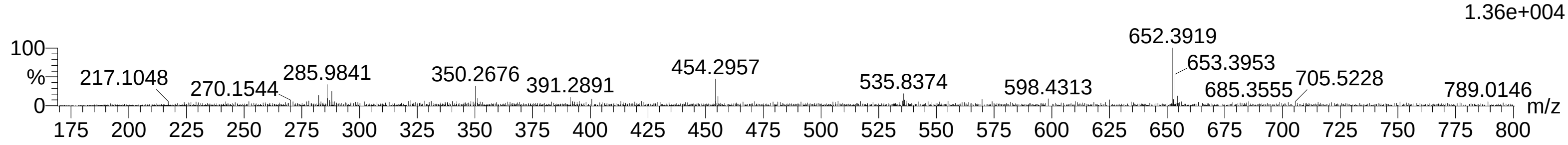

Minimum:				0.0		
Maximum:		5.0	1000.0	50.0		
Mass	Calc. Mass	mDa	PPM	DBE	i-FIT	Formula
652.3919	652.3934	-1.5	-2.3	16.5	6.8	C40 H54 N3 O3 Si
	652.3894	2.5	3.8	12.5	53.9	C35 H54 N5 O5 Si
	652.3881	3.8	5.8	7.5	94.5	C34 H58 N O9 Si
	652.3975	-5.6	-8.6	20.5	22.9	C45 H54 N O Si
	652.3993	-7.4	-11.3	7.5	110.8	C33 H58 N3 O8 Si

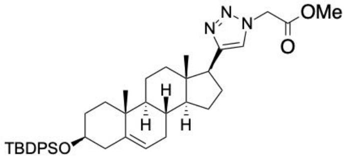

10

Compound 12

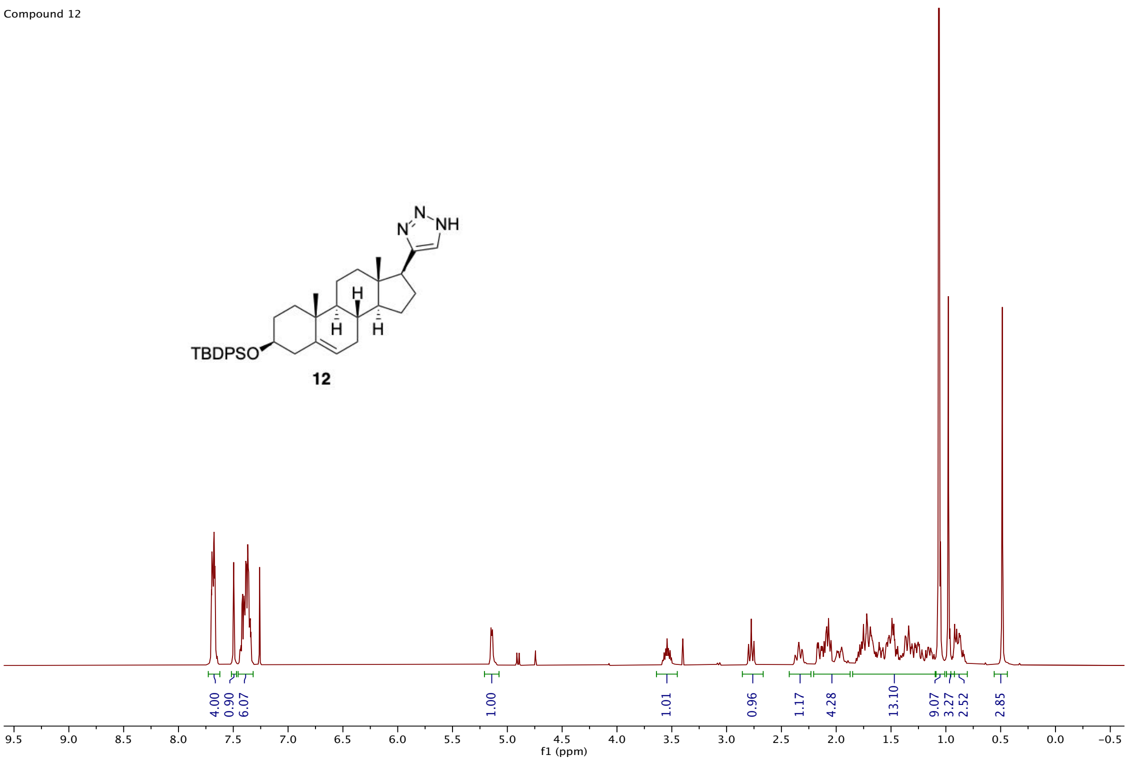

TBDPSO
12
4.00
0.90
6.07
1.00
1.01
0.96
1.17
4.28
13.10
9.07
3.27
2.52
2.85
f1 (ppm)

Single Mass Analysis

Tolerance = 1000.0 PPM / DBE: min = 0.0, max = 50.0
Element prediction: Off
Number of isotope peaks used for i-FIT = 3

Monoisotopic Mass, Even Electron Ions
301 formula(e) evaluated with 141 results within limits (up to 5 closest results for each mass)
Elements Used:
C: 5-50 H: 3-72 N: 1-6 O: 1-9 Si: 1-1

Compound 12 19 (0.352) AM (Cen,4, 50.00, Ar,6000.0,490.89,0.80,LS 10); Cm (15:25)
1: TOF MS ES+

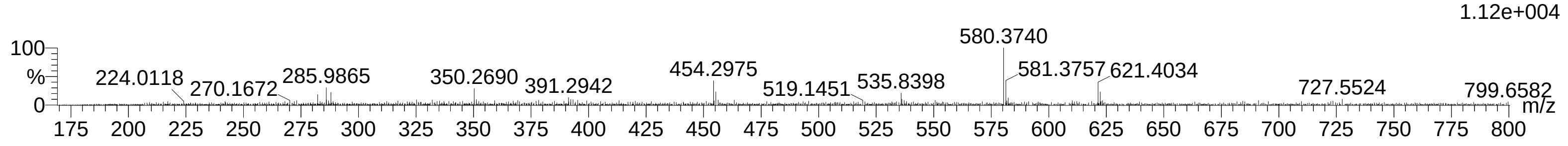

Minimum:				0.0		
Maximum:		5.0	1000.0	50.0		
Mass	Calc. Mass	mDa	PPM	DBE	i-FIT	Formula
580.3740	580.3742	-0.2	-0.3	2.5	50.1	C25 H54 N5 O8 Si
	580.3723	1.7	2.9	15.5	41.6	C37 H50 N3 O Si
	580.3782	-4.2	-7.2	6.5	8.2	C30 H54 N3 O6 Si
	580.3683	5.7	9.8	11.5	7.9	C32 H50 N5 O3 Si
	580.3670	7.0	12.1	6.5	13.0	C31 H54 N O7 Si

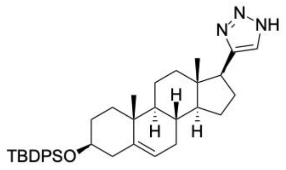

12

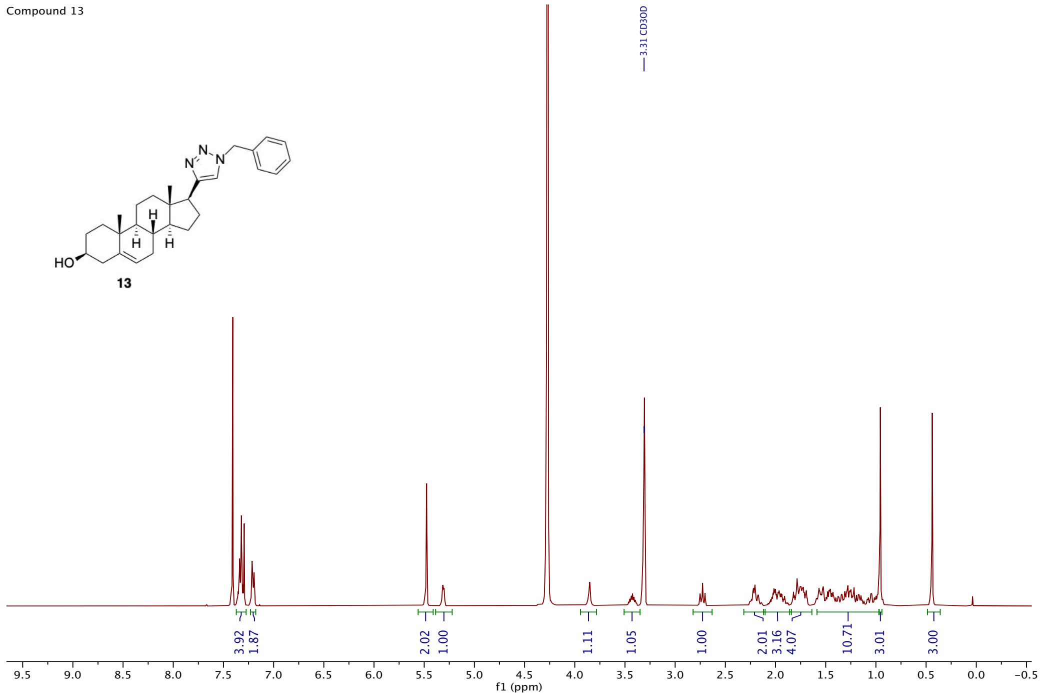

Compound 13
3.31 CD3OD
HO
13
3.92
1.87
2.02
1.00
1.11
1.05
1.00
2.01
3.16
4.07
10.71
3.01
3.00
f1 (ppm)

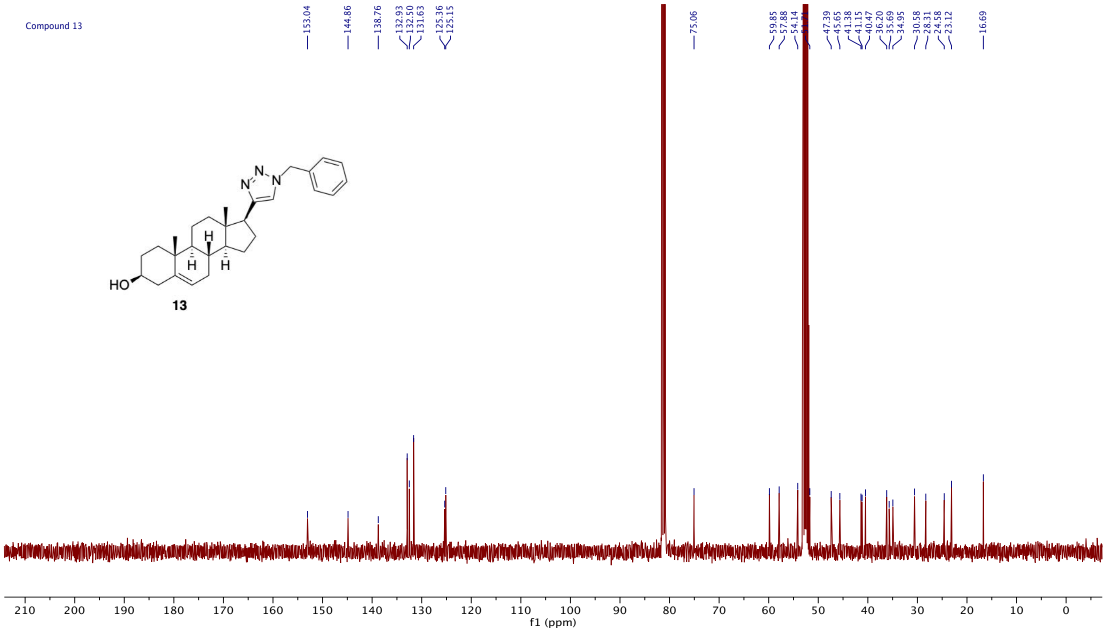

Compound 13
153.04
144.86
138.76
132.93
132.50
131.63
125.36
125.15
75.06
59.85
57.88
54.14
47.39
45.65
41.38
41.15
40.47
36.20
35.69
34.95
30.58
28.31
24.58
23.12
16.69
HO
13
f1 (ppm)

Single Mass Analysis
Tolerance = 1000.0 PPM / DBE: min = 0.0, max = 50.0
Element prediction: Off
Number of isotope peaks used for i-FIT = 3

Monoisotopic Mass, Even Electron Ions
234 formula(e) evaluated with 99 results within limits (up to 5 closest results for each mass)
Elements Used:
C: 5-50 H: 3-72 N: 1-6 O: 1-9

Compound 13 19 (0.352) AM (Cen,4, 50.00, Ar,6000.0,490.89,0.80,LS 10); Cm (15:25)
1: TOF MS ES+

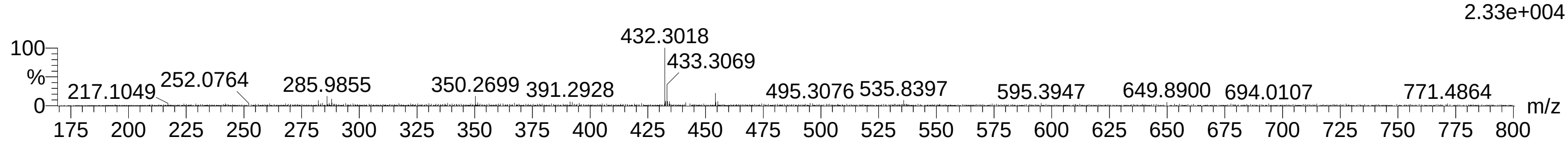

Minimum:				0.0		
Maximum:		5.0	1000.0	50.0		
Mass	Calc. Mass	mDa	PPM	DBE	i-FIT	Formula
432.3018	432.3015	0.3	0.7	11.5	67.1	C28 H38 N3 O
	432.2975	4.3	9.9	7.5	284.2	C23 H38 N5 O3
	432.3074	-5.6	-13.0	2.5	437.4	C21 H42 N3 O6
	432.2961	5.7	13.2	2.5	398.3	C22 H42 N O7
	432.3114	-9.6	-22.2	6.5	182.1	C26 H42 N O4

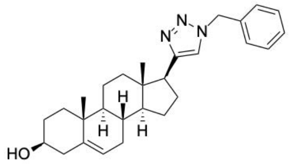

13

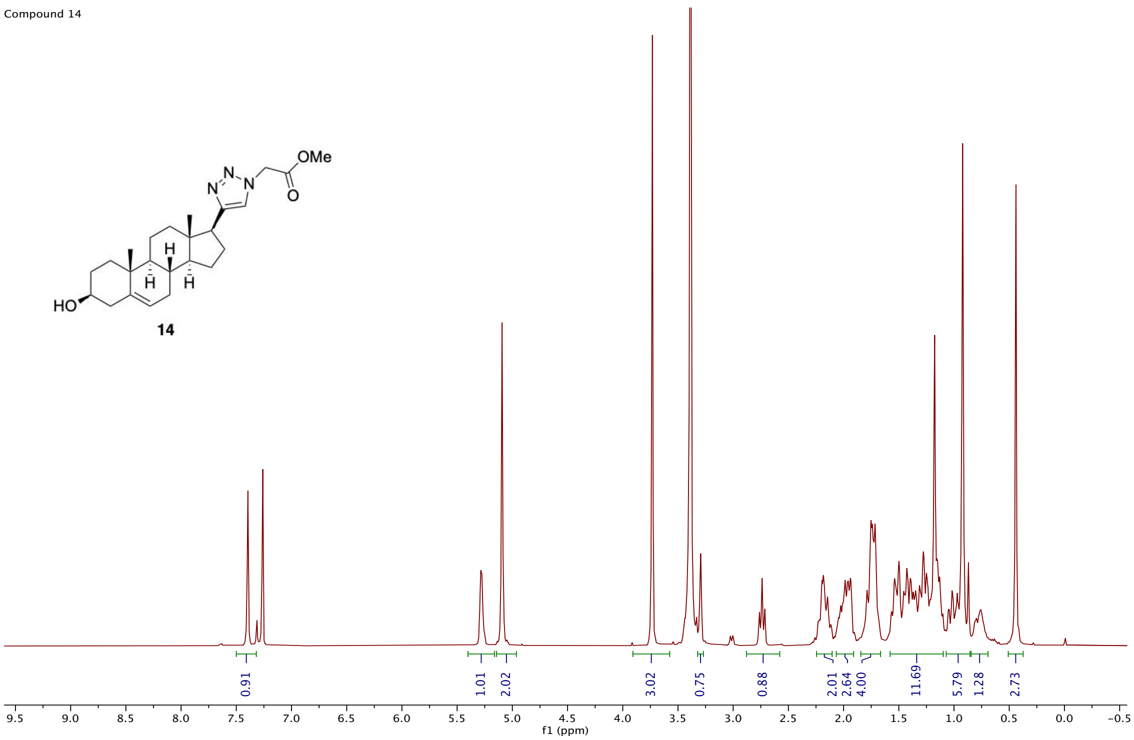
Compound 14
OMe
O
N
N
N
H
H
H
HO
14
0.91
1.01
2.02
3.02
0.75
0.88
2.01
2.64
4.00
11.69
5.79
1.28
2.73
9.5 9.0 8.5 8.0 7.5 7.0 6.5 6.0 5.5 5.0 4.5 4.0 3.5 3.0 2.5 2.0 1.5 1.0 0.5 0.0 -0.5
f1 (ppm)

Single Mass Analysis

Tolerance = 1000.0 PPM / DBE: min = 0.0, max = 50.0
Element prediction: Off
Number of isotope peaks used for i-FIT = 3

Monoisotopic Mass, Even Electron Ions
219 formula(e) evaluated with 99 results within limits (up to 5 closest results for each mass)
Elements Used:
C: 5-50 H: 3-72 N: 1-6 O: 1-9

Compound 14 19 (0.352) AM (Cen,4, 50.00, Ar,6000.0,490.89,0.80,LS 10); Cm (12:27)
1: TOF MS ES+

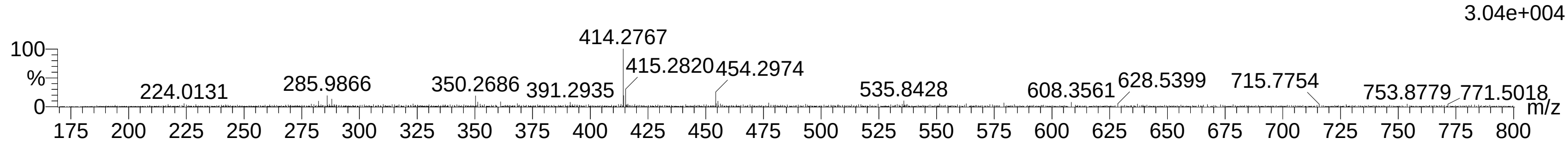

Minimum:				0.0		
Maximum:		5.0	1000.0	50.0		
Mass	Calc. Mass	mDa	PPM	DBE	i-FIT	Formula
414.2767	414.2757	1.0	2.4	8.5	13.8	C24 H36 N3 O3
	414.2797	-3.0	-7.2	12.5	61.9	C29 H36 N O
	414.2716	5.1	12.3	4.5	215.6	C19 H36 N5 O5
	414.2856	-8.9	-21.5	3.5	129.8	C22 H40 N O6
	414.2869	-10.2	-24.6	8.5	82.0	C23 H36 N5 O2

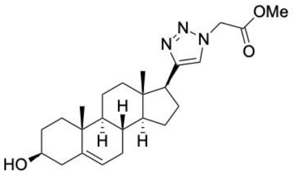

14

Compound 16

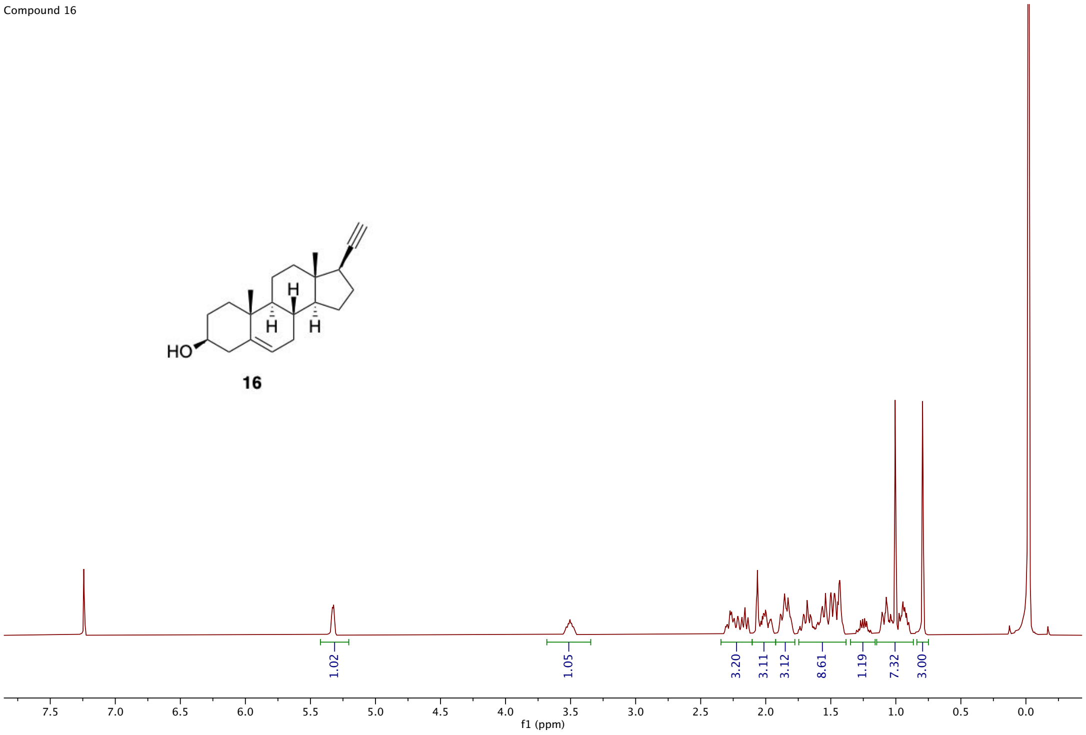

Compound 16

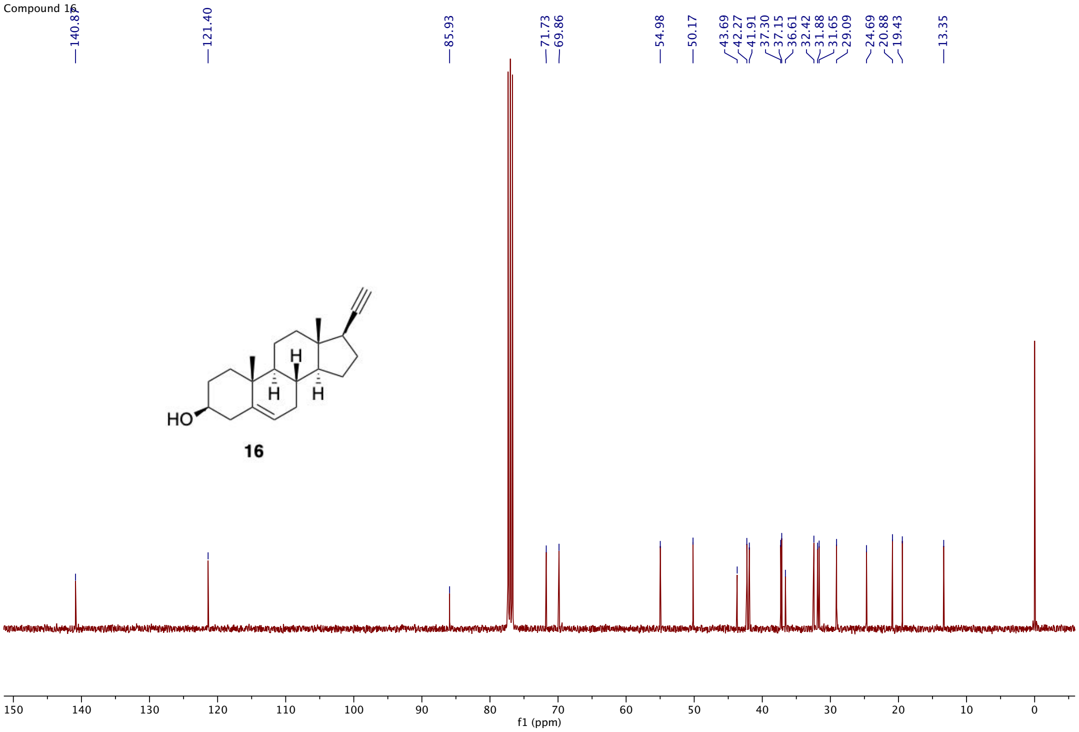

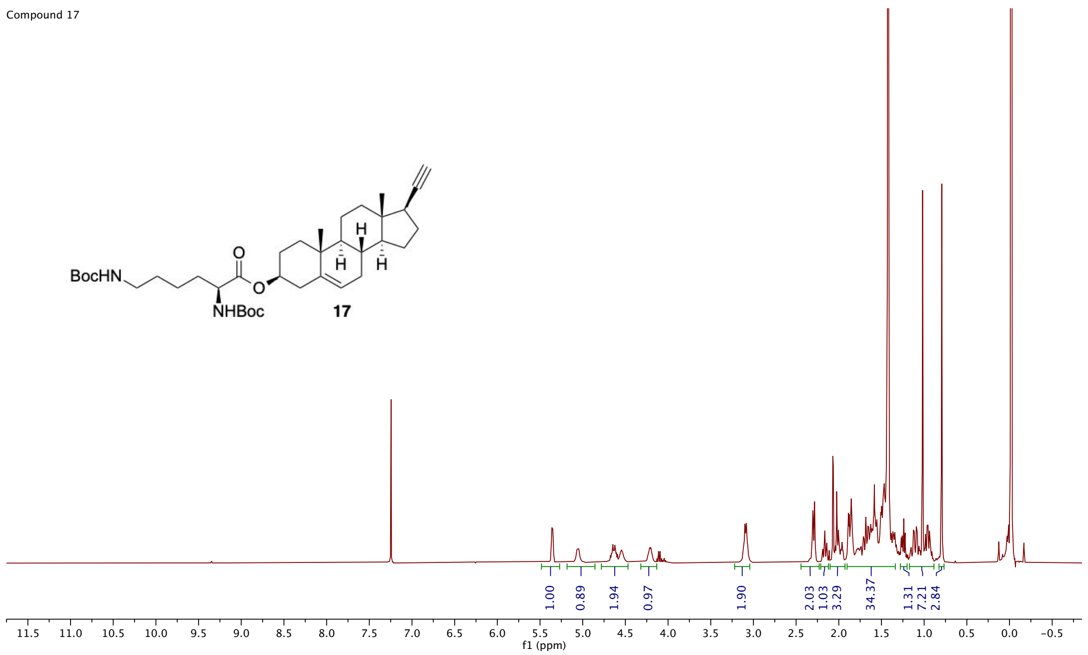
Compound 17
BocHN
NHBoc
17
1.00
0.89
1.94
0.97
1.90
2.03
1.03
3.29
34.37
1.31
7.21
2.84
f1 (ppm)

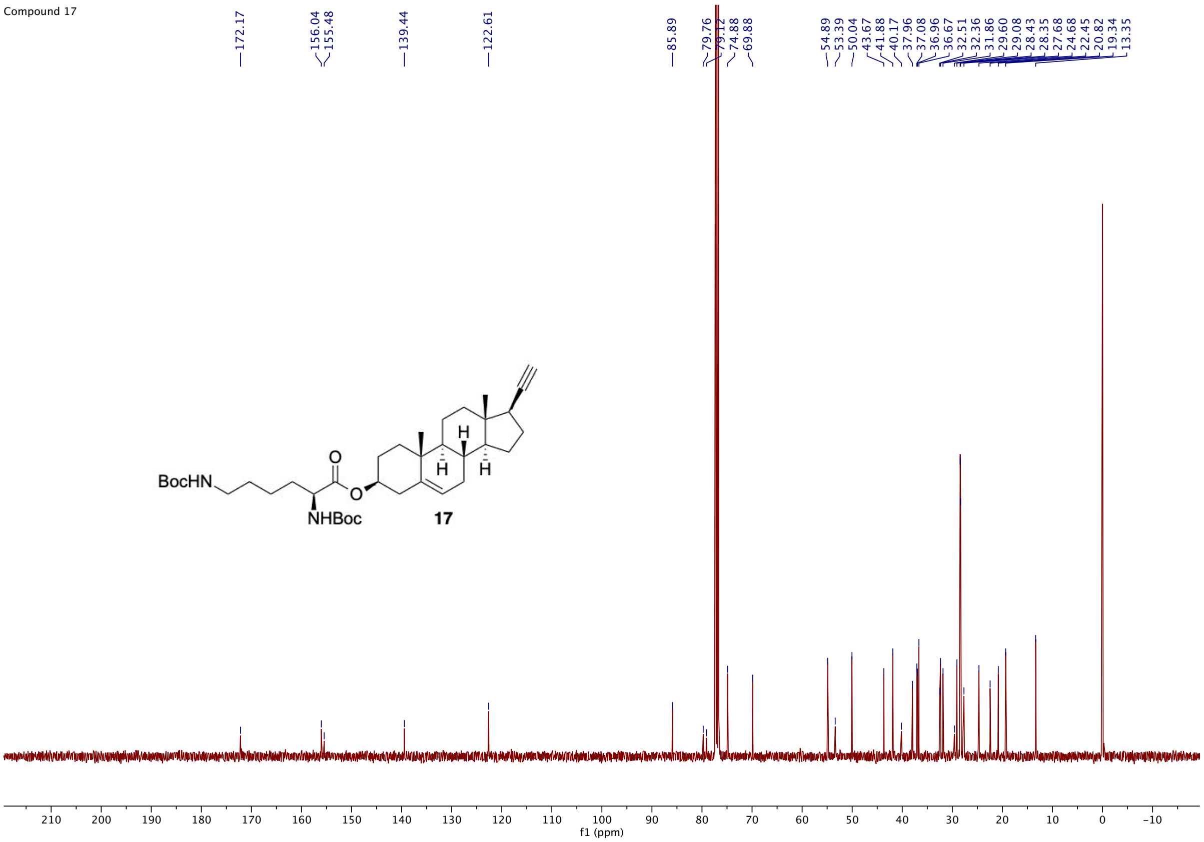
Compound 17
172.17
156.04
155.48
139.44
122.61
85.89
79.76
79.12
74.88
69.88
54.89
53.39
50.04
43.67
41.88
40.17
37.96
37.08
36.96
36.67
32.51
32.36
31.86
29.60
29.08
28.43
28.35
27.68
24.68
22.45
20.82
19.34
13.35
BocHN
NHBoc
17
f1 (ppm)

Compound 18

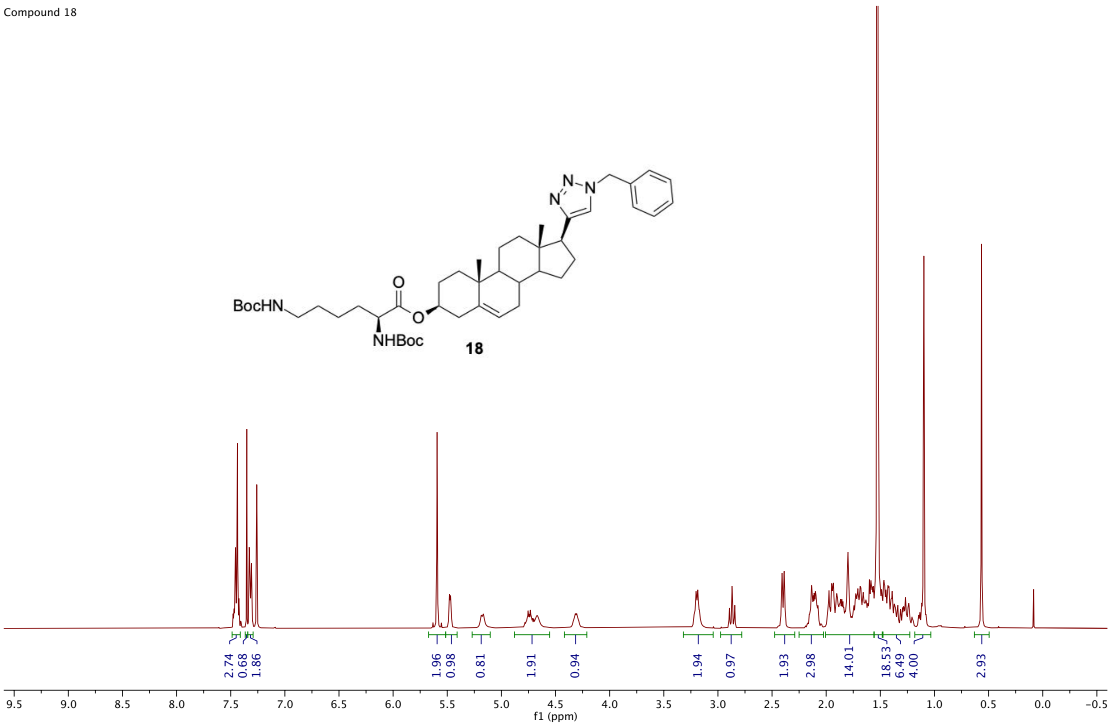

Compound 18

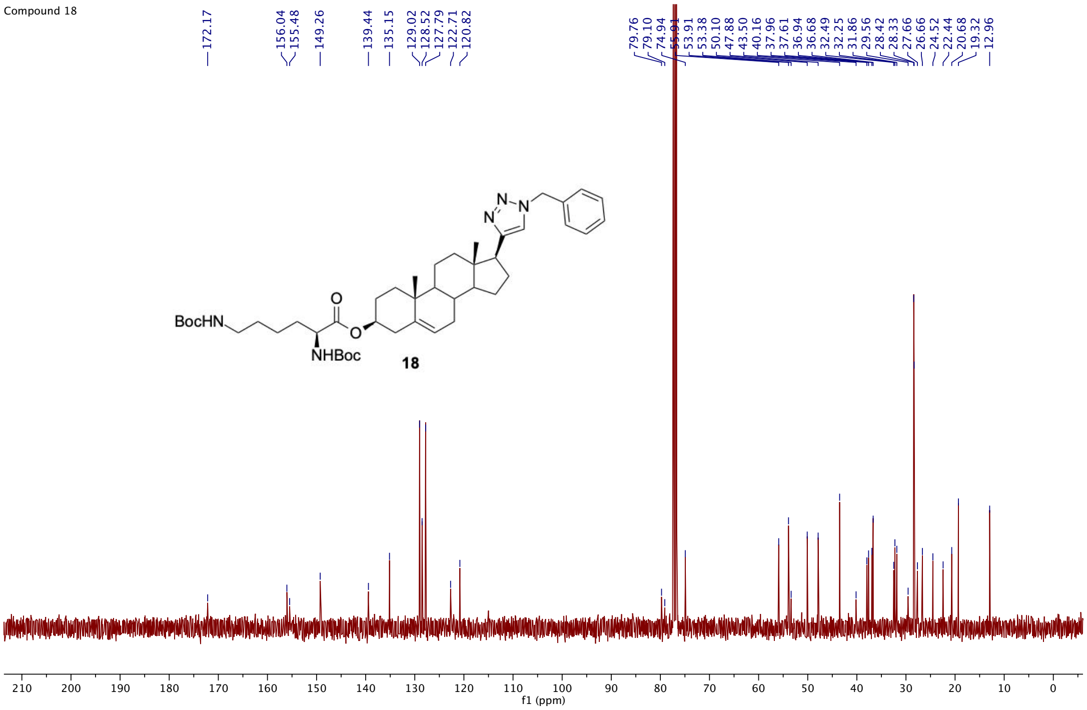

Compound 19

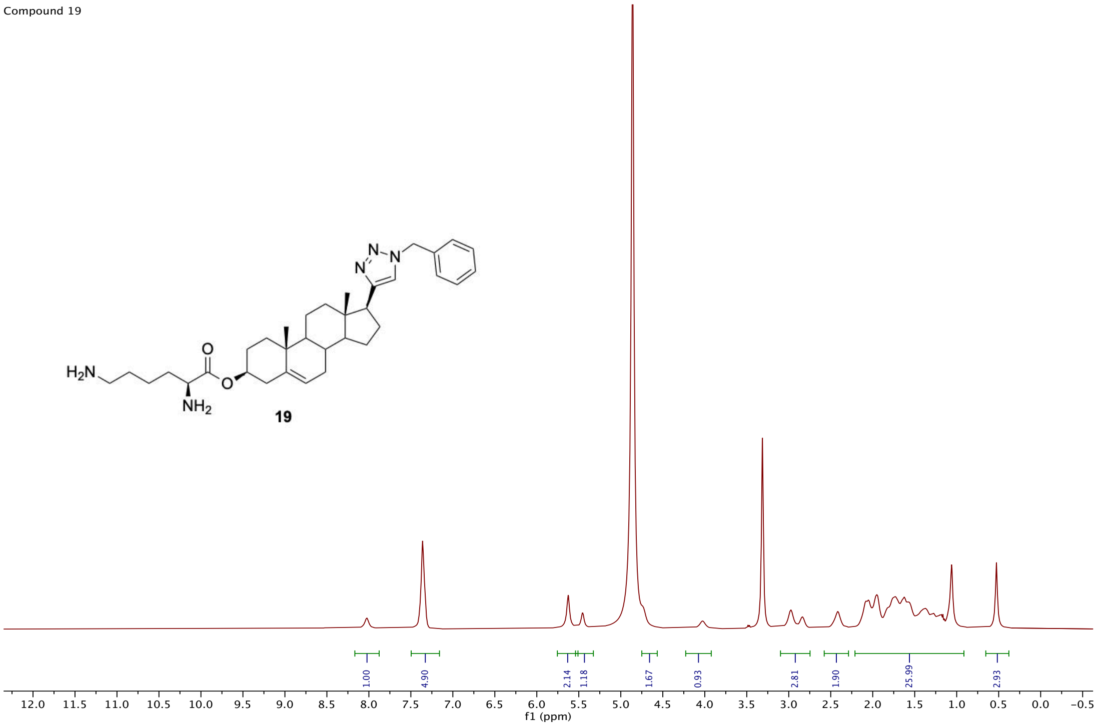
H2N
NH2
O
O
N
N
N
19
1.00
4.90
2.14
1.18
1.67
0.93
2.81
1.90
25.99
2.93
12.0 11.5 11.0 10.5 10.0 9.5 9.0 8.5 8.0 7.5 7.0 6.5 6.0 5.5 5.0 4.5 4.0 3.5 3.0 2.5 2.0 1.5 1.0 0.5 0.0 −0.5
f1 (ppm)

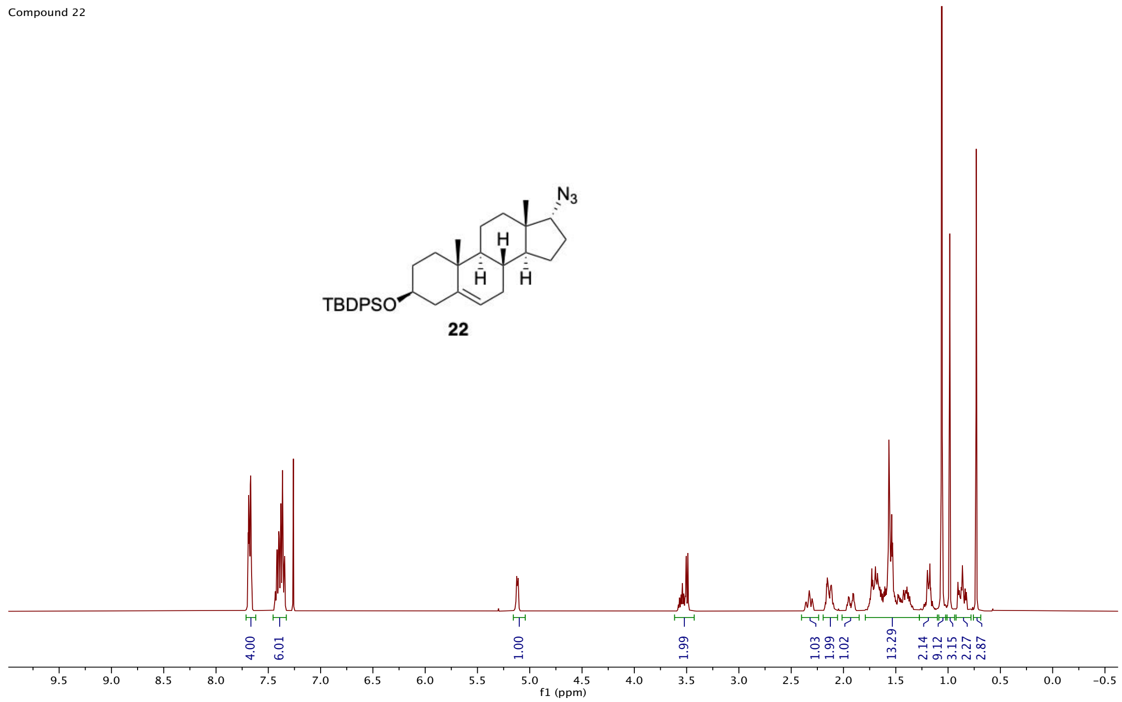
Compound 22
N_3
H
H
H
TBDPSO
22
4.00
6.01
1.00
1.99
1.03
1.99
1.02
13.29
2.14
9.12
3.15
2.27
2.87
9.5 9.0 8.5 8.0 7.5 7.0 6.5 6.0 5.5 5.0 4.5 4.0 3.5 3.0 2.5 2.0 1.5 1.0 0.5 0.0 −0.5
f1 (ppm)

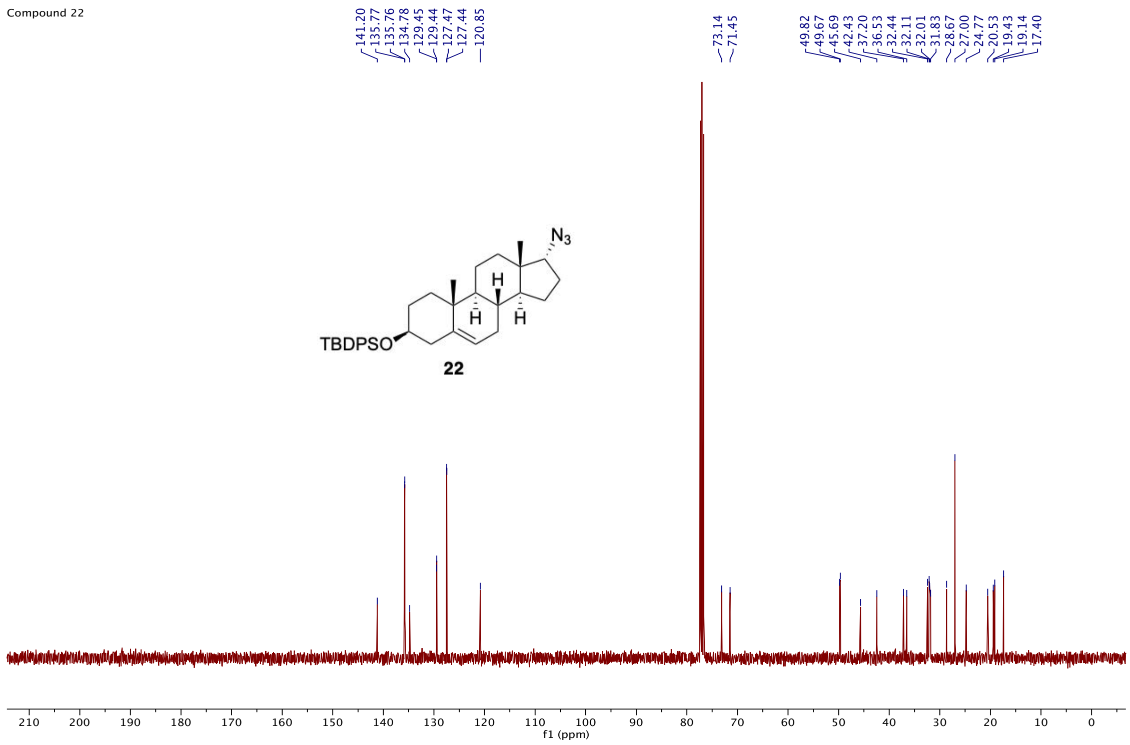
Compound 22
141.20
135.77
135.76
134.78
129.45
129.44
127.47
127.44
120.85
73.14
71.45
49.82
49.67
45.69
42.43
37.20
36.53
32.44
32.11
32.01
31.83
28.67
27.00
24.77
20.53
19.43
19.14
17.40
N_3
H
H
H
TBDPSO
22
210 200 190 180 170 160 150 140 130 120 110 100 90 80 70 60 50 40 30 20 10 0
f1 (ppm)

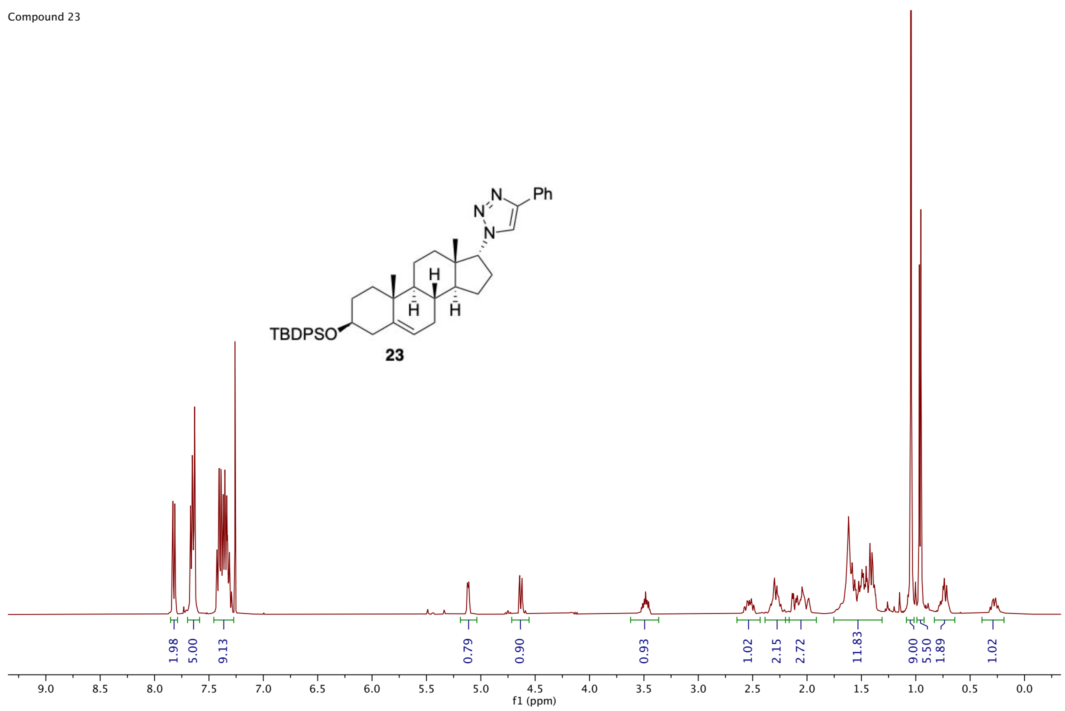

Compound 23
N
N
N
Ph
H
H
H
TBDPSO
23
1.98
5.00
9.13
0.79
0.90
0.93
1.02
2.15
2.72
11.83
9.00
5.50
1.89
1.02
9.0 8.5 8.0 7.5 7.0 6.5 6.0 5.5 5.0 4.5 4.0 3.5 3.0 2.5 2.0 1.5 1.0 0.5 0.0
f1 (ppm)

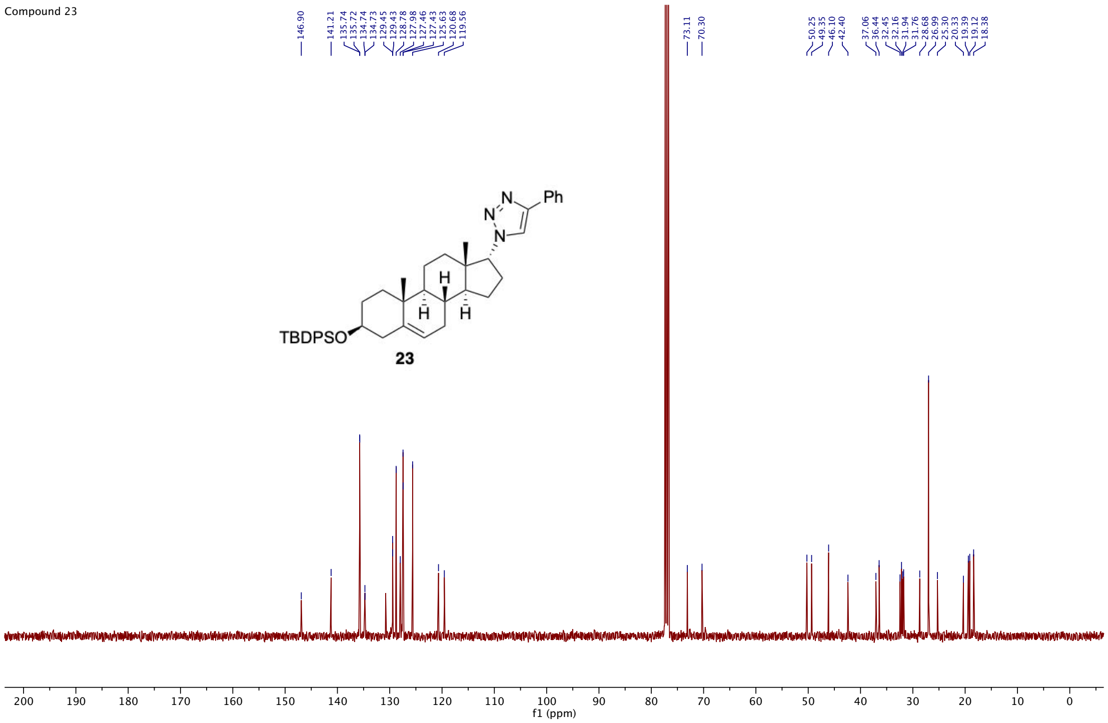

Compound 23
146.90
141.21
135.74
135.72
134.74
134.73
129.45
129.43
128.78
127.98
127.46
127.43
125.63
120.68
119.56
73.11
70.30
50.25
49.35
46.10
42.40
37.06
36.44
32.45
32.16
31.94
31.76
28.68
26.99
25.30
20.33
19.39
19.12
18.38
N
N
N
Ph
H
H
H
TBDPSO
23
200 190 180 170 160 150 140 130 120 110 100 90 80 70 60 50 40 30 20 10 0
f1 (ppm)

Single Mass Analysis

Tolerance = 1000.0 PPM / DBE: min = 0.0, max = 50.0
Element prediction: Off
Number of isotope peaks used for i-FIT = 3

Monoisotopic Mass, Even Electron Ions
312 formula(e) evaluated with 201 results within limits (up to 5 closest results for each mass)
Elements Used:
C: 5-50 H: 3-72 N: 1-3 O: 1-9 Si: 1-2
Compound 23 19 (0.352) AM (Cen,4, 50.00, Ar,6000.0,686.85,0.80,LS 10); Cm (15:22)
1: TOF MS ES+

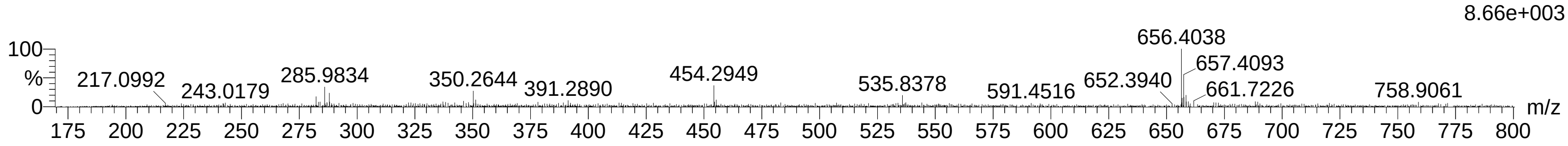

Minimum:				0.0		
Maximum:		5.0	1000.0	50.0		
Mass	Calc. Mass	mDa	PPM	DBE	i-FIT	Formula
656.4038	656.4036	0.2	0.3	19.5	4.5	C43 H54 N3 O Si
	656.4014	2.4	3.7	5.5	27.1	C33 H62 N O8 Si2
	656.4068	-3.0	-4.6	14.5	11.3	C39 H58 N3 O2 Si2
	656.3983	5.5	8.4	10.5	54.4	C37 H58 N O7 Si
	656.4095	-5.7	-8.7	10.5	62.8	C36 H58 N3 O6 Si

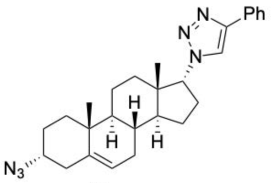

25

Compound 24

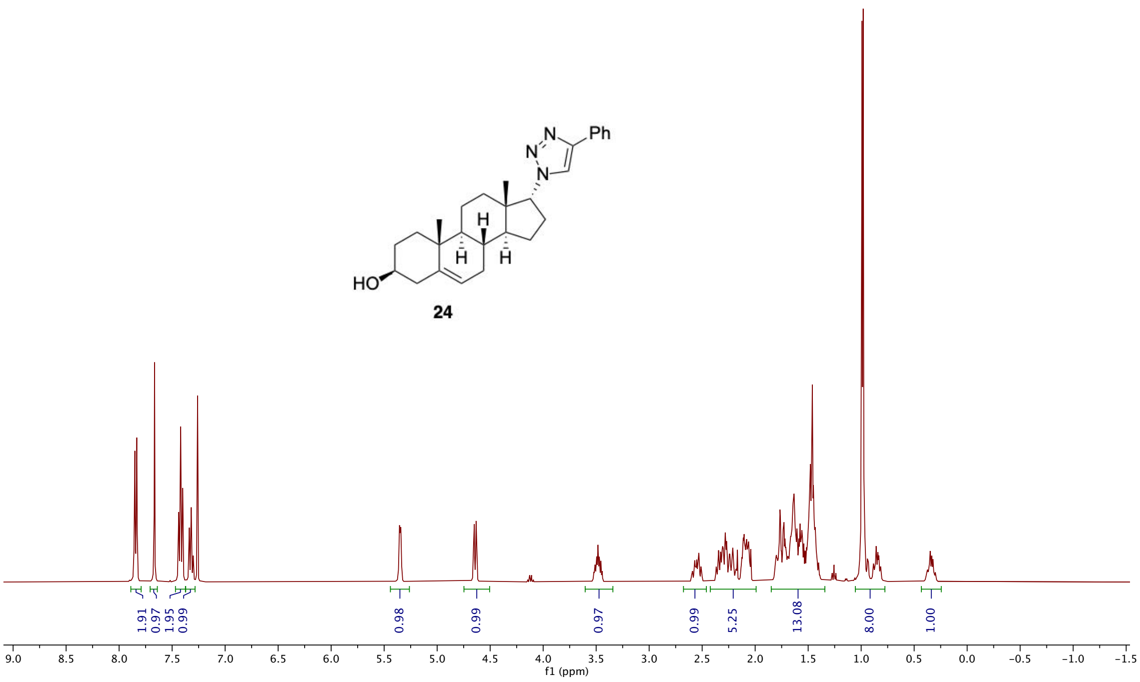

Compound 24

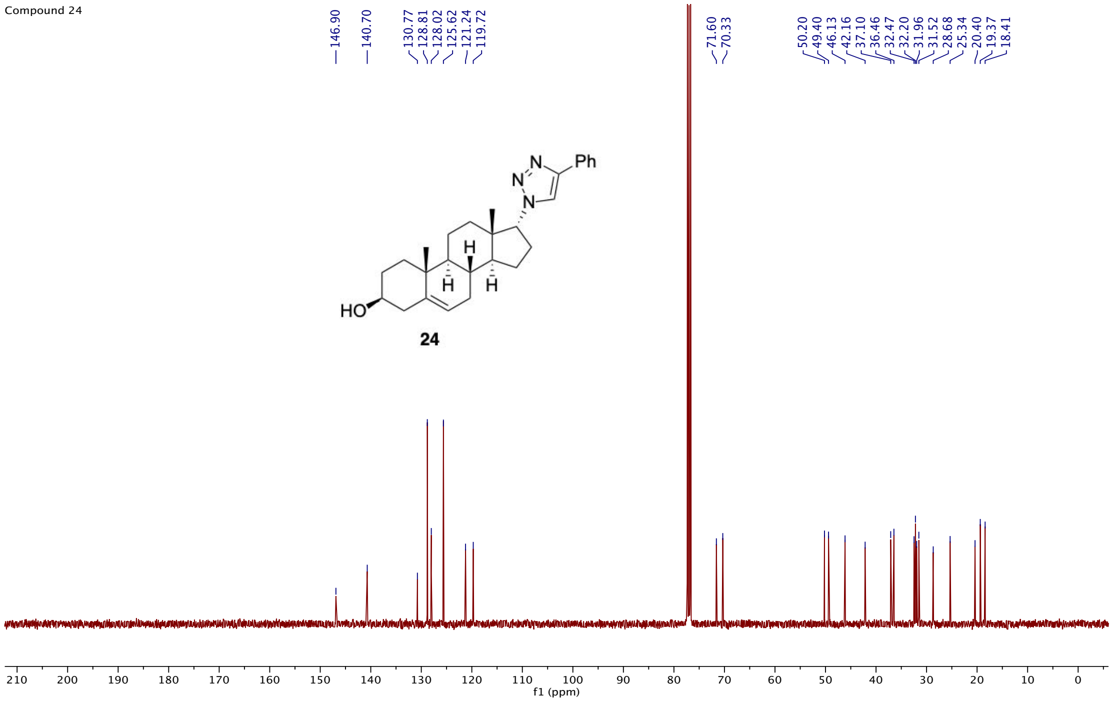

Single Mass Analysis

Tolerance = 1000.0 PPM / DBE: min = 0.0, max = 50.0
Element prediction: Off
Number of isotope peaks used for i-FIT = 3

Monoisotopic Mass, Even Electron Ions
118 formula(e) evaluated with 68 results within limits (up to 5 closest results for each mass)
Elements Used:
C: 5-50 H: 3-72 N: 1-3 O: 1-9

Compound 24 18 (0.335) AM (Cen,4, 50.00, Ar,6000.0,490.89,0.80,LS 10); Cm (15:21)
1: TOF MS ES+

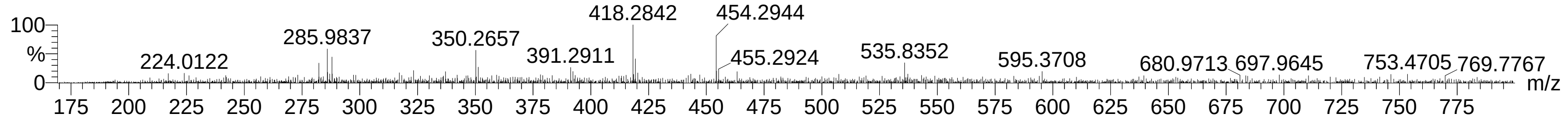

Minimum:				0.0		
Maximum:		5.0	1000.0	50.0		

Mass	Calc. Mass	mDa	PPM	DBE	i-FIT	Formula
418.2842	418.2858	-1.6	-3.8	11.5	165.5	C27 H36 N3 O
	418.2805	3.7	8.8	2.5	255.7	C21 H40 N O7
	418.2917	-7.5	-17.9	2.5	272.4	C20 H40 N3 O6
	418.2746	9.6	23.0	11.5	163.3	C28 H36 N O2
	418.2957	-11.5	-27.5	6.5	203.1	C25 H40 N O4

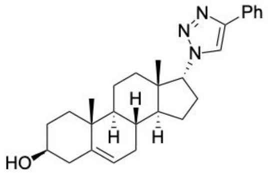

24

Compound 25

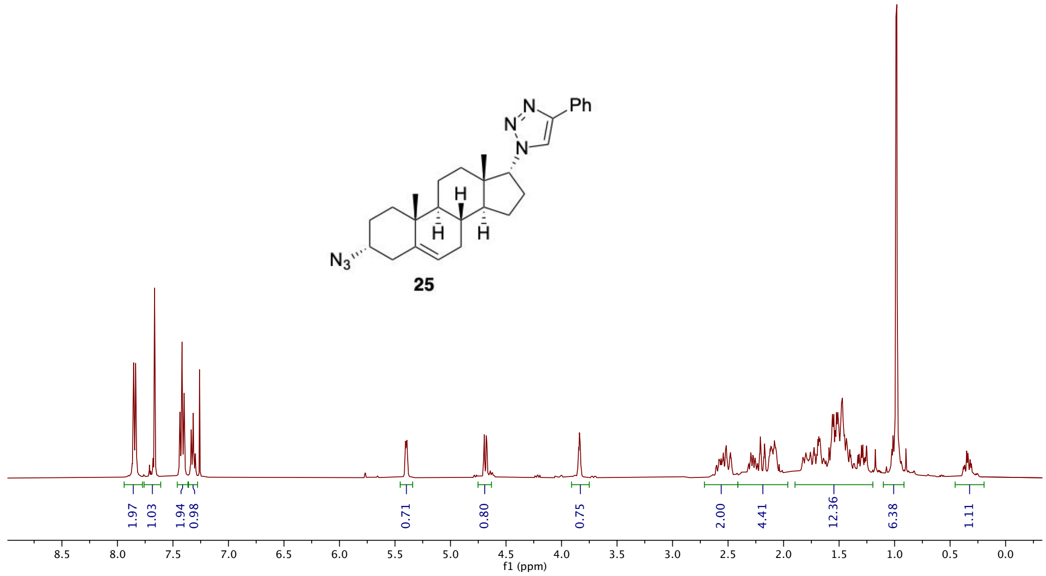

Compound 25

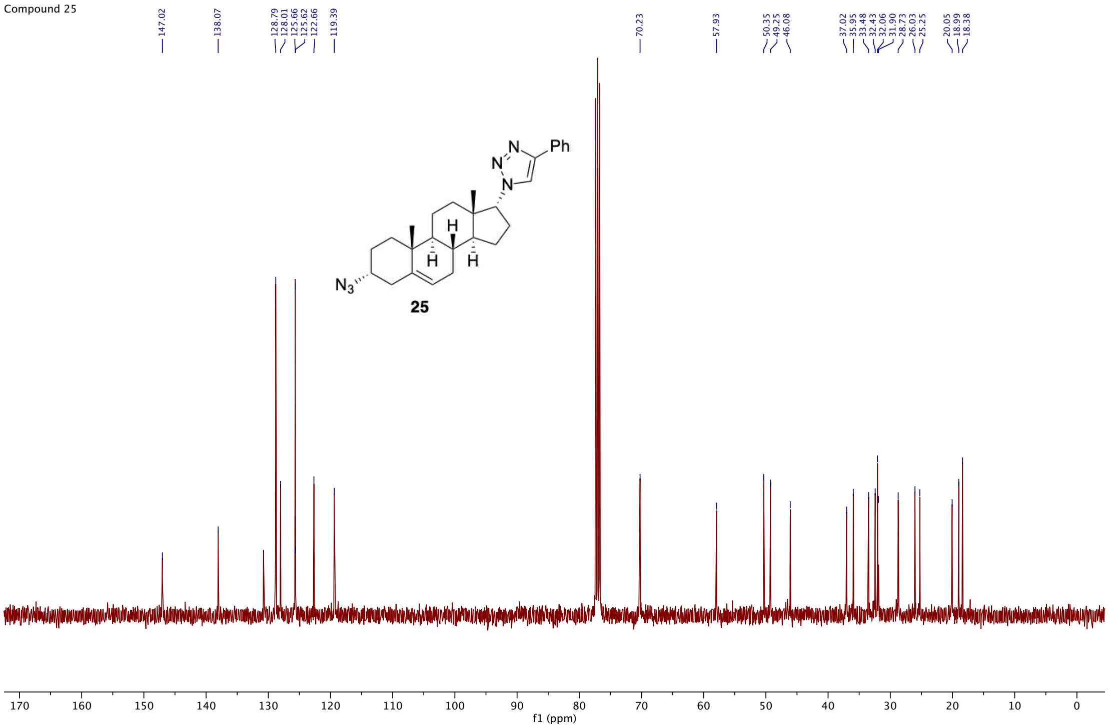

Single Mass Analysis

Tolerance = 1000.0 PPM / DBE: min = 0.0, max = 50.0
Element prediction: Off
Number of isotope peaks used for i-FIT = 3

Monoisotopic Mass, Even Electron Ions
34 formula(e) evaluated with 14 results within limits (up to 5 closest results for each mass)
Elements Used:
C: 5-50 H: 3-72 N: 1-6
Compound 25 19 (0.352) AM (Cen,4, 50.00, Ar,6000.0,490.89,0.80,LS 10); Cm (18:28)
1: TOF MS ES+

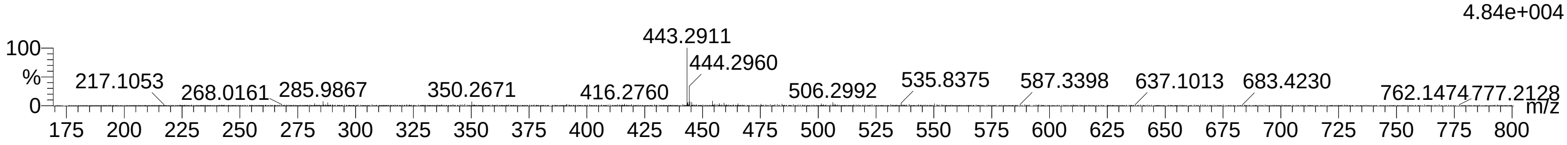

Minimum:				0.0		
Maximum:		5.0	1000.0	50.0		

Mass	Calc. Mass	mDa	PPM	DBE	i-FIT	Formula
443.2911	443.2923	-1.2	-2.7	13.5	22.6	C27 H35 N6
	443.3175	-26.4	-59.6	12.5	646.3	C29 H39 N4
	443.2487	42.4	95.6	18.5	1803.4	C32 H31 N2
	443.3426	-51.5	-116.2	11.5	2513.4	C31 H43 N2
	443.2236	67.5	152.3	19.5	4400.6	C30 H27 N4

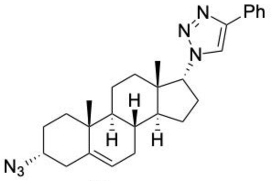

25

Compound 26

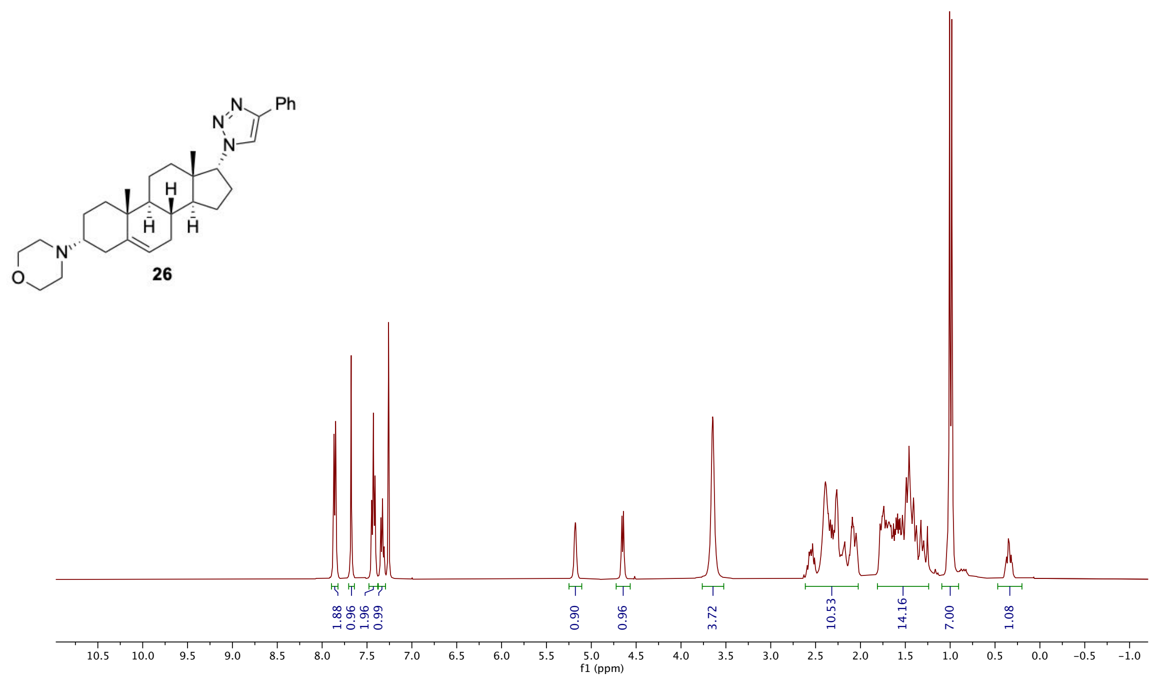

Compound 26

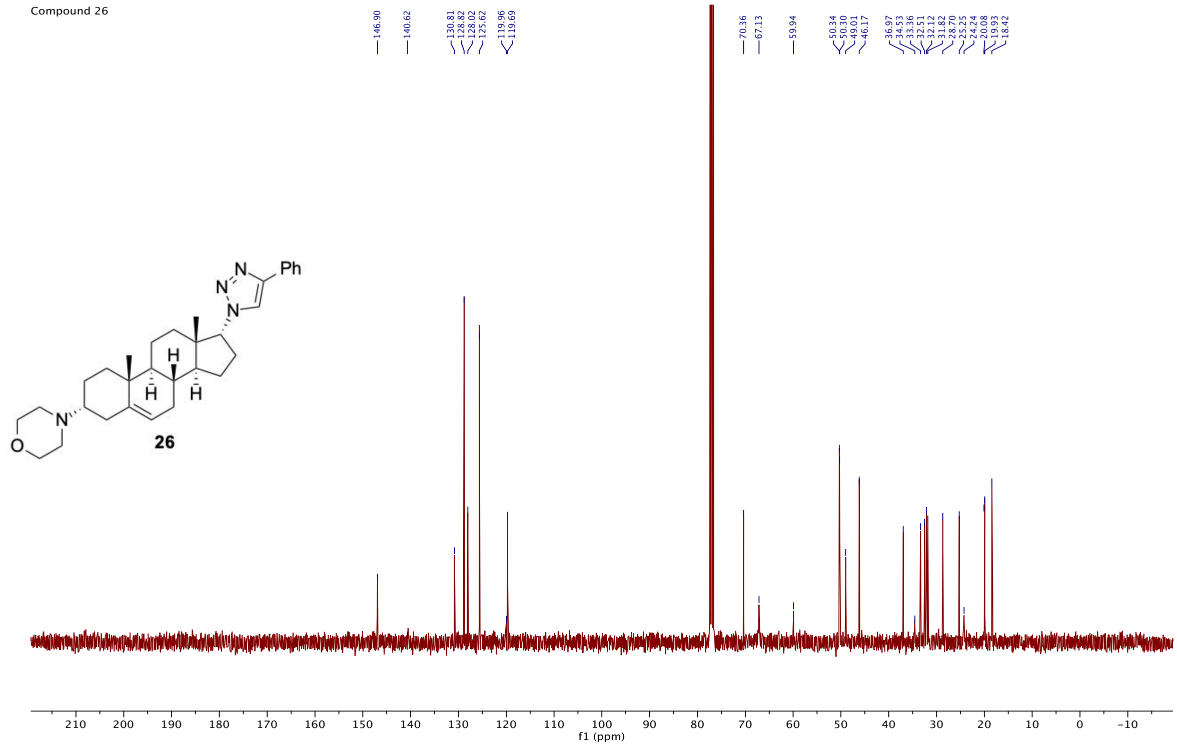

Single Mass Analysis

Tolerance = 1000.0 PPM / DBE: min = 0.0, max = 50.0
Element prediction: Off
Number of isotope peaks used for i-FIT = 3

Monoisotopic Mass, Even Electron Ions
272 formula(e) evaluated with 121 results within limits (up to 5 closest results for each mass)
Elements Used:
C: 5-50 H: 3-72 N: 1-6 O: 1-9

Compound 26 24 (0.452) AM (Cen,4, 50.00, Ar,6000.0,490.89,0.80,LS 10); Cm (16:29)
1: TOF MS ES+

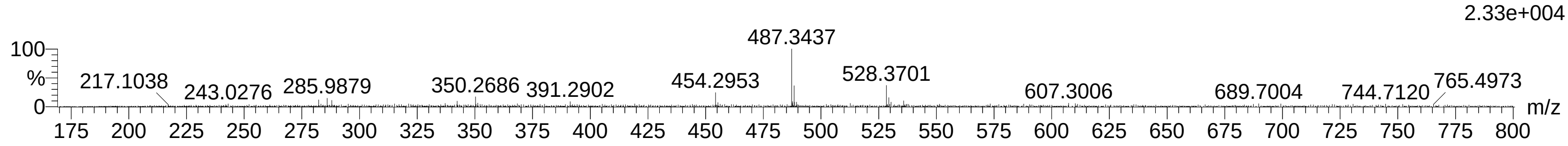

Minimum:				0.0		
Maximum:		5.0	1000.0	50.0		
Mass	Calc. Mass	mDa	PPM	DBE	i-FIT	Formula
487.3437	487.3437	0.0	0.0	12.5	16.1	C31 H43 N4 O
	487.3397	4.0	8.2	8.5	112.2	C26 H43 N6 O3
	487.3383	5.4	11.1	3.5	175.4	C25 H47 N2 O7
	487.3496	-5.9	-12.1	3.5	209.9	C24 H47 N4 O6
	487.3536	-9.9	-20.3	7.5	72.3	C29 H47 N2 O4

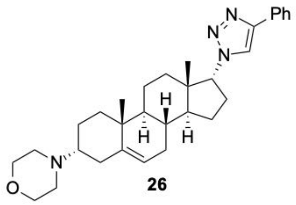

26

Compound 28

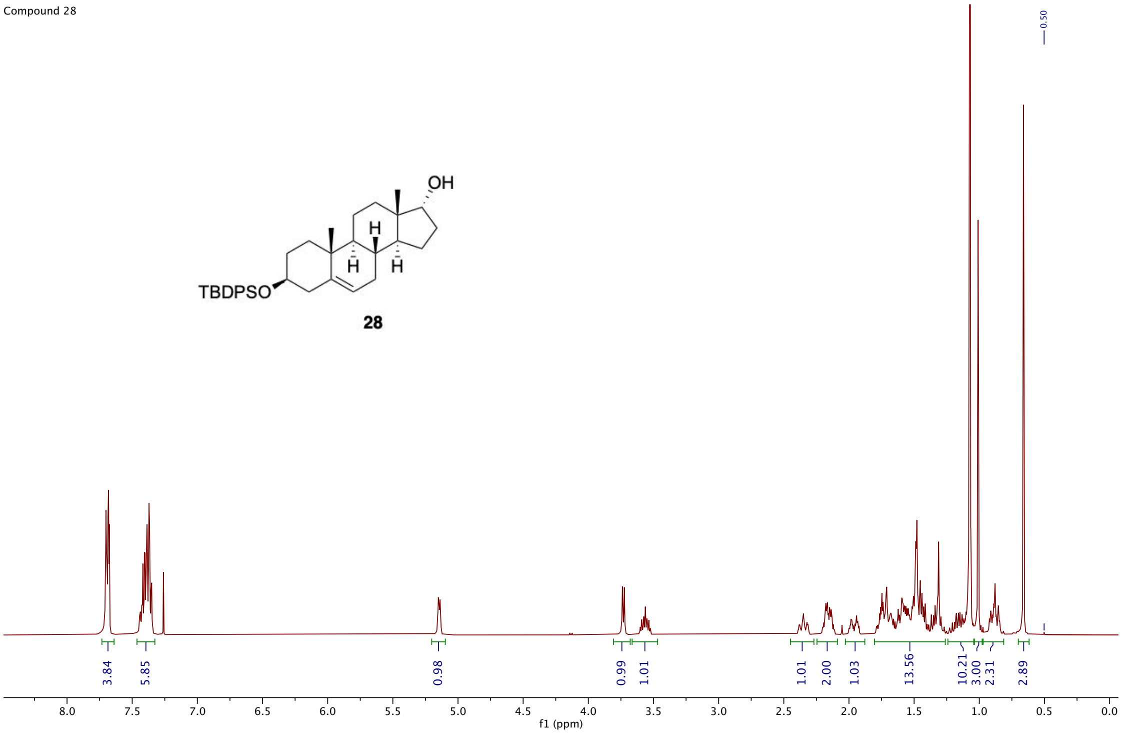

Compound 29

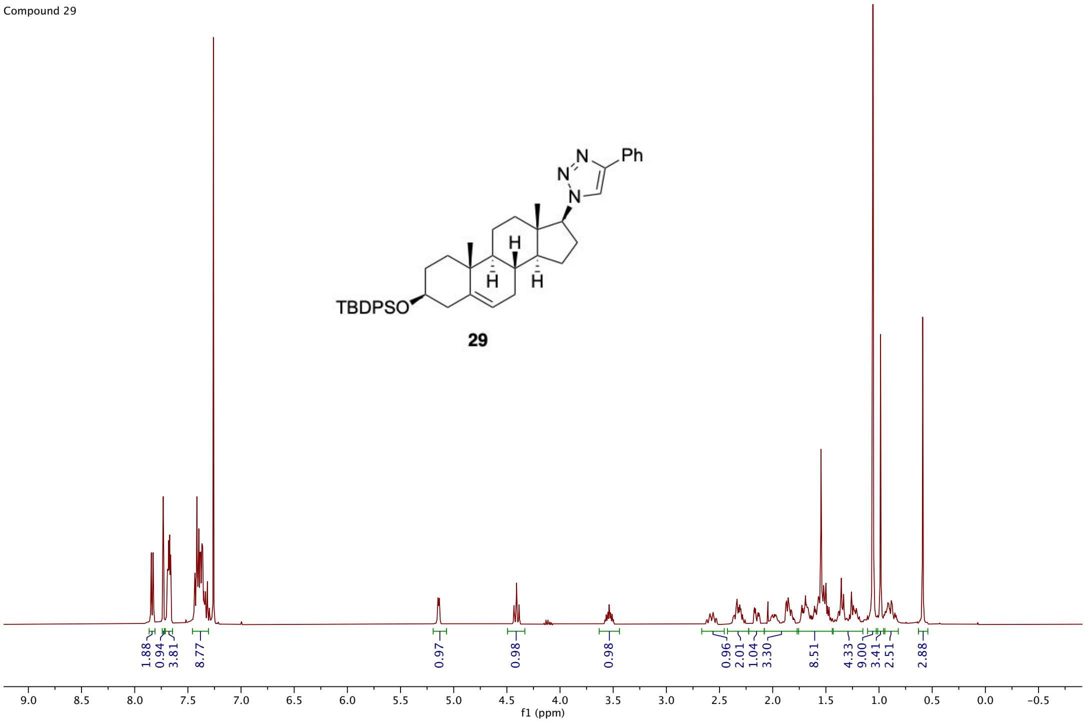

Compound 30

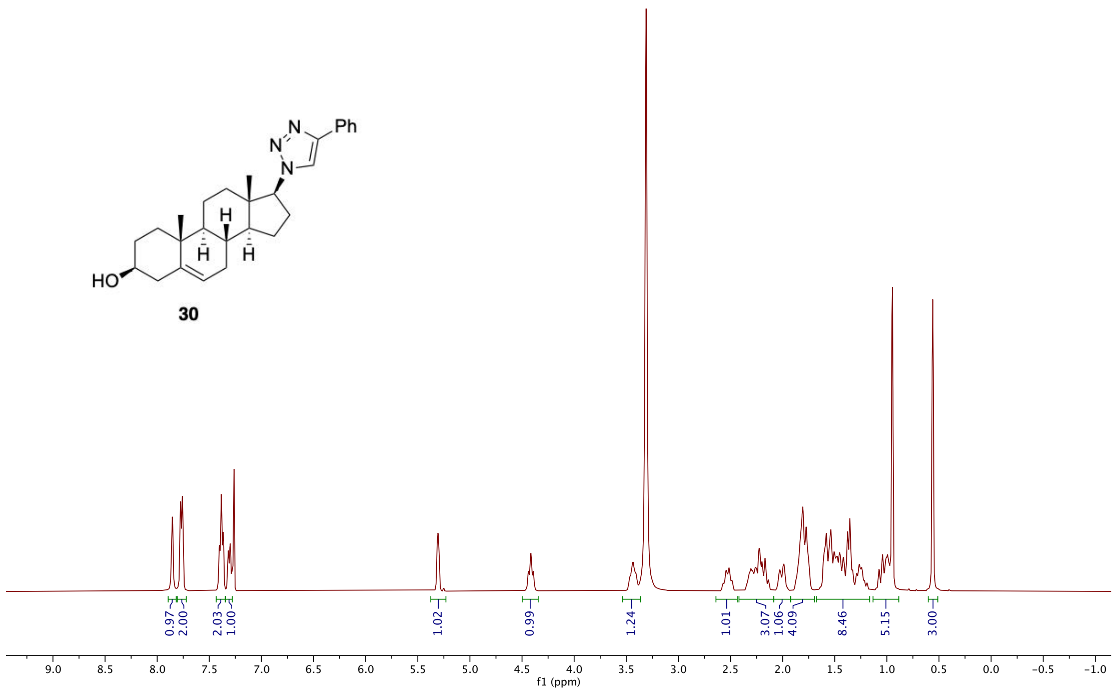

Compound 30

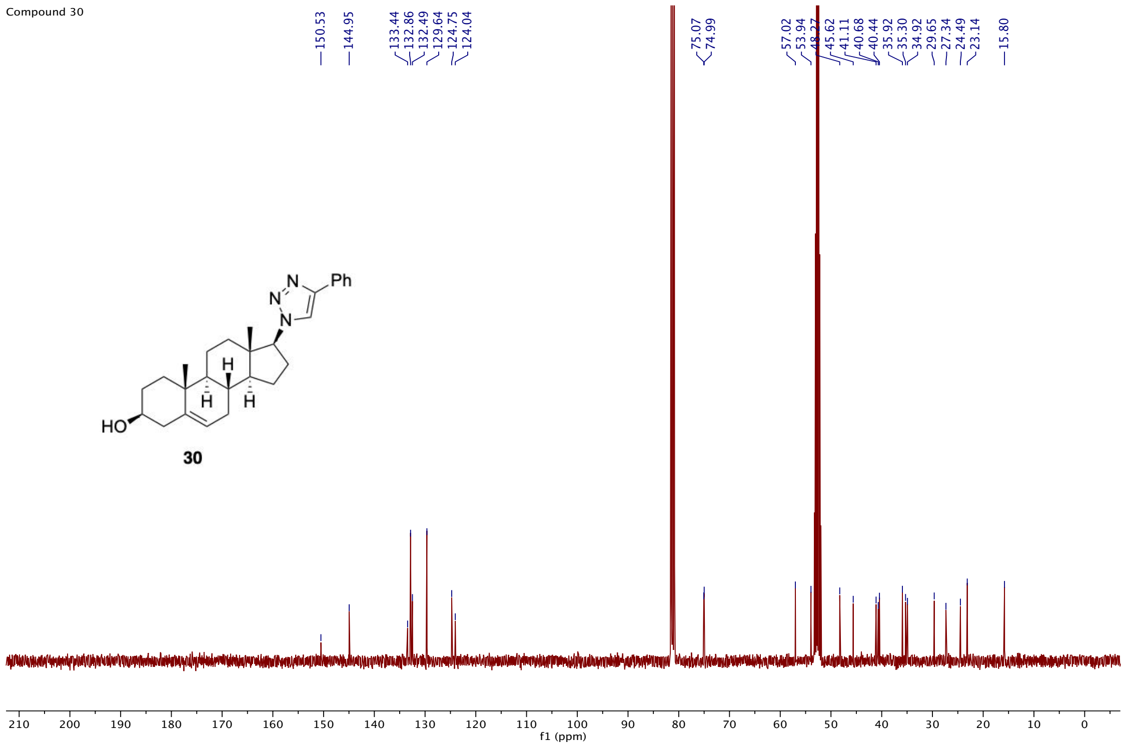

Compound 32

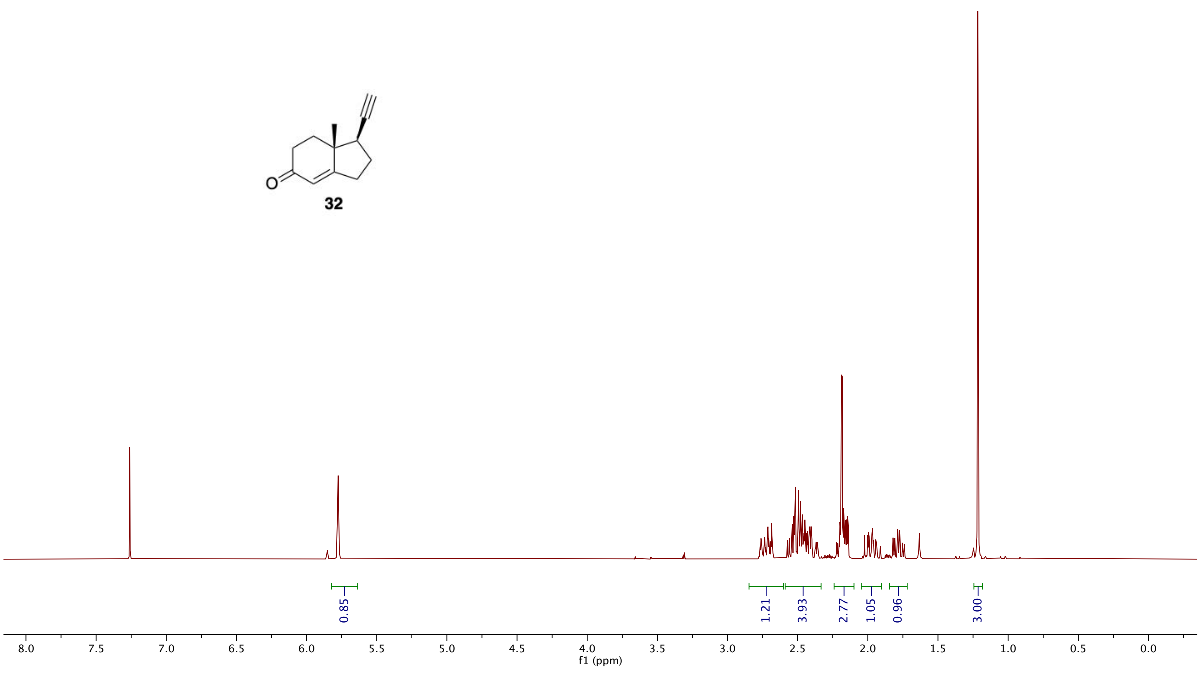

Compound 32

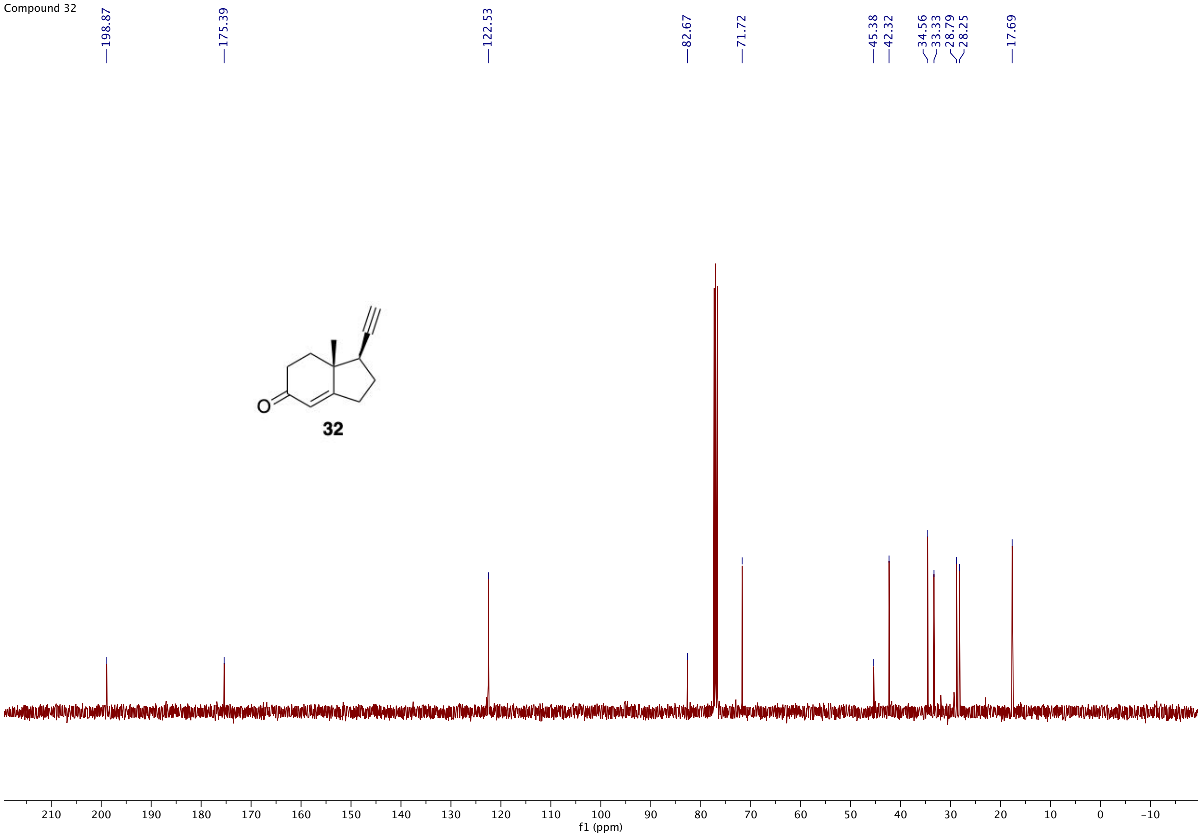

Elemental Composition Report

Single Mass Analysis

Tolerance = 1000.0 PPM / DBE: min = 0.0, max = 50.0
Element prediction: Off
Number of isotope peaks used for i-FIT = 3

Monoisotopic Mass, Even Electron Ions
9 formula(e) evaluated with 6 results within limits (up to 5 closest results for each mass)
Elements Used:
C: 5-50 H: 5-72 O: 1-5

Compound 32 18 (0.335) AM (Cen,4, 9.00, Ar,6000.0,294.94,0.80,LS 10); Cm (16:22)
1: TOF MS ES+

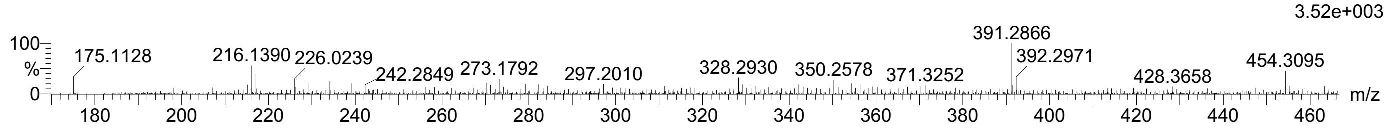

Minimum:				0.0		
Maximum:		5.0	1000.0	50.0		
Mass	Calc. Mass	mDa	PPM	DBE	i-FIT	Formula
175.1128	175.1123	0.5	2.9	5.5	13419.3	C12 H15 O
	175.0970	15.8	90.2	1.5	13015.6	C8 H15 O4
	175.1334	-20.6	-117.6	0.5	14017.9	C9 H19 O3
	175.0759	36.9	210.7	6.5	12620.1	C11 H11 O2
	175.0606	52.2	298.1	2.5	12375.6	C7 H11 O5

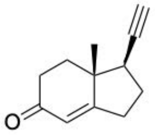

32

Compound 33

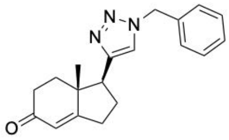

33

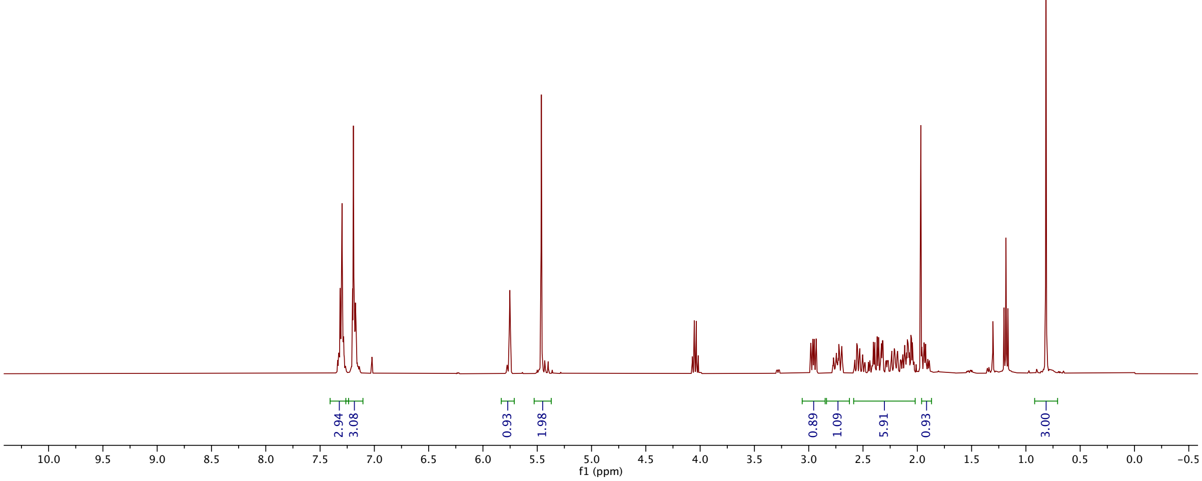

Single Mass Analysis
Tolerance = 1000.0 PPM / DBE: min = 0.0, max = 50.0
Element prediction: Off
Number of isotope peaks used for i-FIT = 3

Monoisotopic Mass, Even Electron Ions
120 formula(e) evaluated with 49 results within limits (up to 5 closest results for each mass)
Elements Used:
C: 5-50 H: 5-72 N: 1-8 O: 1-5
Compound 33 (0.335) AM (Cen,4, 50.00, Ar,6000.0,392.92,0.80,LS 10); Cm (16:21)
1: TOF MS ES+

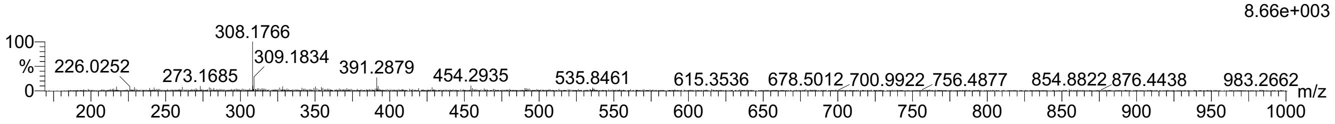

Minimum:				0.0		
Maximum:		5.0	1000.0	50.0		
Mass	Calc. Mass	mDa	PPM	DBE	i-FIT	Formula
308.1766	308.1763	0.3	1.0	10.5	81.4	C19 H22 N3 O
	308.1723	4.3	14.0	6.5	213.2	C14 H22 N5 O3
	308.1835	-6.9	-22.4	6.5	226.1	C13 H22 N7 O2
	308.1682	8.4	27.3	2.5	431.4	C9 H22 N7 O5
	308.1862	-9.6	-31.2	5.5	150.0	C17 H26 N O4

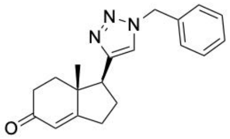

33

UPLC trace of compound 33

Vial:1:D,4
Date:29-Jun-2011

ID:
Time:14:52:01

Mobile Phase A (10 mM ammonium bicarbonate in water), mobile Phase B (ACN), a flow rate of 0.6 mL/min, injection volume 7.5 µL, run time 6.0 min

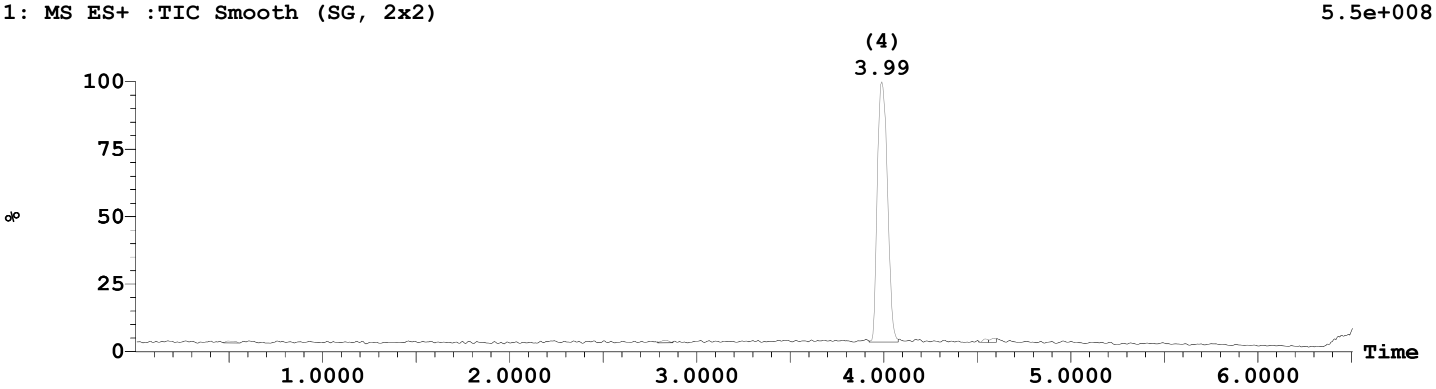

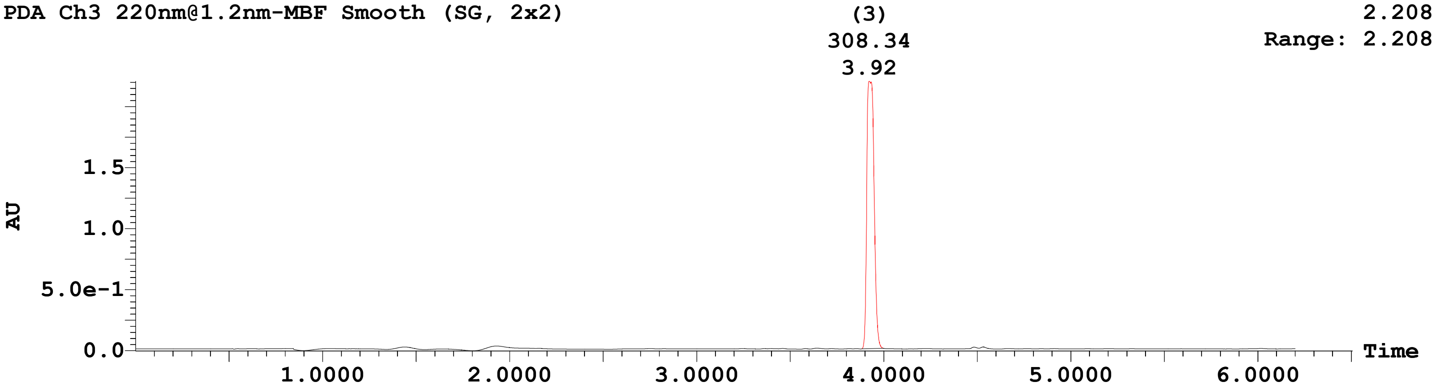

Peak Number	Compound	Time	AreaAbs	Area %Total	Width	Height	Mass Found
3		3.92	9.91e+004	100.00	0	2e+006	

Peak ID	Time	Mass Found	TIC
3	3.92		[illegible]9088

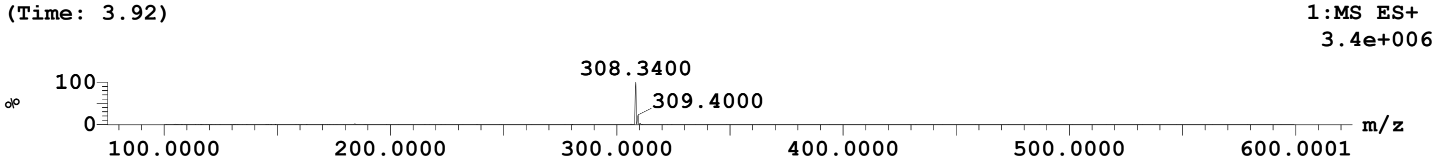

Compound 34a+34b

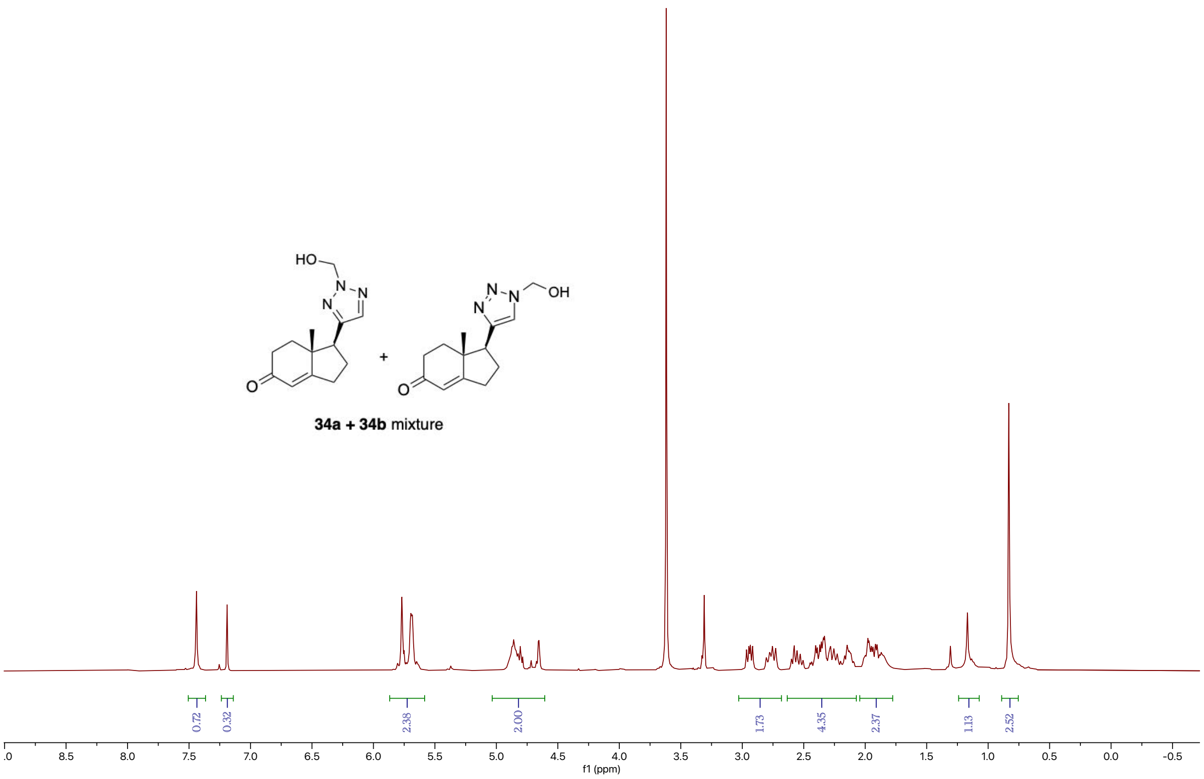

Compound 35

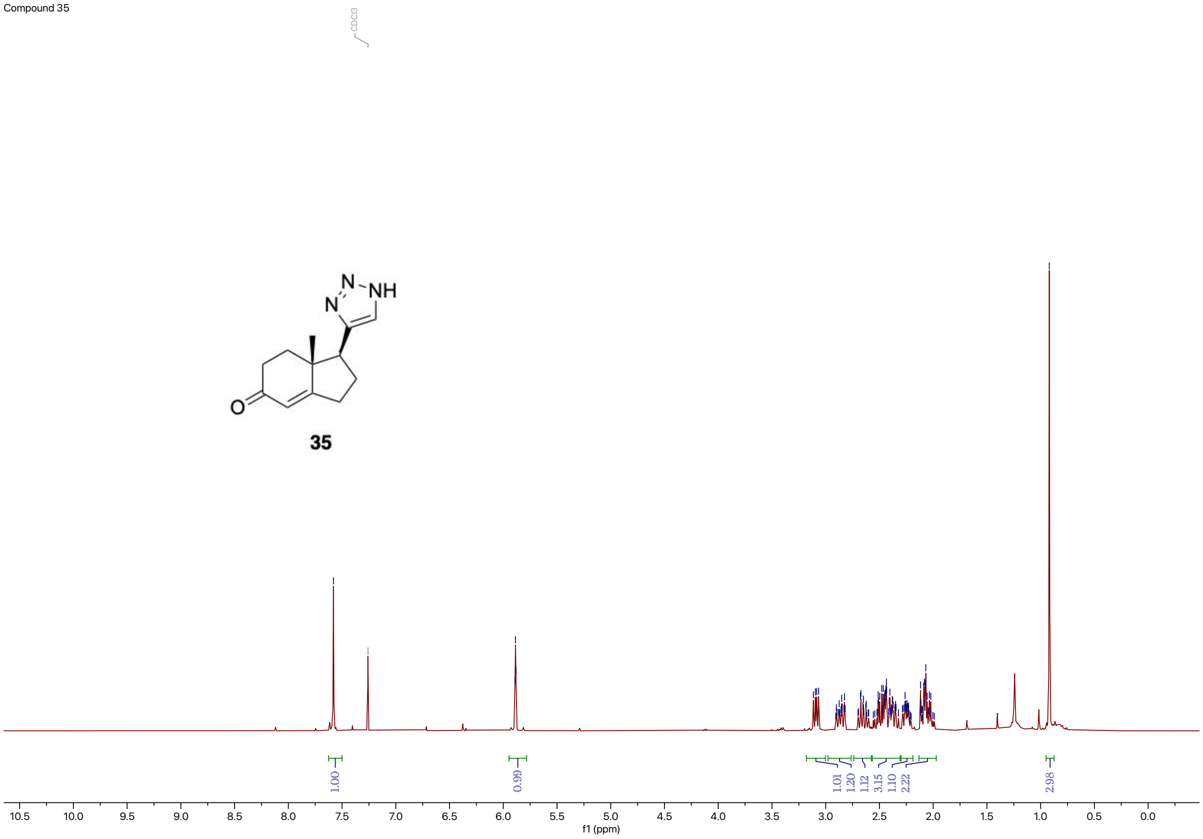

Compound 35

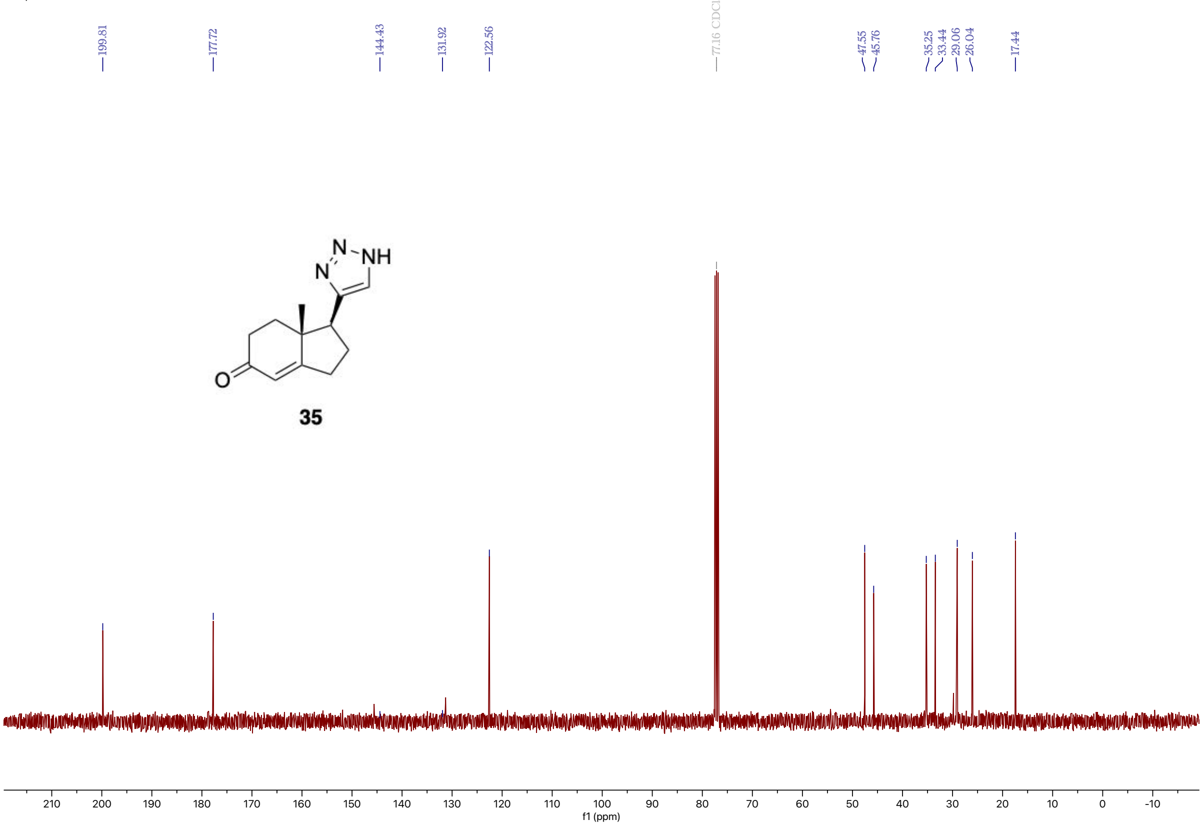

Single Mass Analysis

Tolerance = 1000.0 PPM / DBE: min = 0.0, max = 50.0
Element prediction: Off
Number of isotope peaks used for i-FIT = 3

Monoisotopic Mass, Even Electron Ions
63 formula(e) evaluated with 26 results within limits (up to 5 closest results for each mass)
Elements Used:
C: 5-50 H: 5-72 N: 1-8 O: 1-5

Compound 35 18 (0.335) AM (Cen,4, 90.00, Ar,6000.0,196.96,0.80,LS 10); Cm (16:22)
1: TOF MS ES+

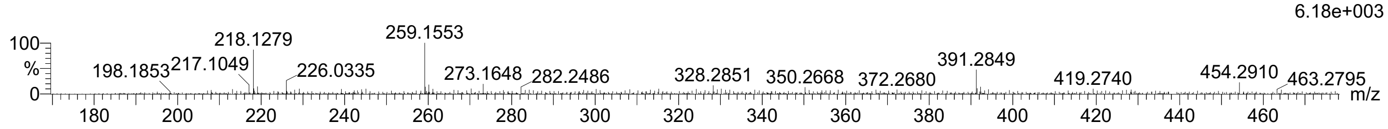

Minimum:				0.0		
Maximum:		5.0	1000.0	50.0		
Mass	Calc. Mass	mDa	PPM	DBE	i-FIT	Formula
218.1279	218.1293	-1.4	-6.4	6.5	37.1	C12 H16 N3 O
	218.1253	2.6	11.9	2.5	106.9	C7 H16 N5 O3
	218.1365	-8.6	-39.4	2.5	143.6	C6 H16 N7 O2
	218.1181	9.8	44.9	6.5	67.7	C13 H16 N O2
	218.1392	-11.3	-51.8	1.5	98.5	C10 H20 N O4

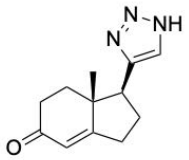

35

UPLC trace of compound 35

Vial:1:D,5 ID:
Date:29-Jun-2011 Time:14:59:37

Mobile Phase A (10 mM ammonium bicarbonate in water), mobile Phase B (ACN), a flow rate of 0.6 mL/min, injection volume 7.5 μL, run time 6.0 min

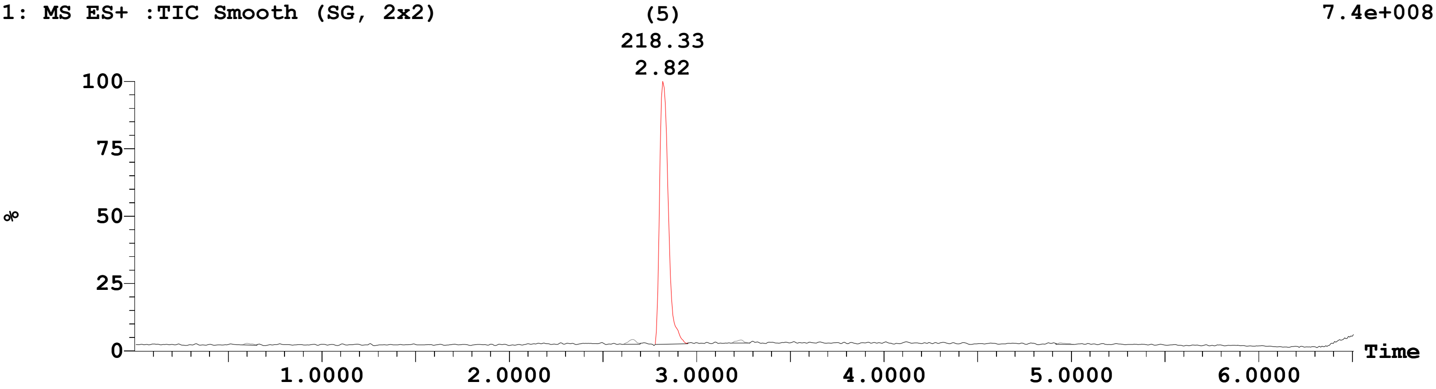

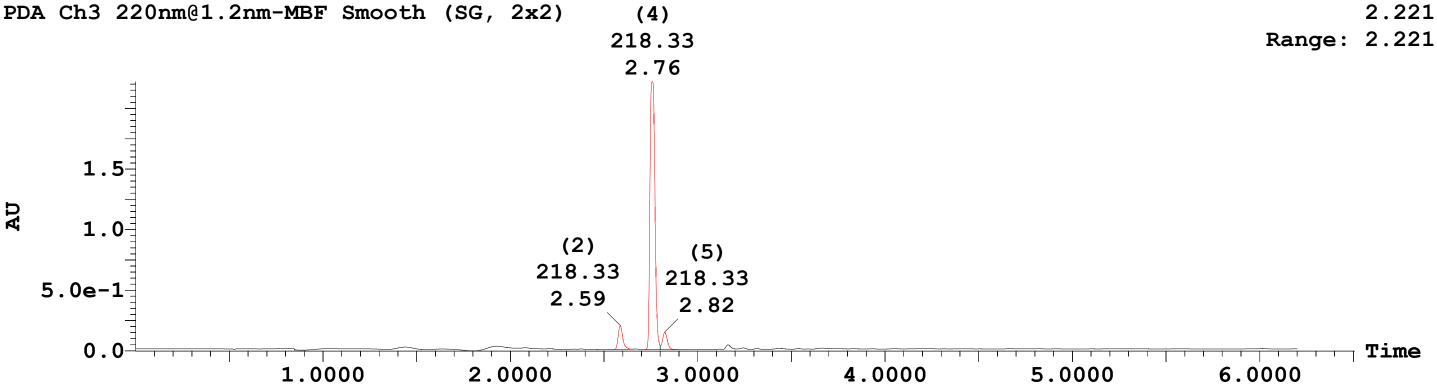

Peak Number	Compound	Time	AreaAbs	Area %Total	Width	Height	Mass Found
2		2.59	5.53e+003	7.07	0	2e+005	
4		2.76	6.84e+004	87.52	0	2e+006	
5		2.82	4.24e+003	5.42	0	1e+005	

Peak ID	Time	Mass Found	TIC
2	2.59		[illegible]8080

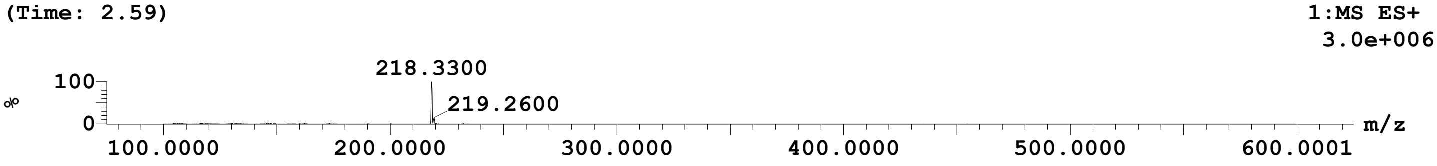

Peak ID	Time	Mass Found	TIC
4	2.76		[illegible]0932

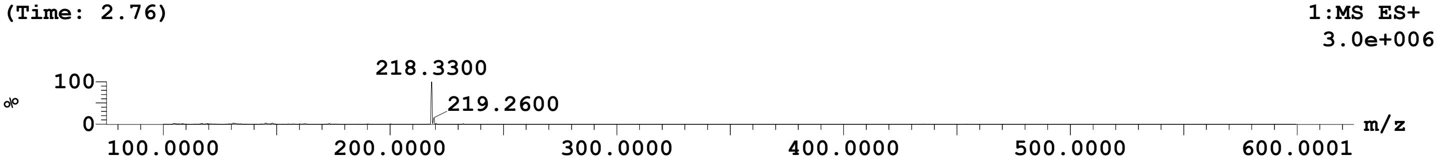

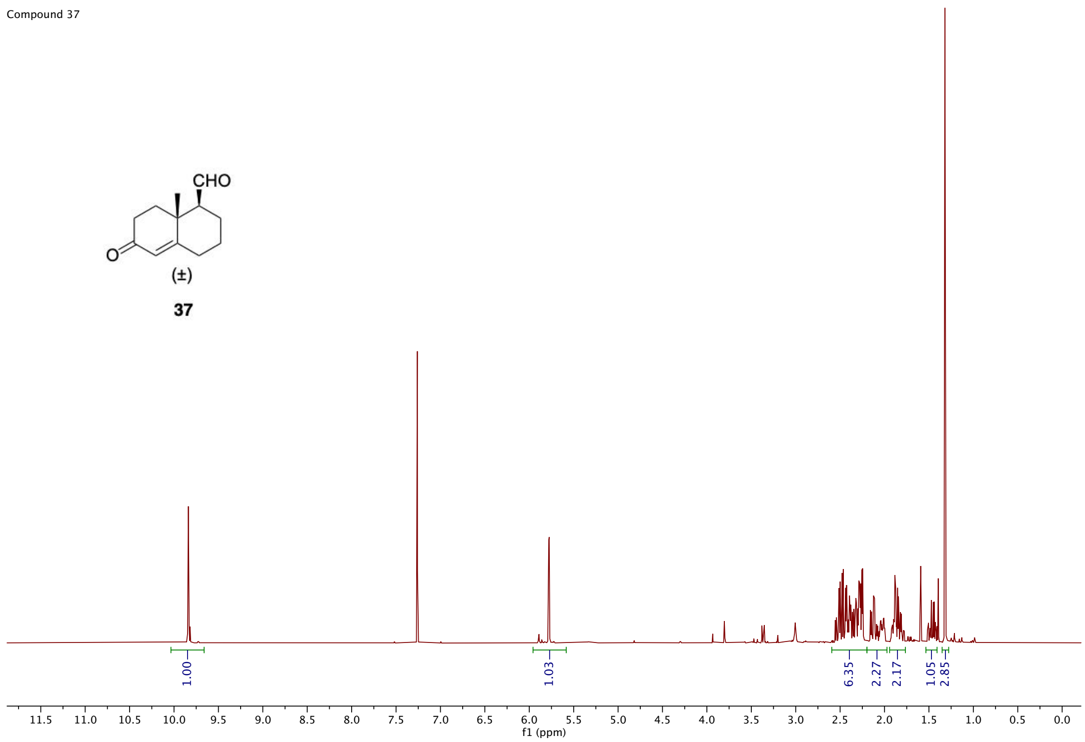
Compound 37
CHO
O
(±)
37
1.00
1.03
6.35
2.27
2.17
1.05
2.85
11.5
11.0
10.5
10.0
9.5
9.0
8.5
8.0
7.5
7.0
6.5
6.0
5.5
5.0
4.5
4.0
3.5
3.0
2.5
2.0
1.5
1.0
0.5
0.0
f1 (ppm)

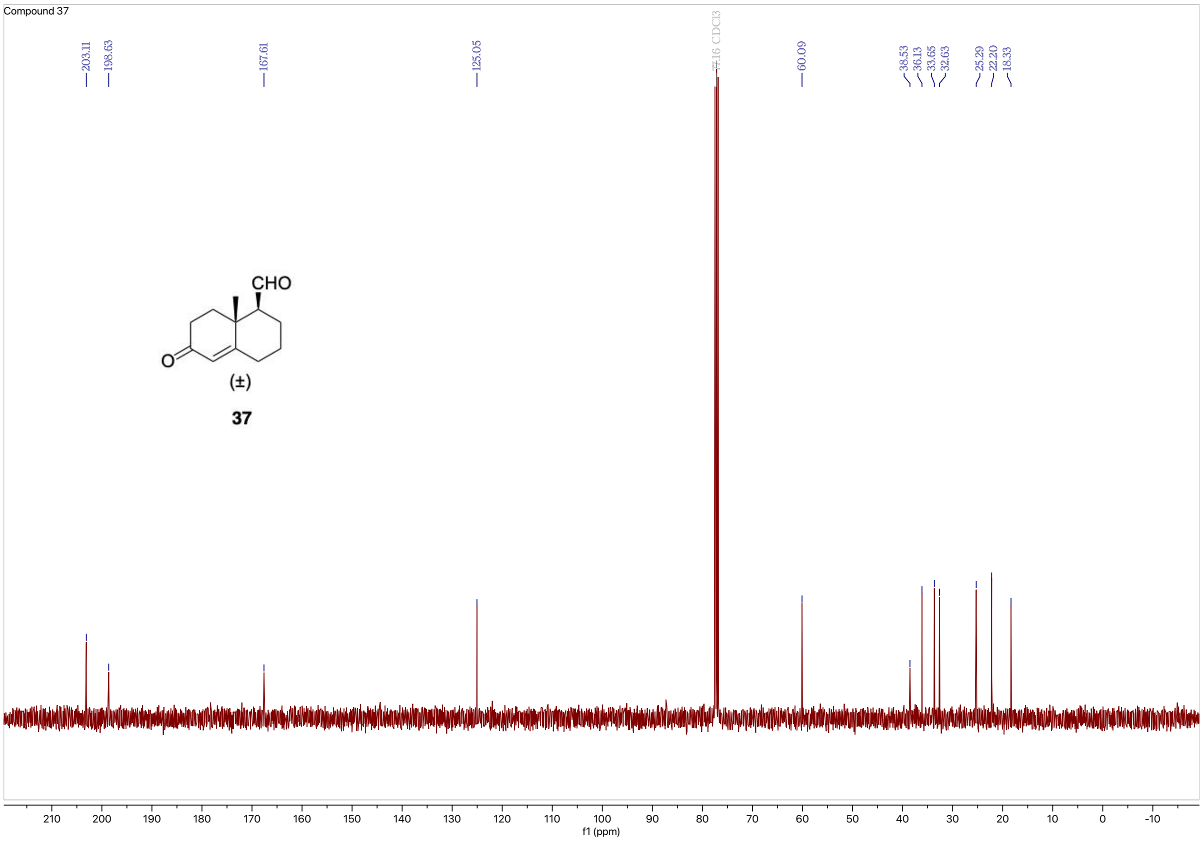
Compound 37
203.11
198.63
167.61
125.05
77.16 CDCl3
60.09
38.53
36.13
33.65
32.63
25.29
22.20
18.33
CHO
O
(±)
37
210
200
190
180
170
160
150
140
130
120
110
100
90
80
70
60
50
40
30
20
10
0
-10
f1 (ppm)

Compound 38

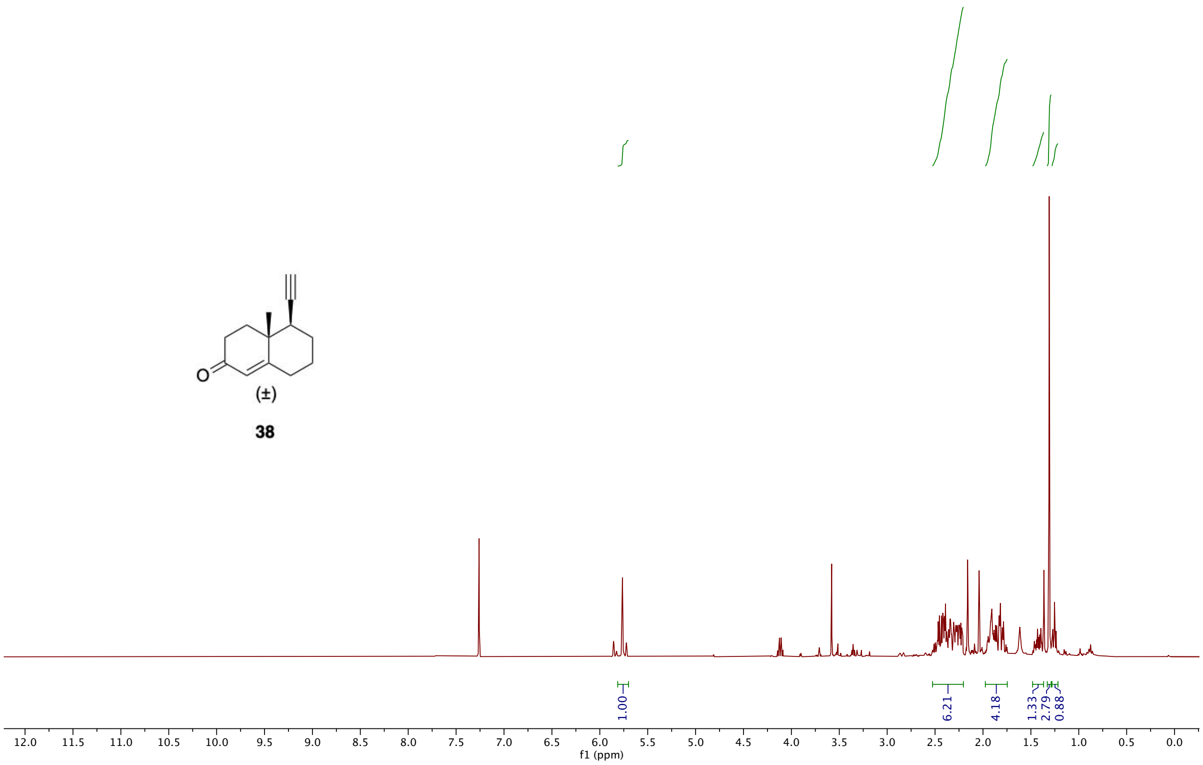

Compound 39

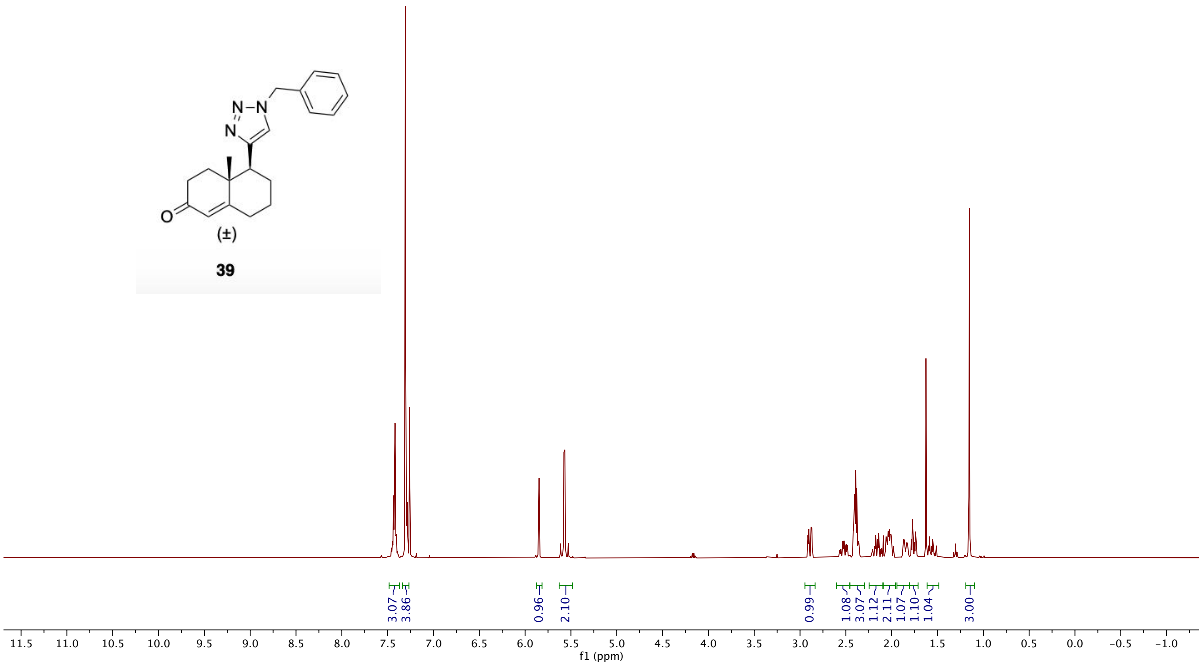

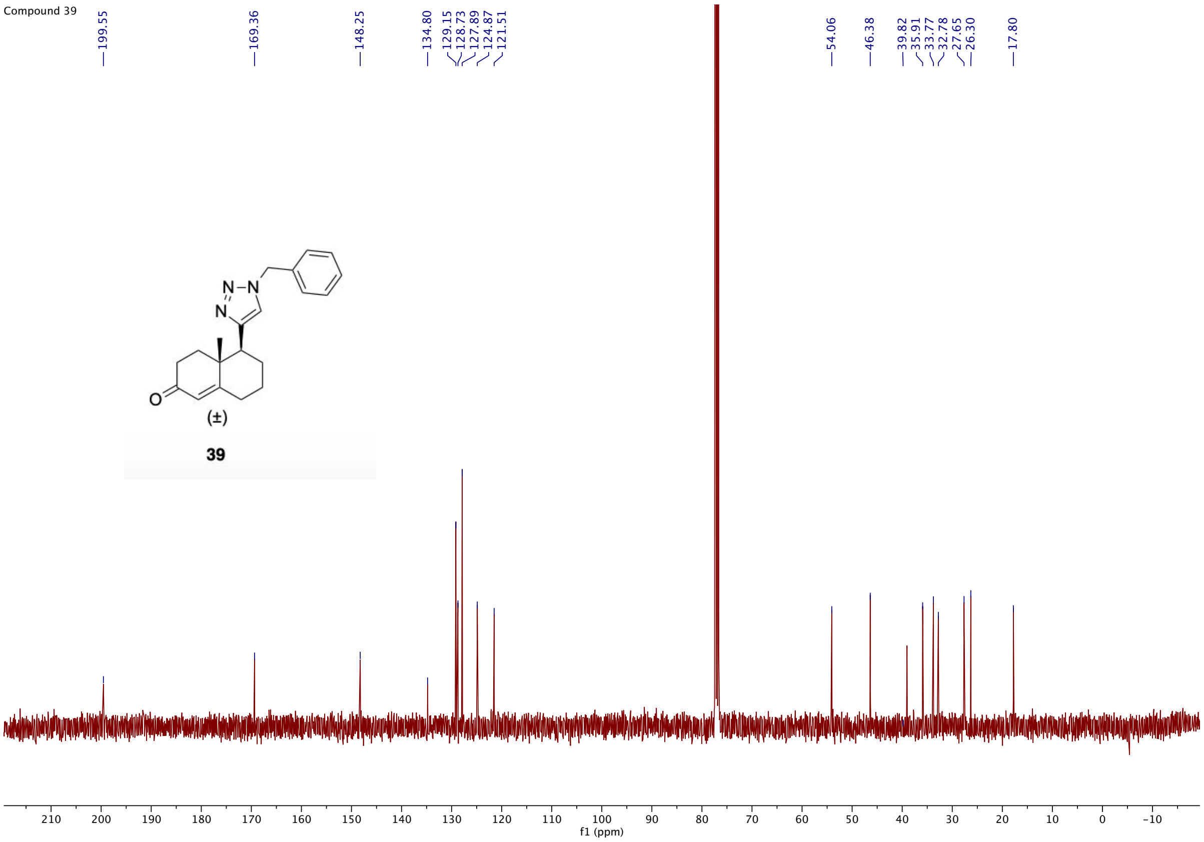
Compound 39
199.55
169.36
148.25
134.80
129.15
128.73
127.89
124.87
121.51
54.06
46.38
39.82
35.91
33.77
32.78
27.65
26.30
17.80
(±)
39
210 200 190 180 170 160 150 140 130 120 110 100 90 80 70 60 50 40 30 20 10 0 -10
f1 (ppm)

Single Mass Analysis

Tolerance = 1000.0 PPM / DBE: min = 0.0, max = 50.0
Element prediction: Off
Number of isotope peaks used for i-FIT = 3

Monoisotopic Mass, Even Electron Ions
126 formula(e) evaluated with 49 results within limits (up to 5 closest results for each mass)
Elements Used:
C: 5-50 H: 5-72 N: 1-8 O: 1-5
Compound 39 19 (0.352) AM (Cen,4, 99.00, Ar,6000.0,392.92,0.80,LS 10); Cm (15:24)
1: TOF MS ES+

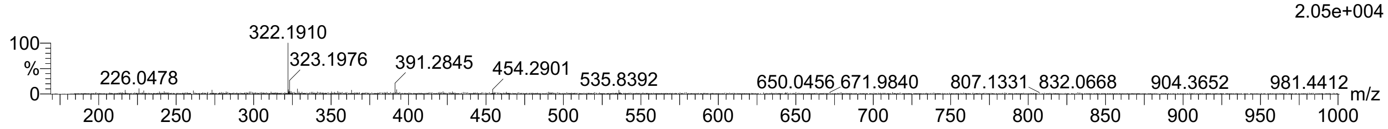

Minimum:				0.0		
Maximum:		5.0	1000.0	50.0		
Mass	Calc. Mass	mDa	PPM	DBE	i-FIT	Formula
322.1910	322.1919	-0.9	-2.8	10.5	100.5	C20 H24 N3 O
	322.1879	3.1	9.6	6.5	320.3	C15 H24 N5 O3
	322.1839	7.1	22.0	2.5	735.7	C10 H24 N7 O5
	322.1991	-8.1	-25.1	6.5	396.9	C14 H24 N7 O2
	322.1807	10.3	32.0	10.5	163.1	C21 H24 N O2

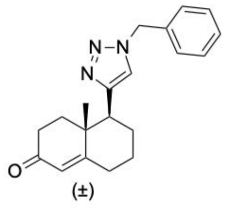

39

Compound 40

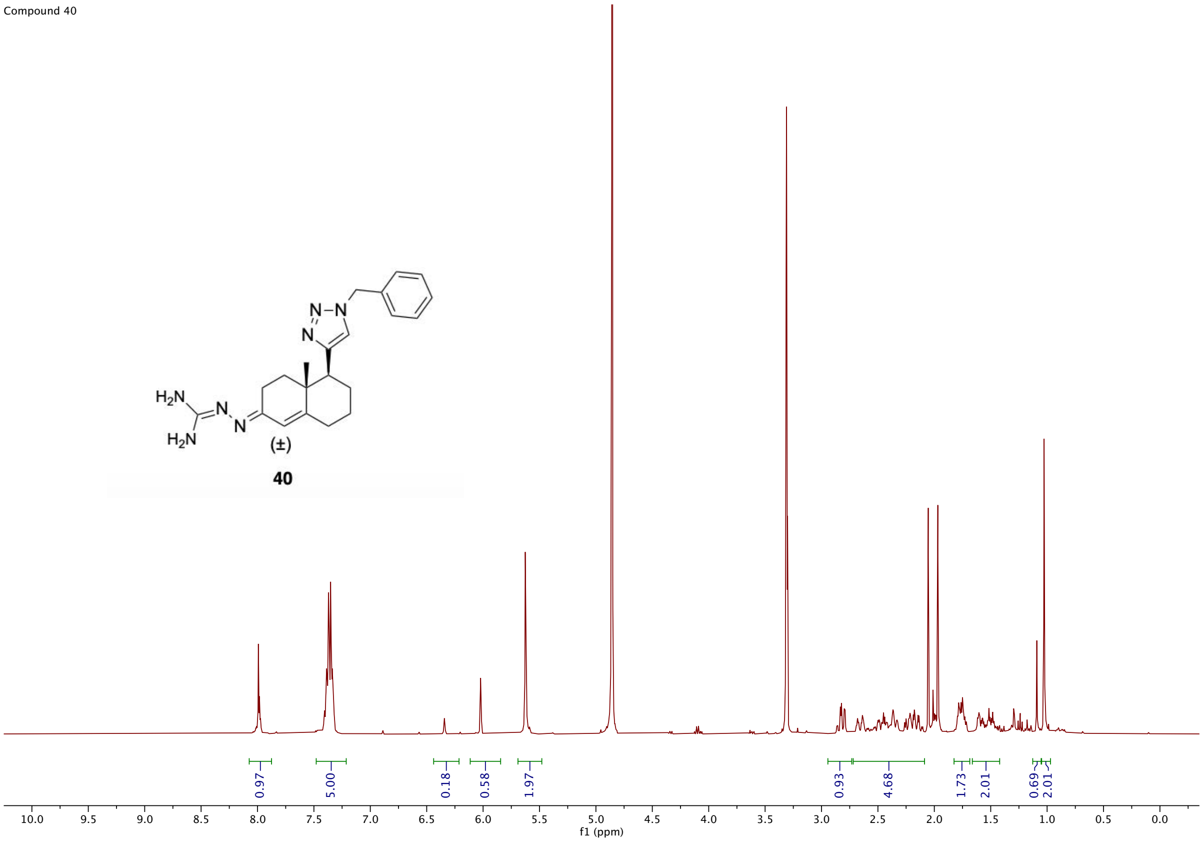
H2N
H2N
N
N
N
N
N
N
(±)
40
0.97
5.00
0.18
0.58
1.97
0.93
4.68
1.73
2.01
0.69
2.01
10.0 9.5 9.0 8.5 8.0 7.5 7.0 6.5 6.0 5.5 5.0 4.5 4.0 3.5 3.0 2.5 2.0 1.5 1.0 0.5 0.0
f1 (ppm)

Single Mass Analysis

Tolerance = 1000.0 PPM / DBE: min = 0.0, max = 50.0
Element prediction: Off
Number of isotope peaks used for i-FIT = 3

Monoisotopic Mass, Even Electron Ions
30 formula(e) evaluated with 15 results within limits (up to 5 closest results for each mass)
Elements Used:
C: 5-50 H: 5-72 N: 1-7
Compound 40 19 (0.352) AM (Cen,4, 99.00, Ar,6000.0,294.94,0.80,LS 10); Cm (15:21)
1: TOF MS ES+

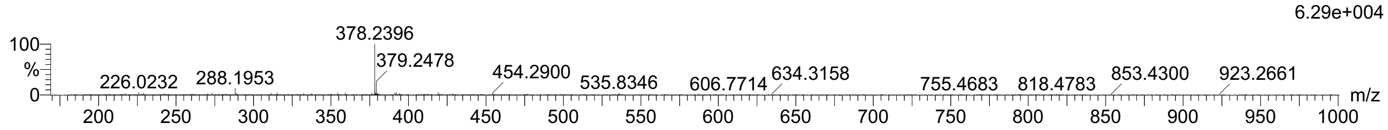

Mass	Calc. Mass	mDa	PPM	DBE	i-FIT	Formula
Minimum:				0.0		
Maximum:		5.0	1000.0	50.0		
378.2396	378.2406	-1.0	-2.6	11.5	22.0	C21 H28 N7
	378.2222	17.4	46.0	15.5	1128.6	C28 H28 N
	378.2658	-26.2	-69.3	10.5	1083.6	C23 H32 N5
	378.1970	42.6	112.6	16.5	3342.8	C26 H24 N3
	378.2909	-51.3	-135.6	9.5	4200.9	C25 H36 N3

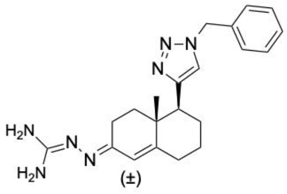

40

UPLC trace of compound 40

Vial:1:18
Date:30-Jul-2009

ID:
Time:12:39:23

Mobile Phase A (10 mM ammonium bicarbonate in water), mobile Phase B (MeOH), a flow rate of 0.6 mL/min, injection volume 7.5 µL, run time 6.0 min

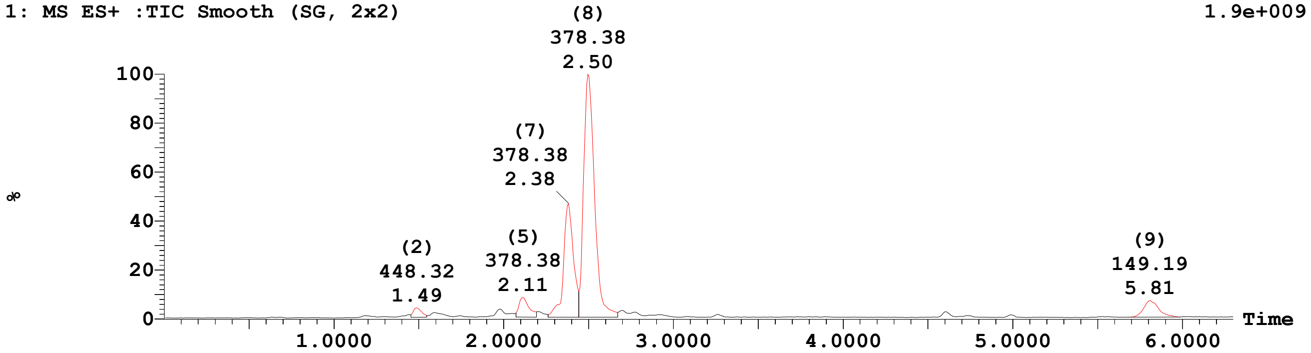

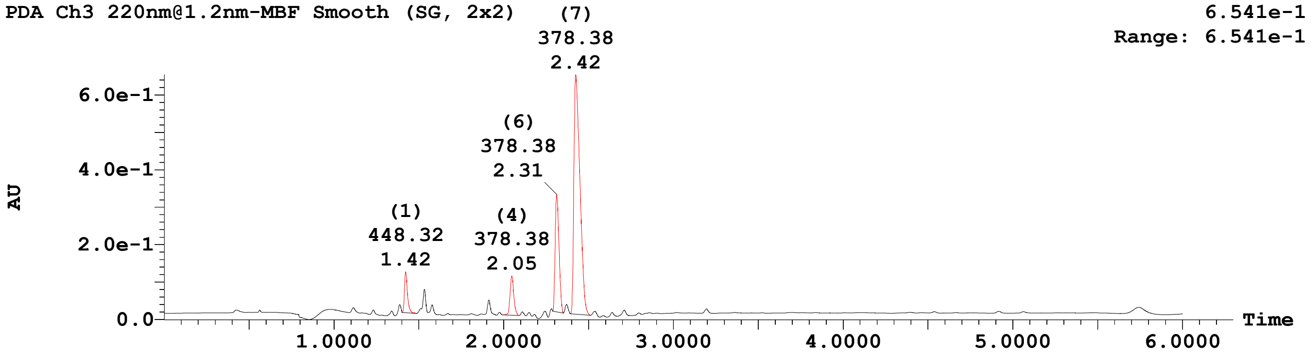

Peak Number	Compound	Time	AreaAbs	Area %Total	Width	Height	Mass Found
1		1.42	2.25e+003	5.69	0	1e+005	
4		2.05	2.68e+003	6.78	0	1e+005	
6		2.31	8.23e+003	20.81	0	3e+005	
7		2.42	2.64e+004	66.72	0	6e+005	

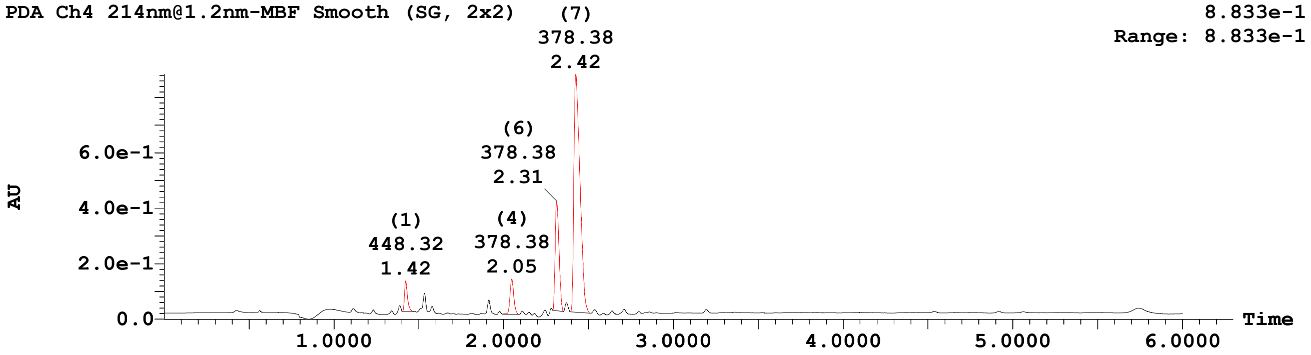

Peak Number	Compound	Time	AreaAbs	Area %Total	Width	Height	Mass Found
1		1.42	2.26e+003	4.37	0	1e+005	
4		2.05	3.29e+003	6.37	0	1e+005	
6		2.31	1.04e+004	20.20	0	4e+005	
7		2.42	3.57e+004	69.05	0	9e+005	

Compound 42

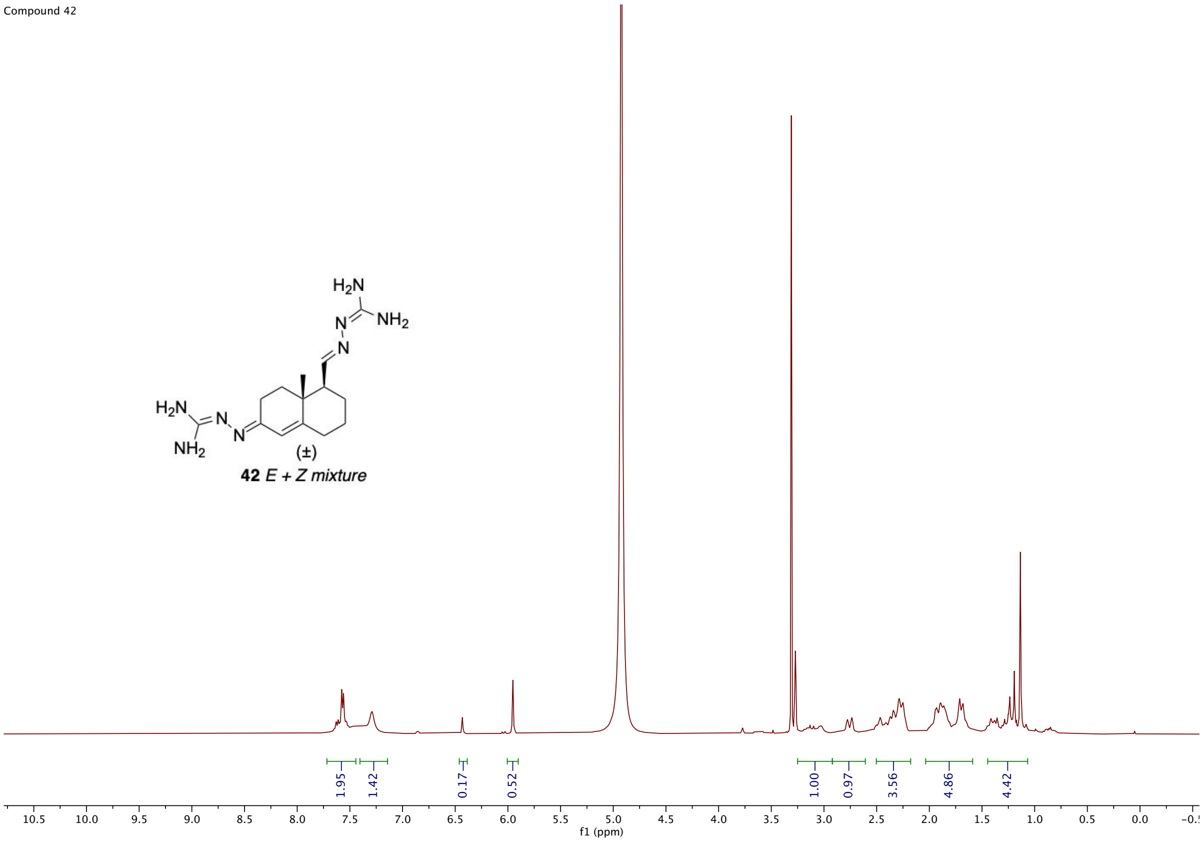
H2N
NH2
N
H2N
NH2
(±)
42 E + Z mixture
1.95
1.42
0.17
0.52
1.00
0.97
3.56
4.86
4.42
10.5 10.0 9.5 9.0 8.5 8.0 7.5 7.0 6.5 6.0 5.5 5.0 4.5 4.0 3.5 3.0 2.5 2.0 1.5 1.0 0.5 0.0
f1 (ppm)

Single Mass Analysis
Tolerance = 1000.0 PPM / DBE: min = 0.0, max = 50.0
Element prediction: Off
Number of isotope peaks used for i-FIT = 3

Monoisotopic Mass, Even Electron Ions
30 formula(e) evaluated with 11 results within limits (up to 5 closest results for each mass)
Elements Used:
C: 5-50 H: 5-72 N: 1-8
Compound 42 23 (0.435) AM (Cen,4, 9.00, Ar,6000.0,294.94,0.80,LS 10); Cm (18:29)
1: TOF MS ES+

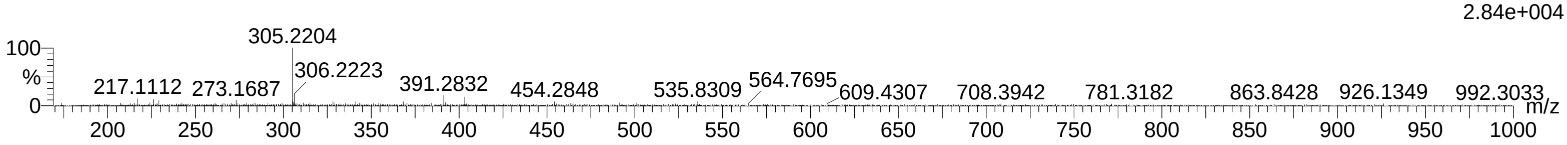

Minimum:				0.0		
Maximum:		5.0	1000.0	50.0		

Mass	Calc. Mass	mDa	PPM	DBE	i-FIT	Formula
305.2204	305.2202	0.2	0.7	6.5	146.6	C14 H25 N8
	305.2018	18.6	60.9	10.5	499.5	C21 H25 N2
	305.2454	-25.0	-81.9	5.5	753.2	C16 H29 N6
	305.1766	43.8	143.5	11.5	2103.0	C19 H21 N4
	305.2705	-50.1	-164.1	4.5	2715.7	C18 H33 N4

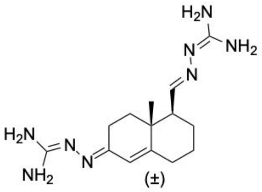

(±)
42 *E* + *Z* mixture

Vial:1:D,1 ID:
Date:29-Jun-2011 Time:15:37:41

Mobile Phase A (10 mM ammonium bicarbonate in water), mobile Phase B (MeOH), a flow rate of 0.6 mL/min, injection volume 7.5 µL, run time 6.0 min

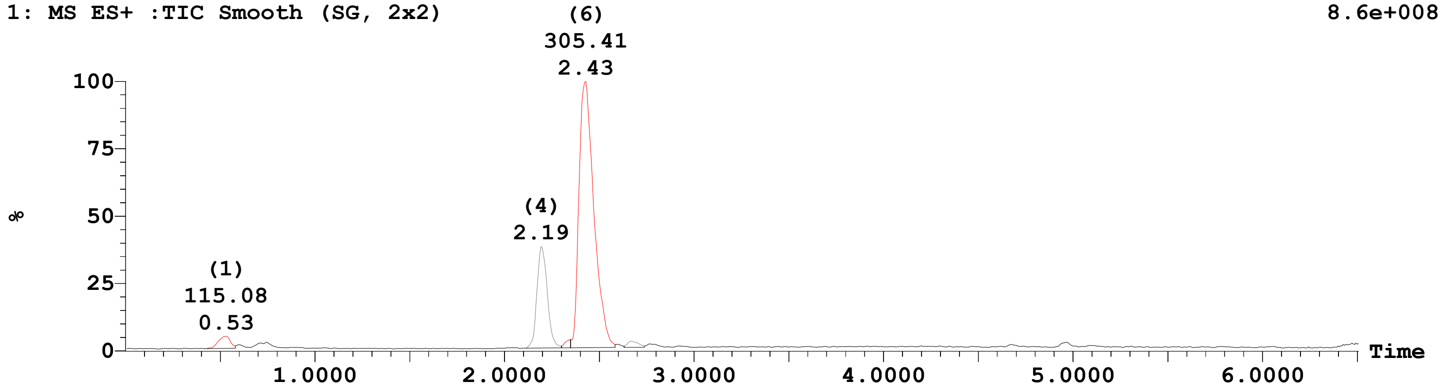

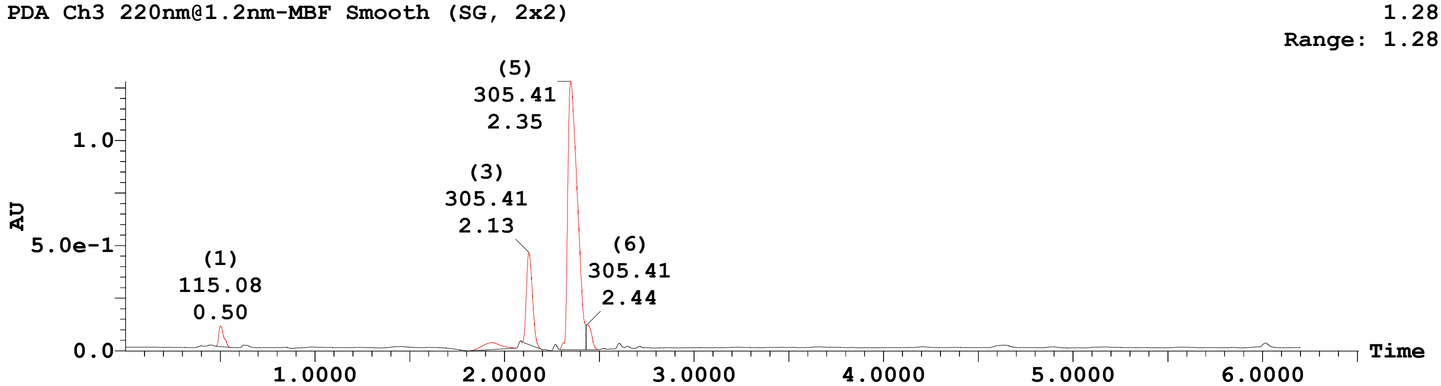

Peak Number	Compound	Time	AreaAbs	Area %Total	Width	Height	Mass Found
1		0.50	3.03e+003	2.98	0	1e+005	
2		1.93	3.64e+003	3.59	0	3e+004	
3		2.13	1.62e+004	15.92	0	4e+005	
5		2.35	7.49e+004	73.79	0	1e+006	
6		2.44	3.77e+003	3.72	0	1e+005	

Peak ID	Time	Mass Found	TIC
1	0.53		[illegible]8156

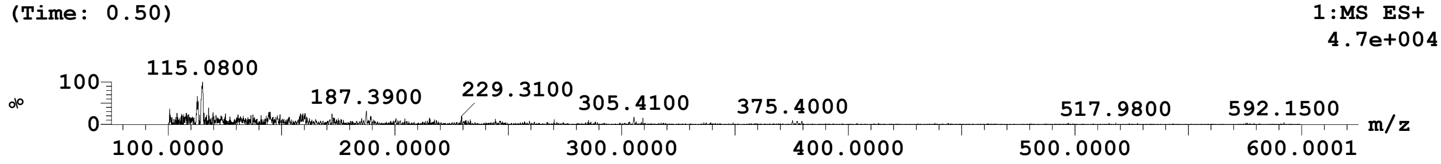

Compound 43

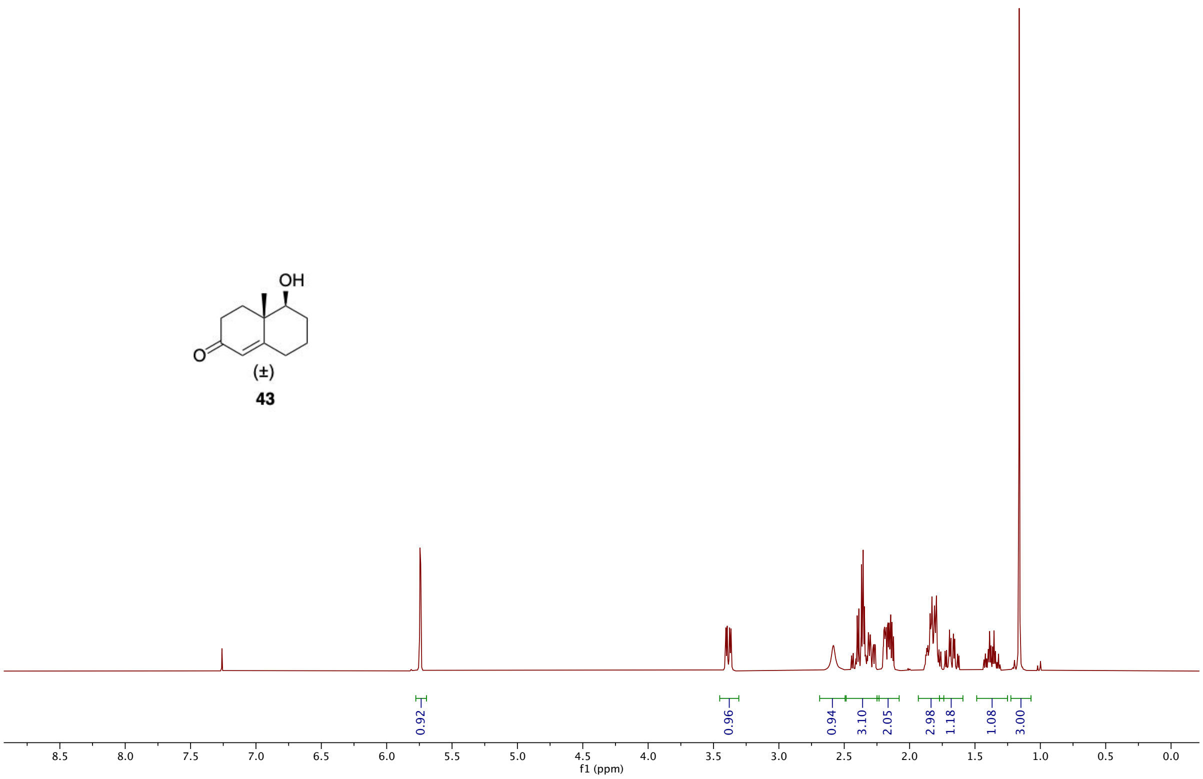

Compound 43

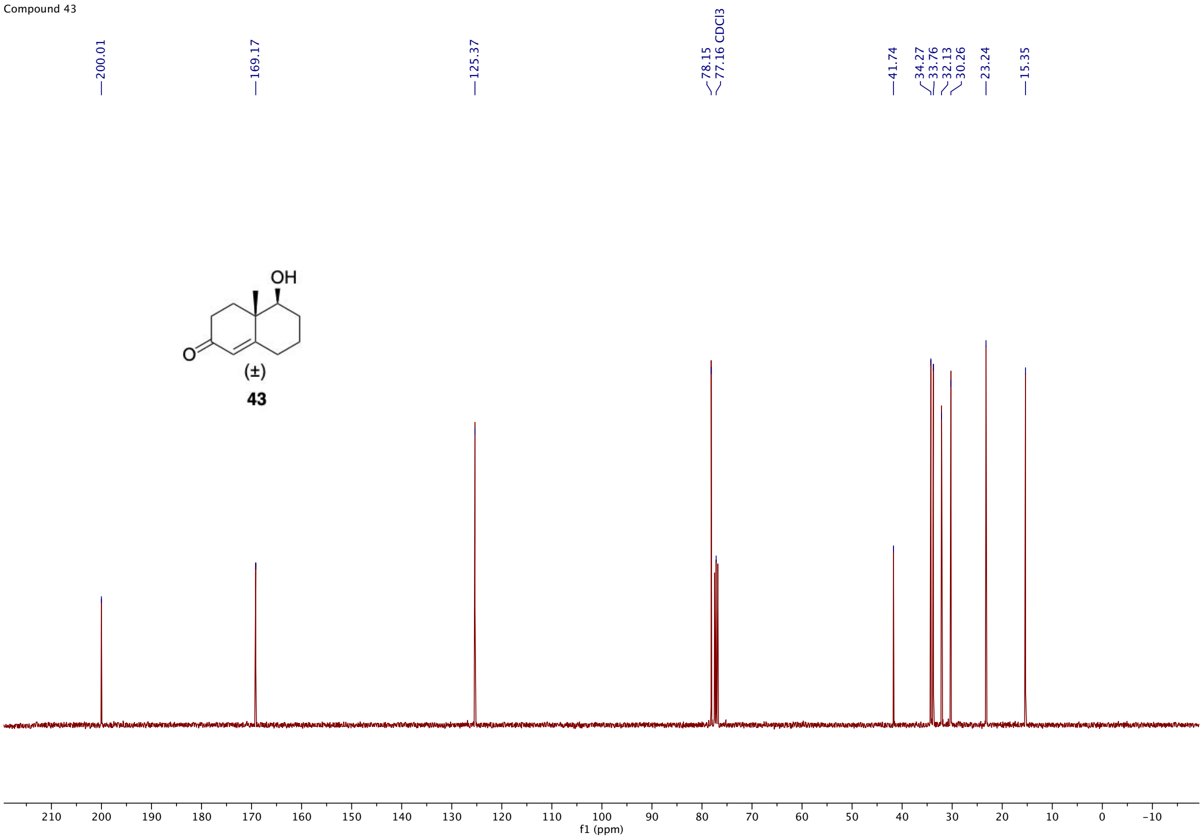

Compound 44

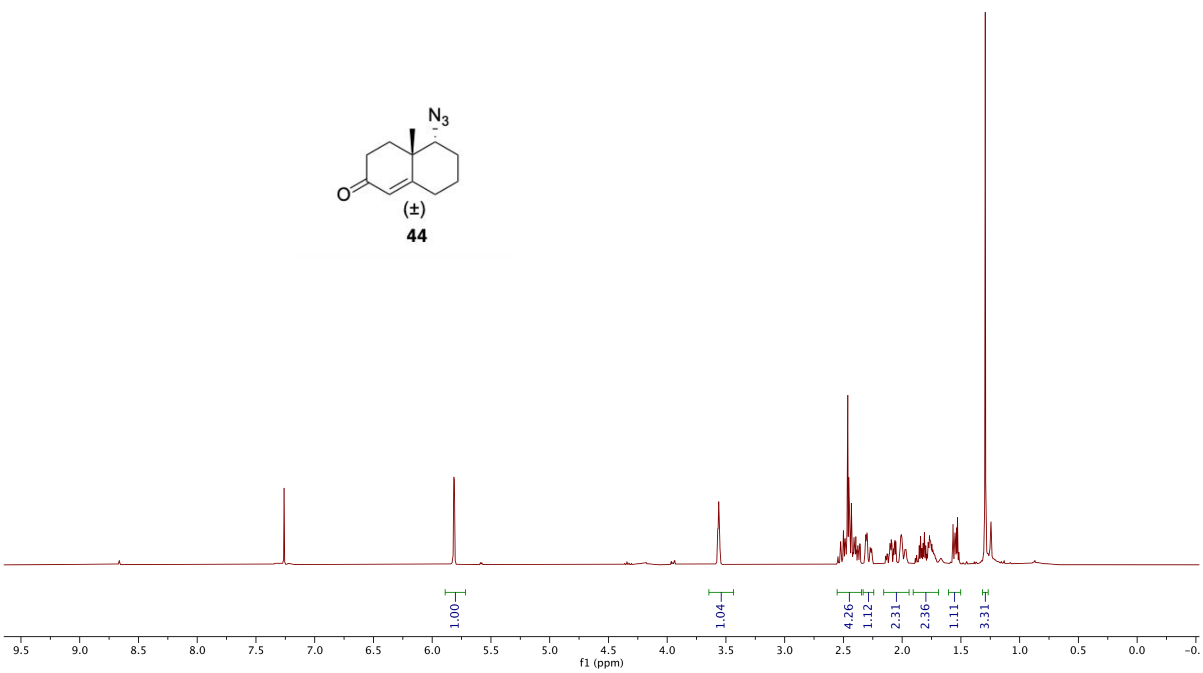

Compound 44

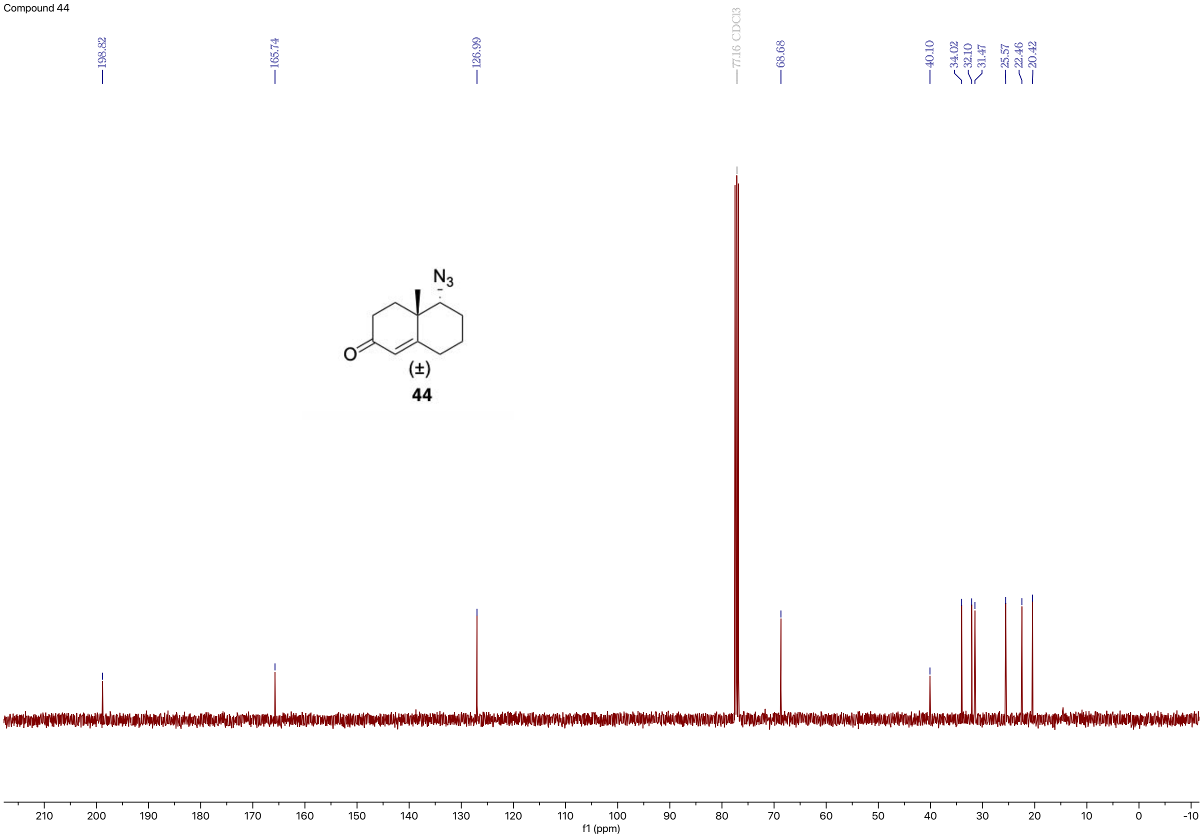

Compound 45

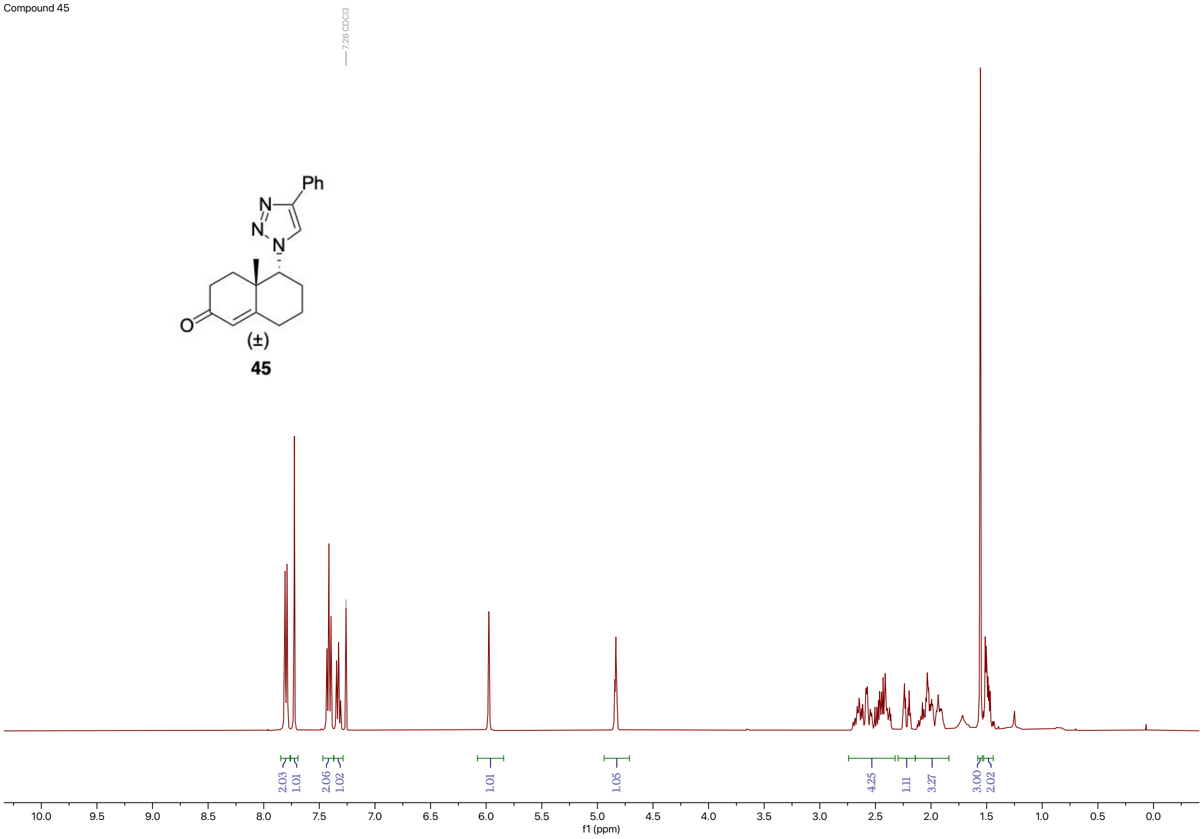

Compound 45

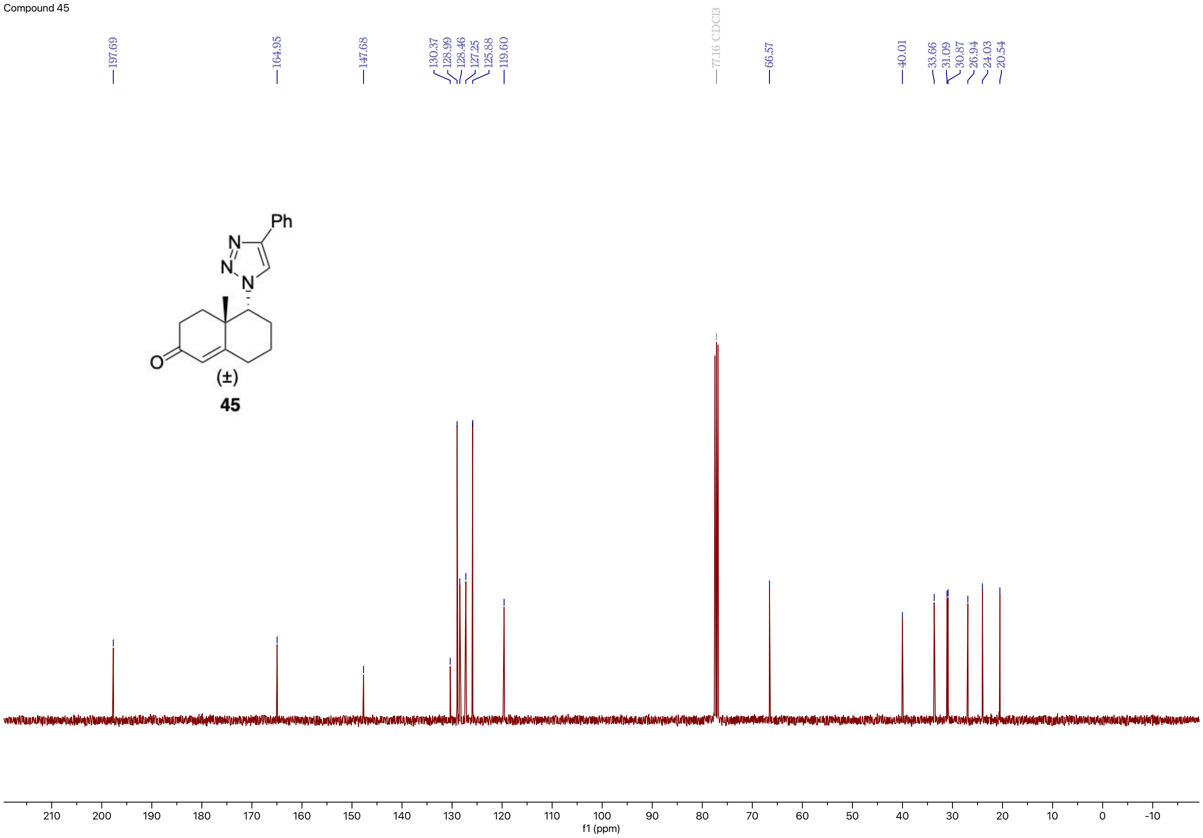

Elemental Composition Report

Single Mass Analysis
Tolerance = 1000.0 PPM / DBE: min = 0.0, max = 50.0
Element prediction: Off
Number of isotope peaks used for i-FIT = 3

Monoisotopic Mass, Even Electron Ions
108 formula(e) evaluated with 49 results within limits (up to 5 closest results for each mass)
Elements Used:
C: 5-50 H: 5-72 N: 1-7 O: 1-5

Compound 45 19 (0.352) AM (Cen,4, 9.00, Ar,6000.0,294.94,0.80,LS 10); Cm (16:23)
1: TOF MS ES+

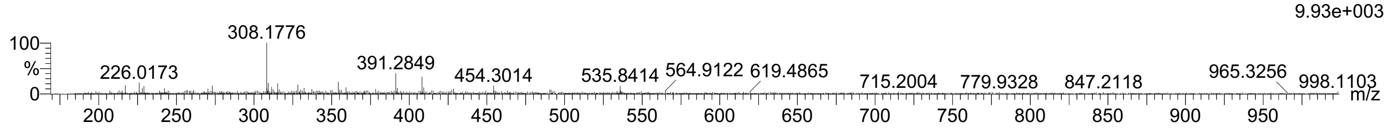

		mDa	PPM	DBE	i-FIT	Formula
Minimum:				0.0		
Maximum:		5.0	1000.0	50.0		

Mass	Calc. Mass	mDa	PPM	DBE	i-FIT	Formula
308.1776	308.1763	1.3	4.2	10.5	57.9	C19 H22 N3 O
	308.1723	5.3	17.2	6.5	119.2	C14 H22 N5 O3
	308.1835	-5.9	-19.1	6.5	135.0	C13 H22 N7 O2
	308.1862	-8.6	-27.9	5.5	76.7	C17 H26 N O4
	308.1682	9.4	30.5	2.5	286.3	C9 H22 N7 O5

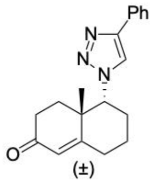

(±)
45

UPLC trace of compound 45

Page 1

Vial:1:19
Date:30-Jul-2009

ID:
Time:12:54:41

Mobile Phase A (10 mM ammonium bicarbonate in water), mobile Phase B (ACN), a flow rate of 0.6 mL/min, injection volume 7.5 µL, run time 6.0 min

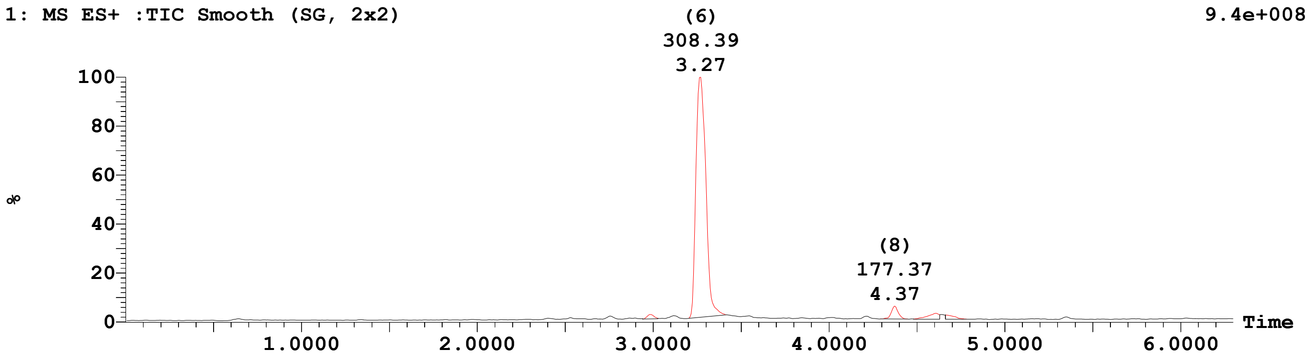

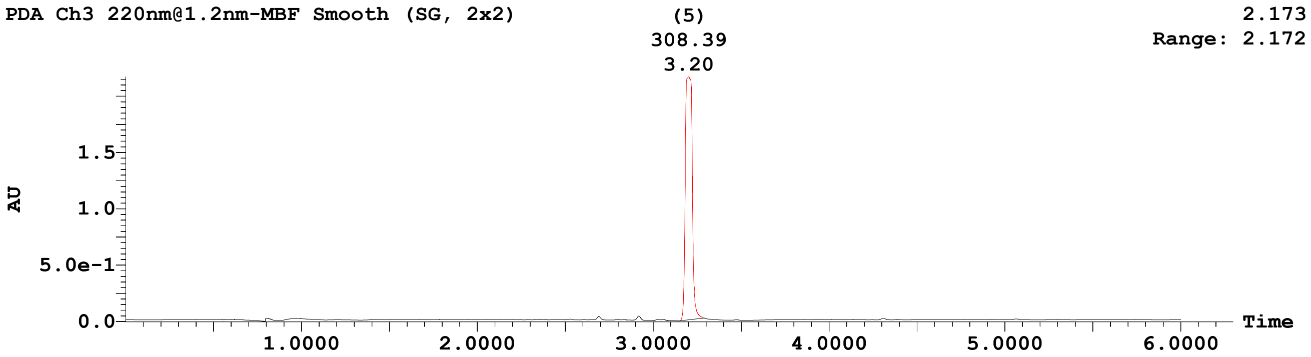

Peak Number	Compound	Time	AreaAbs	Area %Total	Width	Height	Mass Found
5		3.20	9.84e+004	100.00	0	2e+006	

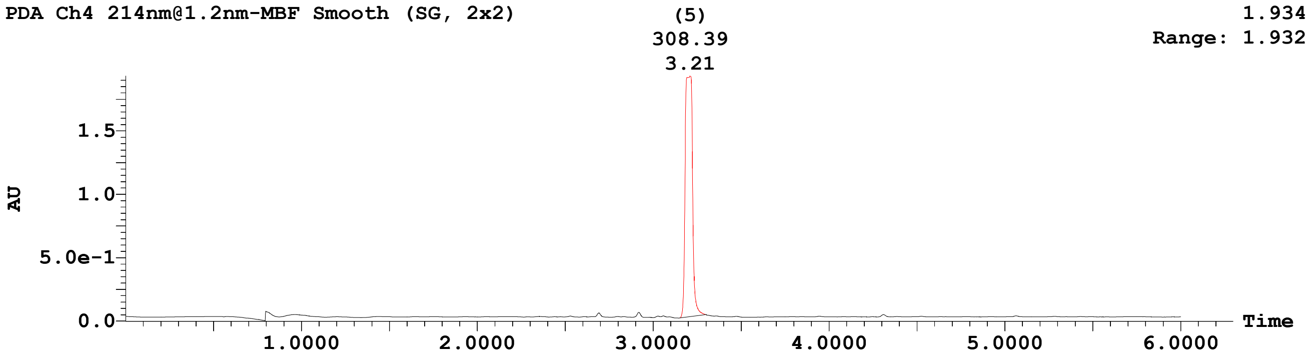

Peak Number	Compound	Time	AreaAbs	Area %Total	Width	Height	Mass Found
5		3.21	9.15e+004	100.00	0	2e+006	
